# Solution-processed organic infrared photodetectors operating beyond 1.4 μm for sensitive environmental monitoring

**DOI:** 10.1126/sciadv.aeg4558

**Published:** 2026-10-07

**Authors:** Yifei Geng, Tengfei Li, Zhenzhen Zhang, Huiqing Hou, Yuze Lin

**Affiliations:** ^1^ Beijing National Laboratory for Molecular Sciences, CAS Key Laboratory of Organic Solids, Institute of Chemistry, Chinese Academy of Sciences, Beijing 100190, China.; ^2^ University of Chinese Academy of Sciences, Beijing 100049, China.

## Abstract

Photon energies in the short-wave infrared (SWIR) beyond 1.4 μm enable access to vibrational-overtone signatures of O–H, C–H, and N–H bonds in pollutants and waste materials, offering an imaging-based nondestructive approach for environmental monitoring. However, current SWIR imaging technologies rely on costly epitaxial semiconductors incompatible with monolithic integration, limiting consumer-grade deployment. Here we report a cost-effective organic SWIR imager with response beyond 1.4 μm, integrating high-detectivity organic photodetectors (OPDs) with amorphous silicon thin-film transistors via solution processing. The developed highly-ordered SWIR organic semiconductors with (halogenated) thiophene-fused quinoid terminals exhibit ultra-narrow optical bandgaps of 0.68–0.94 eV and Urbach energies down to 23 meV. The SWIR OPDs achieve spectral response from 0.3 μm to 1.5–1.7 μm with responsivities up to ∼0.1 A W^−1^ and specific detectivities of 2.03–4.60 × 10^10^ Jones at 1.4 μm. The active-matrix SWIR imagers achieve accurate oil-leak monitoring in simulated seawater (R^2^ = 0.993–0.997) and reliable waste-textile classification (silhouette score = 0.824), demonstrating their capabilities in environmental monitoring.

## INTRODUCTION

Short-wave infrared (SWIR) photodetection beyond 1.4 μm enables precise, rapid, and nondestructive monitoring of environmental pollutants by exploiting the moderate first-overtone absorptions of O–H, N–H, and C–H bonds common in pollutants and waste materials (*1*, *2*), offering deeper penetration depths than the strong mid-infrared fundamental absorptions while providing stronger signals than the weak second-overtones below 1.4 μm (Fig. 1A) (*3*), and surpassing traditional time-consuming and destructive methods reliant on complex sample pre-treatment or manual sorting (Fig. 1B) (*4*). However, current inorganic SWIR sensors, like InGaAs and Ge, rely on costly epitaxial growth and complex flip-chip bonding for integration with readout circuits (*5*, *6*), limiting their low-cost consumer-grade applications, particularly in less-developed regions.

**Fig. 1.**
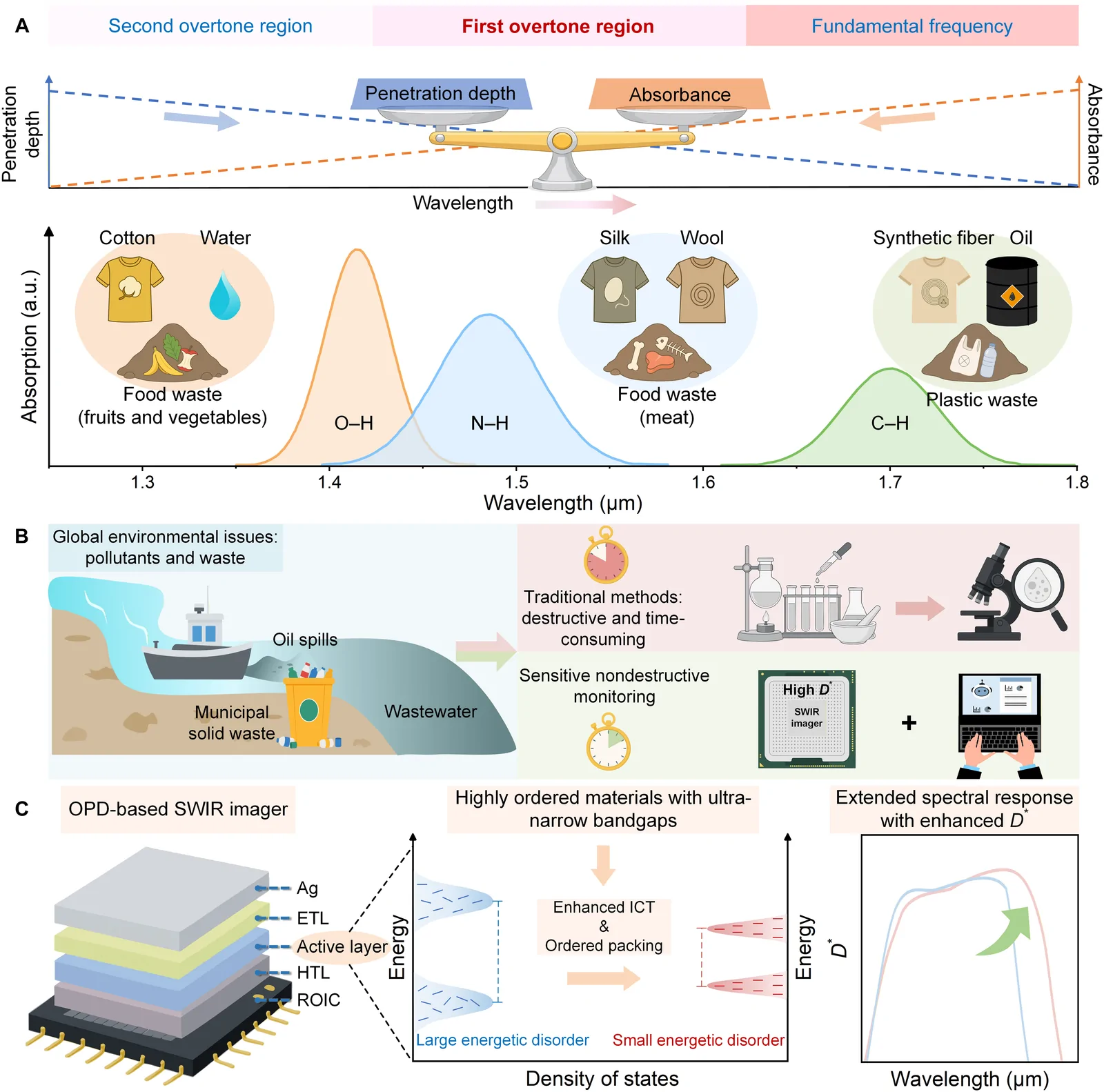
Active-matrix organic SWIR imager for nondestructive environmental monitoring. (**A**) Schematic illustrating the trade-off between penetration depth and absorbance across vibrational spectral regions, highlighting that first-overtone absorptions of O–H, N–H, and C–H bonds offer a balanced, moderate absorption strength with sufficient penetration depth for nondestructive sensing, in contrast to the weak signals in the 2nd-overtone region and the shallow penetration depth of strong fundamental absorptions, together with representative pollutants and waste materials containing these functional groups. (**B**) Improperly treated wastewater discharge and inadequate management of urban solid waste cause severe environmental pollution, posing substantial threats to human health. Portable OPD-based SWIR sensor system with high D* enables nondestructive pollutants and waste monitoring, offering advantages over traditional destructive and time-consuming methods. (**C**) The OPD device consists of a stacked ITO/hole transport layer (HTL)/active layer/electron transport layer (ETL)/Ag structure and is integrated with the readout integrated circuit (ROIC) to form an OPD-based SWIR sensor. The synergistic enhancement of ICT and packing ordering in the active-layer materials leads to reduced bandgap and minimized energetic disorder, thereby enabling OPDs with higher specific detectivity and broader spectral response.

Solution-processed emerging semiconductors offer a cost-effective route to SWIR sensor systems via facile fabrication (*7*–*12*), among which organic semiconductors outperform quantum dots in free of heavy metal toxicity, structural diversity and tunable optoelectronic properties (*13*, *14*). However, due to the lack of organic semiconductors comprising both ultra-narrow optical bandgap (*E*_g_^opt^ < 0.9–1.0 eV) (*15*, *16*) and long-range ordered packing (Fig. 1C) (*17*, *18*), the reported organic photodetectors (OPDs) operating beyond 1.4 μm are scarce and generally exhibit limited responsivity (*R* ≤ 0.05 A W^−1^) and/or specific detectivity (D* ≤ 10^8^–10^9^ Jones) (*19*–*21*), lagging far behind those (D* ≥ 10^10^ Jones) inorganic photodetectors. Therefore, highly ordered organic photoactive materials with ultra-narrow bandgaps are desired for consumer-grade sensitive SWIR imagers with extended spectral response for wide-scale applications (*22*, *23*), such as efficient nondestructive pollution and waste monitoring.

Here, we demonstrate an active-matrix organic SWIR imager operating beyond 1.4 μm by monolithically integrating solution-processed OPDs based on the developed SWIR materials, onto amorphous silicon (a-Si) thin-film transistor (TFT) backplane, and thereby enable sensitive environmental monitoring. Highly ordered SWIR organic semiconductors employing (halogenated) thiophene-fused quinoid terminals are rationally designed and synthesized. These terminal blocks possess high electronegativity and uneven electrostatic potential, leading to *E*_g_^opt^ as narrow as 0.68–0.94 eV and Urbach energy (*E*_U_) down to ∼23 meV of organic semiconductors. The optimized SWIR OPDs response to 1.5–1.7 μm with the high *R* up to ∼0.1 A W^−1^ and D* of 2.03–4.60 × 10^10^ Jones at 1.4 μm. Additionally, our devices achieve a linear dynamic range (LDR) of 149 dB, which exceeds that of InGaAs photodetector (132 dB) and those reported for SWIR OPDs with response beyond 1.4 μm. The resulting active-matrix SWIR imagers enable accurate oil-leak monitoring, achieving reliable and robust monitoring across the full concentration range (R^2^ > 0.99) while attaining single-pixel spatial resolution with detecting oil droplets as small as ∼1 mm in diameter (signal-to-noise ratio (SNR) = 4.59). For textile-waste identification, the system delivers robust clustering between natural and synthetic fibers, yielding a silhouette score of 0.824 (range −1 to 1, with values closer to 1 indicating optimal quality), while maintaining reliable detection across a broad range of fabric thicknesses, underscoring the suitability of this approach for practical textile-waste sorting.

## RESULTS

### Design of SWIR organic semiconductors

Sulfur atoms exhibit a much higher polarizability (∼3.4 Å^3^) than carbon atoms (∼1.2 Å^3^), owing to their larger atomic radii and more spatially extended p orbitals (*24*, *25*) (Fig. 2A and fig. S1), which is expected to facilitate more effective delocalization of excess negative charges within the π-conjugated system and further enhance the intramolecular charge transfer (ICT) effect. Additionally, the electron clouds of highly polarizable sulfur atoms tend to deform asymmetrically under nearby charge influence, which is likely to induce uneven local charge distributions that potentially enhance non-covalent interactions between sulfur and surrounding atoms, thereby promoting more ordered molecular packing (*26*).

**Fig. 2.**
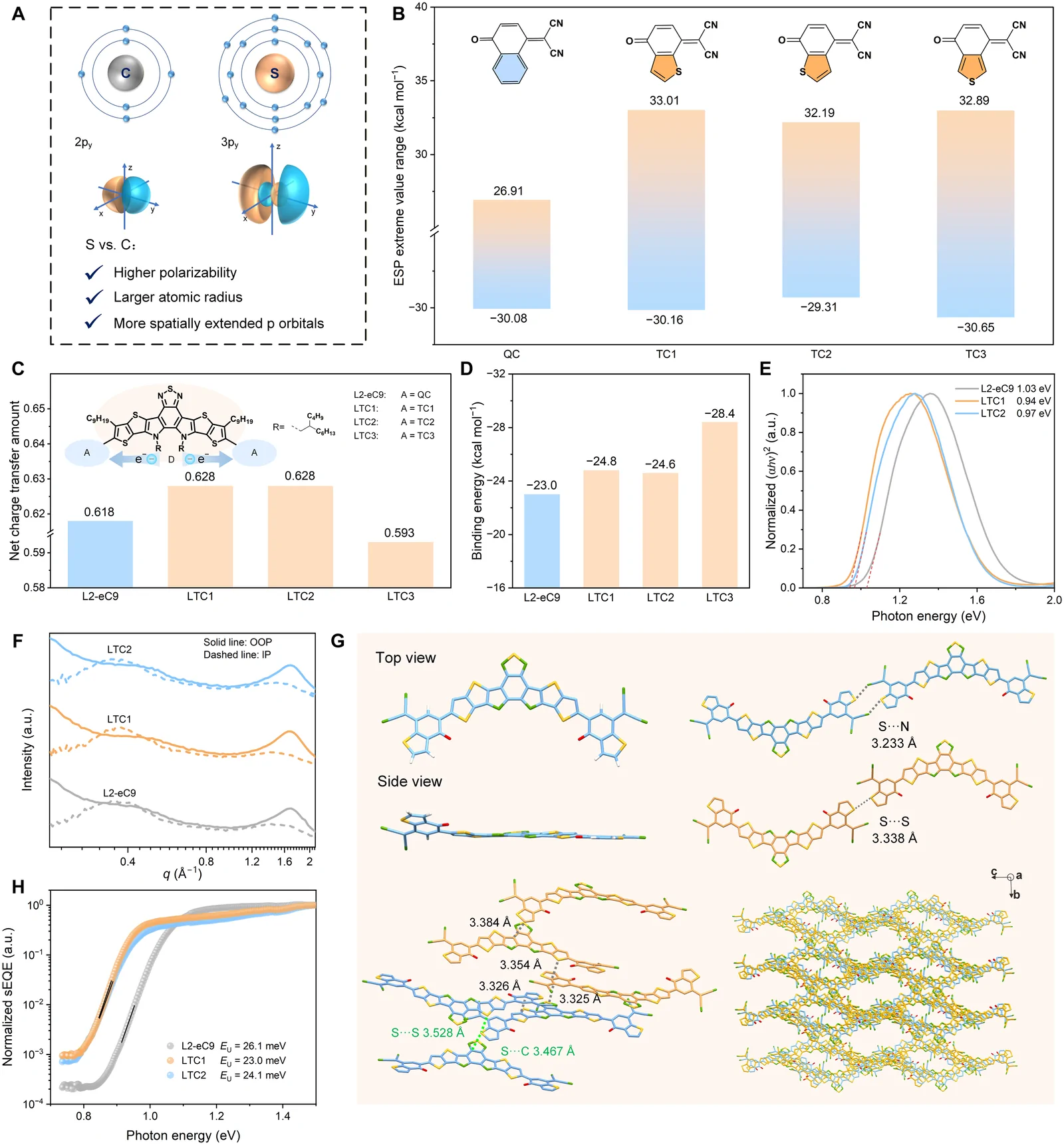
Design and properties of SWIR sensing molecules. (**A**) Atomic structures and electron configurations of C and S, along with their 2p_y_ and 3p_y_ orbitals, respectively. (**B**) Chemical structures and ESP extreme value range of the quinoid terminal groups QC and TCx (x = 1, 2, 3). (**C)** Chemical structures of L2-eC9 and LTCx (x = 1, 2, 3) along with the calculated charge transfer amounts from the D fragment to the A fragment during electronic transition. (**D**) The calculated binding energy of L2-eC9 and LTCx (x = 1, 2, 3) dimers. (**E**) The *E*_g_^opt^ values of L2-eC9, LTC1, and LTC2 derived from thin-film absorption spectra. (**F**) Line-cut profiles of the 2D GIWAXS data in the out-of-plane (OOP) and in-plane (IP) directions for neat films of L2-eC9, LTC1, and LTC2. (**G**) The monomolecular state with a banana-shaped geometry and quasi-planar conformation extracted from the unit cell, and intermolecular stacking modes of LTC1 in single crystals. The 3D interpenetrating network packing structures of LTC1 in single crystals. (**H**) Normalized sEQE spectra and the exponential fitting of *E*_U_ for single-component devices based on L2-eC9, LTC1, and LTC2.

Therefore, we replaced the benzene ring in the strongly electron-withdrawing 2-[4-oxonaphthalen-1(4H)-ylidene]malononitrile (QC) terminal with thiophene to design three terminals (TCx, x = 1, 2, 3, Fig. 2B), whose Mulliken electronegativities (5.61, 5.72, and 5.53 eV) obtained from Density functional theory (DFT) calculations confirm electron-withdrawing strengths comparable to QC (5.60 eV) (*15*). Additionally, as reflected by similar negative electrostatic potential (ESP) minima (−30.08 kcal mol^−1^ for QC; −29.31 to −30.65 kcal mol^−1^ for TCx) but notably higher positive ESP maxima (26.91 kcal mol^−1^ for QC; 32.19 to 33.01 kcal mol^−1^ for TCx) (Fig. 2B and fig. S2), compared to the QC unit, the TCx end groups exhibit more uneven local charge distributions around the sulfur atoms as expected.

Based on TCx as acceptor (A) units, A-D-A type SWIR sensing materials LTCx are designed using dithienothiophen[3,2-b]pyrrolobenzothiadiazole (BTP) as the donor (D) unit, and DFT calculations reveal that LTC1 and LTC2 possess similar HOMO levels (−5.35 and −5.34 eV) but deeper LUMO levels (both −4.02 eV) than the QC analogue L2-eC9 (−5.33 eV/−3.91 eV), resulting in smaller transport gaps (1.32–1.33 eV vs. 1.42 eV) (fig. S3). This can be attributed to enhanced ICT arising from the high polarizability of sulfur, which improves charge accommodation and electron delocalization along the conjugated backbone. The net transferred charges (*Q*_CT_) from the D fragment to the A fragment during electronic excitation are 0.628 electrons for both LTC1 and LTC2, higher than that of L2-eC9 (0.618 electrons) (*27*) (Fig. 2C), confirming the enhanced ICT effect in LTC1 and LTC2. Interestingly, LTC3 differs from LTC1 and LTC2 because the distinct thiophene orientation weakens the ICT effect (*28*) (detailed analysis can be found in figs. S3 to S5 and note S1). Furthermore, LTCx dimers exhibit more negative BSSE-corrected binding energies (*29*, *30*) (−24.6 to −28.4 kcal mol^−1^) than L2-eC9 (−23.0 kcal mol^−1^) (Fig. 2D and fig. S6), indicating stronger intermolecular stacking that favors enhanced molecular crystallinity and charge transport.

### Properties of SWIR organic semiconductors

According to the synthetic routes outlined in fig. S7, LTC1 and LTC2 featuring narrower bandgap than LTC3 were synthesized with terminal group of TC1 and TC2, respectively. The ultraviolet-visible-near infrared (UV-vis-NIR) absorption spectra show that in solution (∼10^−6^ M CHCl_3_), LTC1 and LTC2 exhibit nearly identical long-wavelength absorption peak (λ_max_) values at 940 nm and 947 nm, redshifted by 72 and 79 nm compared with L2-eC9 (868 nm), consistent with the enhanced ICT predicted by DFT, while in thin films their λ_max_ values further shift to 1080 and 1060 nm versus 958 nm for L2-eC9 (fig. S8). Consequently, the films of TCx-based materials exhibit significantly larger bathochromic shifts compared to their dilute solutions, featuring Δλ_max_ and corresponding energy differences of 140 nm and 0.171 eV for LTC1, and 113 nm and 0.139 eV for LTC2, respectively, exceeding those of L2-eC9 (90 nm and 0.134 eV). Among them, LTC1 exhibits the largest Δλ_max_ and corresponding energy differences, indicating stronger intermolecular interactions and enhanced molecular packing in the LTC1 solid state (discussed further below), which is consistent with its highest dimer binding energy observed in theoretical calculations. As illustrated in Fig. 2E, the *E*_g_^opt^s of LTC1 and LTC2 are estimated to be 0.94 and 0.97 eV, respectively, which are smaller than L2-eC9 (1.03 eV). Meanwhile, ultraviolet photoelectron spectroscopy and low-energy inverse photoemission spectroscopy measurements further show that deeper LUMO levels of LTCx than L2-eC9 (−4.29/−4.30 eV vs. −4.18 eV) with similar HOMOs (−5.52 eV vs. −5.48 eV) (fig. S9) account for the reduced *E*_g_^opt^. Grazing-incidence wide-angle x-ray scattering (GIWAXS) analysis of L2-eC9, LTC1, and LTC2 films (Fig. 2F, fig. S10, and table S1) shows that all adopt a primarily face-on orientation. Compared with L2-eC9 (19.0%), LTC1 and LTC2 films exhibit reduced amorphous content in the π-π stacking direction (11.9% and 12.7%), demonstrating that sulfur incorporation promotes more ordered π-π stacking. In addition, their lamellar (100) diffraction peaks at 0.367–0.375 Å^−1^ with longer crystalline coherence lengths (CCLs) (43.2 and 36.0 Å vs. 32.3 Å for L2-eC9) indicate improved lamellar ordering, which is attributed to stronger sulfur-mediated intermolecular interactions (*26*) as supported by single-crystal analysis and correlates with the larger Δλ_max_ observed in LTC1 and LTC2 films.

High-quality single crystals of LTC1 were obtained via solvent diffusion, and characterized by single-crystal x-ray diffraction (table S2). In contrast, single crystals of LTC2 could not be obtained, which is likely attributed to its higher degree of structural disorder compared to LTC1, consistent with its relatively smaller CCL observed in GIWAXS analysis. LTC1 crystallizes in a monoclinic system (space group P2_1_/c) with two distinct molecular conformations in the unit cell. As shown in Fig. 2G, the molecule adopts a nearly planar conformation, stabilized by intramolecular S⋯O interactions between the BTP core and the terminal group, with an average interaction distance of 2.67 Å, forming interlocking structural features. Along the molecular backbone direction, adjacent molecules exhibit non-covalent N⋯S and S⋯S interactions, with distances of 3.233 Å and 3.338 Å, respectively, both shorter than the corresponding van der Waals radii sums (3.35 Å for N⋯S and 3.60 Å for S⋯S), leading to a wavelike arrangement within the planar layer. LTC1 exhibits multiple dimer configurations featuring head-to-tail and head-to-core π-π stacking modes with interlayer distances of 3.325–3.384 Å, accompanied by non-covalent S⋯S and S⋯C interactions (3.528 Å and 3.467 Å, respectively) mediated by the terminal sulfur atoms. These interactions yield a three-dimensional intermolecular network with ordered molecular stacking, which is advantageous for facilitating efficient multidirectional charge transport (*31*), consistent with the superior crystallinity observed in LTC1 films. A packing coefficient of 68.8% was calculated for the unit cell of LTC1, representing the ratio of the sum of molecular volumes to the cell volume and reflecting the tightness of intermolecular packing (*32*), which is one of the highest values reported among high-performance organic photovoltaic materials to date (*33*), indicating that LTC1 exhibits dense molecular packing and a compact crystal structure, potentially promoting higher electron mobility. Accordingly, LTC1 exhibits the highest electron mobility (μ_e_ = 5.6 × 10^−4^ cm^2^ V^−1^ s^−1^), followed by LTC2 (μ_e_ = 3.5 × 10^−4^ cm^2^ V^−1^ s^−1^), with both outperforming L2-eC9 (μ_e_ = 2.5 × 10^−4^ cm^2^ V^−1^ s^−1^), as determined by space-charge-limited current (SCLC) measurements (fig. S11). The *E*_U_ values derived from highly sensitive external quantum efficiency (sEQE) measurements were 23.0 meV for LTC1 and 24.1 meV for LTC2, both lower than that of L2-eC9 (26.1 meV) (Fig. 2H), with the lowest value of LTC1 arising from enhanced intermolecular packing, consistent with previous analyses and ranking among the smallest reported for organic semiconductors (*34*).

### Device performance of SWIR OPDs

Photodiode-type SWIR OPDs with an inverted structure were fabricated, using bulk heterojunction active layers of PTB7-Th (HOMO: −5.05 eV, LUMO: −3.40 eV; fig. S12) (*35*) blended with LTC1, LTC2, or L2-eC9 to ensure efficient exciton dissociation via well-aligned energy levels (*36*). As shown in Fig. 3A, PTB7-Th: LTC1 and PTB7-Th: LTC2 devices exhibit broader spectral responses (0.3–1.5 μm) than PTB7-Th: L2-eC9 (0.3–1.3 μm), with PTB7-Th: LTC1 achieving the highest *R* values with a peak of 0.097 A W^−1^ at 1.06 μm, while PTB7-Th: LTC2 and PTB7-Th: L2-eC9 reach maximum *R* values of 0.087 A W^−1^ at 1.06 μm and 0.093 A W^−1^ at 1.04 μm, respectively.

**Fig. 3.**
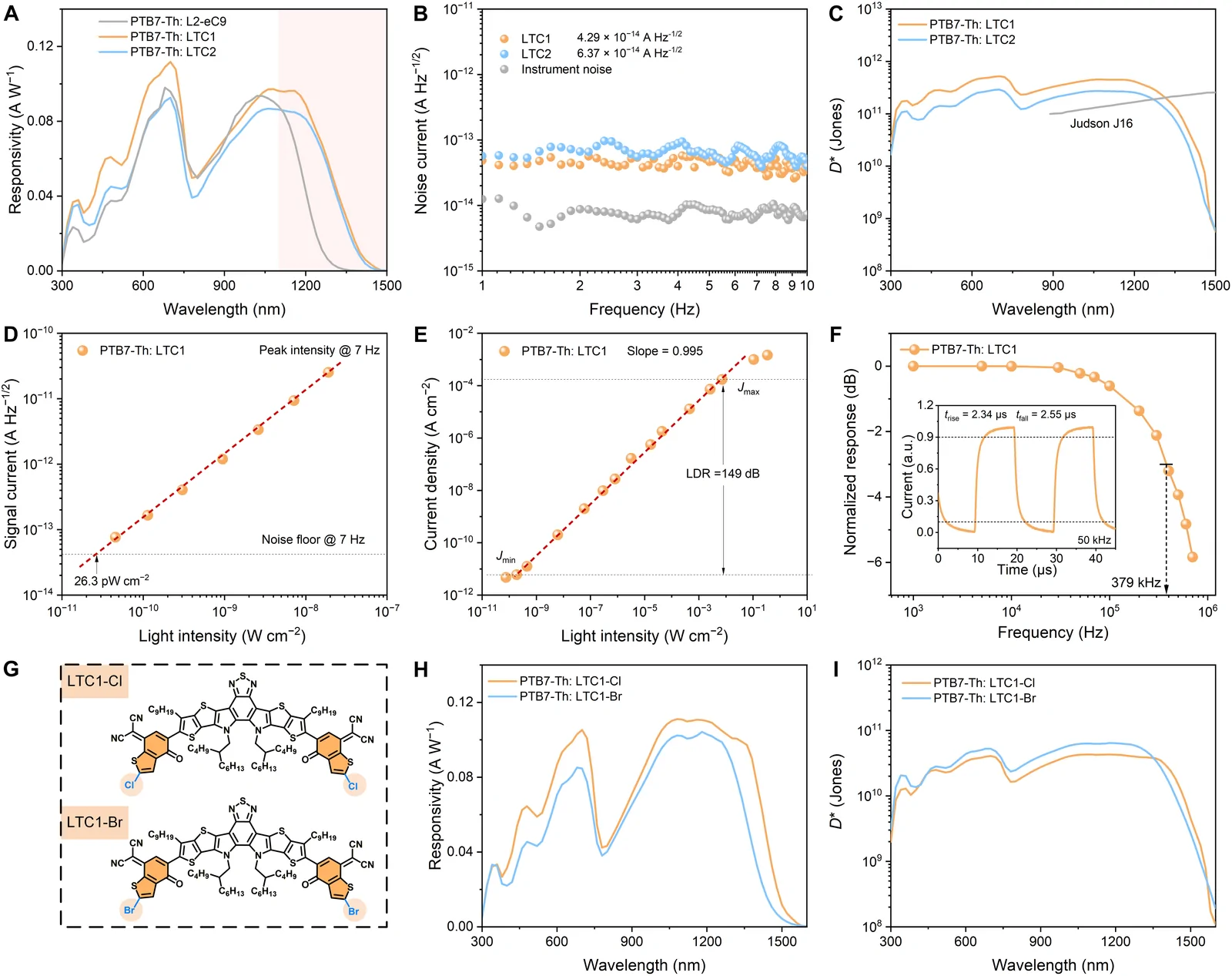
Device performance of SWIR OPDs. (**A**) The *R* curves of SWIR OPDs based on PTB7-Th: L2-eC9, PTB7-Th: LTC1, and PTB7-Th: LTC2 under zero bias. (**B**) The measured noise currents of SWIR OPDs based on PTB7-Th: LTC1 and PTB7-Th: LTC2 under zero bias. (**C**) The D* curves of SWIR OPDs based on PTB7-Th: LTC1 and PTB7-Th: LTC2 under zero bias. The D* curves of commercial Ge photodetectors (Judson J16). (**D**) The peak signal currents under 1.3 μm light illumination modulated at 7 Hz as a function of light intensities for PTB7-Th: LTC1-based SWIR OPD. (**E**) The LDR measurement under illumination at 1.3 μm with various light intensities for PTB7-Th: LTC1-based SWIR OPD. (**F**) The cut-off frequency of SWIR OPDs based on PTB7-Th: LTC1 under illumination at 1.3 μm, with the inset showing the response time of the PTB7-Th: LTC1-based OPD under 1.3 μm LED illumination. (**G**) Molecular structures of LTC1-Cl and LTC1-Br. (**H**) The *R* curves of SWIR OPDs based on PTB7-Th: LTC1-Cl, and PTB7-Th: LTC1-Br under zero bias. (**I**) The D* curves of SWIR OPDs based on PTB7-Th: LTC1-Cl, and PTB7-Th: LTC1-Br under zero bias.

The D* is a critical figure of merit for OPD devices, quantifying their ability to detect faint light signals (*14*), and is calculated from the *R* and the noise current (*i*_n_) using the following equation

D∗=RABin=ANEP (1)

where *A* is the device area (0.04 cm^2^ in this work), *B* is the electrical measurement bandwidth, and the NEP is the noise-equivalent power. As depicted in Fig. 3B, the noise current at zero bias exhibits frequency independence across the tested frequency range, and the average *i*_n_ values of LTC1- and LTC2-based OPDs are measured as 4.29 × 10^−14^ A Hz^−1/2^ and 6.37 × 10^−14^ A Hz^−1/2^, respectively, lower than those reported for OPDs with comparable response ranges (*19*, *37*). Previous studies have demonstrated that the *i*_n_ of OPDs in photovoltaic mode strongly correlates with the effective bandgap (*E*_eff_, defined as the difference between the LUMO of acceptor and the HOMO of donor) and the bandtail disorder spread (*38*). Notably, the deeper Fermi levels of LTC1 (−4.56 eV), LTC2 (−4.79 eV), and L2-eC9 (−4.71 eV) relative to that of PTB7-Th (−4.46 eV) lead to vacuum level shifts at the donor–acceptor interfaces upon contact (*35*, *39*). As a result, the actual *E*_eff_ in the PTB7-Th: LTC1 is derived as 0.86 eV, smaller than that of PTB7-Th: LTC2 films (1.08 eV) (fig. S13). Meanwhile, the PTB7-Th: LTC1-based OPD exhibits an obviously lower *E*_U_ value of 23.5 meV compared to 26.3 meV for PTB7-Th: L2-eC9, indicating reduced bandtail disorder. In contrast, PTB7-Th: LTC2 -based OPD shows a higher *E*_U_ of 26.2 meV relative to PTB7-Th: LTC1 (fig. S14), consistent with its elevated noise current. Consequently, the D* values for LTC1- and LTC2-based devices, determined by *R* and *i*_n_, exceed 10^11^ Jones across the 0.3 to ∼1.4 μm range. Notably, LTC1-based devices achieve a peak D* of 4.53 × 10^11^ Jones at 1.06 μm, significantly surpassing commercial Ge detectors at wavelengths below 1.3 μm and representing one of the highest reported values among OPDs responsive beyond 1.4 μm (Fig. 3C and table S3). Considering that OPDs generally operate under reverse bias in practical applications, we further evaluated the performance of the LTC1- and LTC2-based devices under different reverse biases (figs. S15 and S16). Although the enhancement in responsivity under reverse bias, the concomitant increase in *i*_n_ values results in an approximately 1 ∼ 2 orders reduction in detectivity for the OPDs relative to those operating at zero bias.

The PTB7-Th: LTC1-based OPDs show extrapolated detection limits of 16.2 and 26.3 pW cm^−2^ under 1.05 and 1.3 μm illumination at 0 V, slightly lower than those of LTC2-based devices (27.9 and 37.8 pW cm^−2^) and in good agreement with calculated values (11.2 and 21.4 pW cm^−2^), with the detection limit at 1.3 μm shifting to 191 pW cm^−2^ (calculated: 186 pW cm^−2^) under −0.5 V bias (Fig. 3D and figs. S17 to S19). The LDR reaches 149 dB at 0 V and remains 130 dB under −0.5 V bias, both comparable to or exceeding that of commercial InGaAs detectors (132 dB) (Fig. 3E and fig. S20) (*40*). Moreover, the devices deliver fast photoresponse with *t*_rise_/*t*_fall_ of 2.34/2.55 μs and a − 3 dB cut-off frequency of 379 kHz at 0 V, outperforming LTC2- and L2-eC9-based devices, and further accelerate to 1.37/1.39 μs and 838 kHz under −0.5 V bias (Fig. 3F and figs. S21 and S22).

To elucidate the origin of the superior performance, the morphology of the blended films and the carrier dynamics of the devices were further investigated. GIWAXS reveals face-on orientation for all blended films, with PTB7-Th: LTC1 and PTB7-Th: LTC2 displaying longer lamellar CCLs and markedly lower amorphous fractions along the π-π direction than L2-eC9 (figs. S23, S24, and table S4), indicating that sulfur incorporation facilitates stronger intermolecular interactions and more ordered π-π stacking (note S2). Correspondingly, PTB7-Th: LTC1 devices exhibit the lowest trap densities and more balanced, faster charge transport (note S3 and figs. S25 and S26), which suppress recombination and enhance charge extraction, as evidenced by the lowest bimolecular recombination rate coefficient and the shortest carrier extraction times from transient photovoltage and transient photocurrent measurements (note S4 and fig. S27). Collectively, these morphological ordering and charge dynamics underpin the highest responsivity of PTB7-Th: LTC1 devices.

### Extended-response OPD-based SWIR imager

Building on the thiophene-fused terminals, we employed a halogenation strategy to further strengthen the electron-withdrawing character of the terminal groups (*16*, *41*) and extend the photoresponse range of organic semiconductors over 1.5 μm. The α-position of the thiophene ring in the TC1 terminal was brominated and chlorinated to yield BrTC1 and ClTC1 (fig. S7), which were used to construct electron acceptors LTC1-Br and LTC1-Cl (Fig. 3G). DFT calculations show *Q*_CT_ values of 0.657 and 0.658 e for LTC1-Br and LTC1-Cl, respectively, both higher than LTC1 (0.628 e), confirming enhanced ICT. Consistently, the solution absorption of LTC1-Br and LTC1-Cl are both redshifted to 974 nm (vs. 940 nm for LTC1, fig. S28A), while thin-film absorption maxima reach 1.16 μm (LTC1-Br) and 1.17 μm (LTC1-Cl) (vs. 1.08 μm for LTC1, fig. S28B), with narrower optical bandgaps of 0.89 (LTC1-Br) and 0.87 eV (LTC1-Cl) (vs. 0.94 eV for LTC1, fig. S28C) and extended absorption edges to ∼1.55 μm, attributed to enhanced intermolecular charge-transfer-coupled aggregation. Similar to LTC1 film, LTC1-Cl also exhibits good photostability and thermal stability (fig. S29).

Subsequently, SWIR OPDs based on PTB7-Th: LTC1-Cl and PTB7-Th: LTC1-Br were fabricated and characterized (fig. S30). Both devices exhibited markedly enhanced *R* across a broad spectral range of 0.3–1.6 μm, achieving peak *R* values of 0.111 A W^−1^ and 0.104 A W^−1^ at 1.18 μm for PTB7-Th: LTC1-Cl and PTB7-Th: LTC1-Br, respectively (Fig. 3H). Compared to PTB7-Th: LTC1-Br-based OPDs, PTB7-Th: LTC1-Cl-based OPDs demonstrate consistently higher responsivity, with notable *R* value up to ∼0.1 A W^−1^ at ∼1.4 μm, which is also markedly higher than that of LTC1-based OPDs around 1.4 μm. Furthermore, the average *i*_n_ values for SWIR OPDs based on PTB7-Th: LTC1-Cl and PTB7-Th: LTC1-Br were measured at 5.17 × 10^−13^ A Hz^−1/2^ and 3.23 × 10^−13^ A Hz^−1/2^, respectively (fig. S31). Consequently, SWIR OPDs based on PTB7-Th: LTC1-X exhibit D* values of 2.95 × 10^10^ Jones for LTC1-Cl and 2.03 × 10^10^ Jones for LTC1-Br at 1.4 μm (Fig. 3I). Under reverse bias, the performance variation trends of the PTB7-Th: LTC1-Cl and PTB7-Th: LTC1-Br based OPDs are similar with those of the LTC1-based devices (figs. S32 and S33).

Given their good operational stability similar to that observed for LTC1-based devices, high *R* and D* at 1.4 μm, sufficient LDR of 89 dB at −0.5 V, and fast response times (*t*_rise_ = 1.23 μs and *t*_fall_ = 1.31 μs at −0.5 V) (figs. S34–S36), the solution-processed LTC1-Cl-based OPDs are directly deposited onto a commercial 64 by 64 a-Si TFT backplane with a 500 μm by 500 μm pixel pitch without additional photolithographic patterning to form the active-matrix imager. The a-Si TFT backplane is built on a glass substrate with patterned Mo: Al gate electrodes and scan lines, a SiN_x_ gate insulator, an a-Si semiconductor layer, Mo: Al source and drain electrodes forming bottom-gate staggered TFTs, a SiN_x_ passivation layer, and ITO pixel electrodes contacted to the TFTs through vias in the dielectric. Owing to the scalability of a-Si TFT backplanes and the solution-processable organic OPD layer, the same architecture can be straightforwardly scaled to larger areas and higher pixel densities at much lower cost than complementary-metal-oxide-semiconductor circuits, and it offers a practical solution for wide-scale consumer-grade application of SWIR imaging (*42*). Integrated with a commercial readout system, the resulting 64 by 64 sensor array delivers an average photocurrent of ∼200 nA per pixel under 1.4 μm light-emitting diode (LED) illumination (2 mW cm^−2^) in the absence of ambient light (fig. S37), providing sufficient SWIR signal levels for the following environmental monitoring demonstrations.

### Proof of concept for environmental sensing

#### Oil spill simulation detection

Timely and accurate pollution monitoring is a prerequisite for environmental sustainability, particularly in aquatic and marine systems where oil spills pose severe ecological risks and require rapid source identification to mitigate environmental damage (*43*). The SWIR OPD-based sensor array leverages the strong first-overtone absorption of O–H at ∼1.4 μm and the comparatively weak hydrocarbon absorption at the same wavelength to rapid discriminate of oil-water mixtures. Compared with conventional methods (Fig. 4A), the OPD-based array provides several advantages: relative to satellite or airborne remote sensing, it enables real-time on-site monitoring that avoids the hours-to-days delay in data collection and processing (*44*); compared with acoustic techniques such as multibeam sonar, it offers higher spatial resolution, allowing reliable detection of small oil droplets and thin films that acoustics often under-resolves (*45*); and unlike thermal infrared imaging, which is limited to sea-surface slicks due to water’s strong absorption (*44*, *46*), the SWIR OPD-based arrays enable subsurface and underwater detection.

**Fig. 4.**
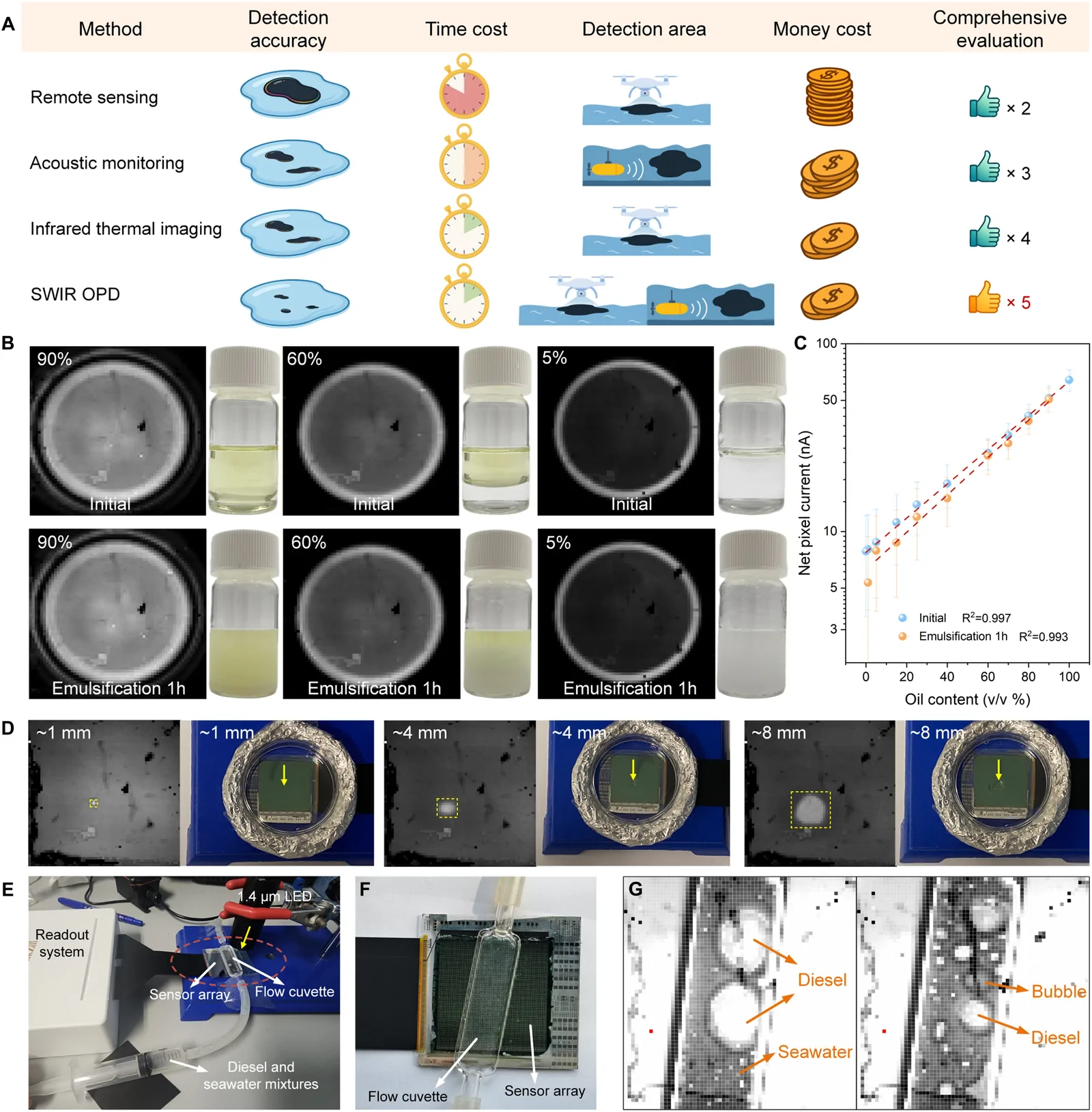
Oil spill simulation detection. (**A**) Comparison of oil spill monitoring methods, including remote sensing, acoustic monitoring, infrared thermal imaging, and the developed OPD-integrated SWIR sensor array. The comprehensive evaluation (indicated by thumbs-up symbols) is based on four key indices relevant to oil spill monitoring. Relative to SWIR OPDs, the high cost of conventional Ge/InGaAs photodetectors limits their accessibility in low- and middle-income countries. (**B**) SWIR imaging of oil-water mixtures with oil fractions of 90%, 60%, and 5% before (also presented in fig. S39) and after 1 h emulsification (also presented in fig. S40), together with optical photographs. The SWIR images were captured from an upward view after the container lid was removed, with an imaging area of 3.2 cm by 3.2 cm. All containers were 20 mL sample vials (body diameter: 2.7 cm) containing 10 mL of liquid. (**C**) Net photocurrent versus oil fraction under 1.4 μm LED illumination for the initial mixture and after 1 h emulsification, with corresponding fitted curves. Error bars represent the standard error of the mean. Quantitative analysis uses the average net photocurrent from an 11 by 12 pixel matrix in the central region of the sample image. (**D**) Detection of oil droplets with diameters of ∼1 mm, ∼4 mm, and ∼ 8 mm under 1.4 μm illumination, shown with corresponding imaging and optical photographs (also presented in fig. S41). The imaging area is 3.2 cm by 3.2 cm, and the samples are contained in culture dishes (diameter: 6 cm), each holding 10 mL of solution. (**E**) Illustration of the active-matrix SWIR imaging system using an OPD-integrated TFT sensor array as the key element. (**F**) A photograph of oil spill monitoring employing the active-matrix OPD-based SWIR imaging system. (**G**) Image sensing of oil spill monitoring under 1.4 μm LED illumination without ambient light. The imaging area is 3.2 cm by 3.2 cm.

To evaluate the SWIR OPD-based sensor array for detecting oil spills in seawater, we selected commercial No. 0 diesel from a local gas station, which is a common component of marine oil spills, and seawater from the Bohai Sea to simulate oil spill scenarios (fig. S38). Under 1.4 μm LED illumination, the net photocurrent exhibited an exponential dependence on oil fraction (Fig. 4, B and C and fig. S39), which can be described by

$$J_{P}\left(\phi_{\text{oil}}\right)=J_{0}e^{k\phi_{\text{oil}}}$$ (2)

where *J*_P_(ϕ_oil_) is the net photocurrent at oil fraction ϕ_oil_, *J*_0_ is the extrapolated zero-content photocurrent, and *k* is the exponential growth factor reflecting the sensitivity to oil concentration. Fitting yielded *k* = 2.10 (per v/v) and *J*_0_ = 7.73 × 10^−9^ A, with a deviation of only 1.5% from the measured blank of 7.85 × 10^−9^ A and an excellent coefficient of determination (R^2^ = 0.997), demonstrating the high measurement accuracy of the sensor array for oil-leak detection across the full concentration range (0–100%) in seawater. In real seawater environments, oil slicks inevitably undergo emulsification over time, driven primarily by wave-induced mechanical agitation together with the surface-active components naturally present in crude oil. To mimic this process, simulated oil-water emulsification was conducted for 1 h, after which the net photocurrents at identical oil fractions were consistently lower than those of the non-emulsified mixtures (Fig. 4, B and C and fig. S40), owing to enhanced light scattering and turbidity from the dispersed oil droplets that reduced effective light transmission. At oil fractions above 5%, the photocurrent-concentration relationship can be well fitted by the exponential model with an exponential growth factor of *k* = 2.29 and an extrapolated baseline photocurrent of *J*_0_ = 5.34 × 10^−9^ A, whereas at very low oil fractions (ϕ_oil_ = 0.01) the measured photocurrent deviates from the fitted trend. These results demonstrate that the SWIR OPD-based sensor array maintains high accuracy and sensitivity for oil-leak detection even under realistic emulsified seawater conditions, as confirmed by the excellent goodness of fit (R^2^ = 0.993). In addition, oil droplets of different sizes ranging from ∼1 mm to ∼12 mm in diameter were detected (Fig. 4D and fig. S41), and even for the smallest droplets (∼1 mm), a SNR of 4.59 was obtained, corresponding to an effective spatial resolution of ∼1 mm.

To further emulate realistic flow-state oil spill scenarios, we established a controlled water-flow channel with continuous introduction of oil-water mixtures (Fig. 4, E and F), as captured in movie S1, which highlights the system’s potential for real-time marine monitoring. Under 1.4 μm LED illumination, the array distinguishes oil, water, and air bubbles at the single-pixel spatial scale (Fig. 4G): water strongly absorbs 1.4 μm light and appears darker in grayscale images, oil shows weaker absorption and appears brighter, and bubbles are identified by characteristic scattering and refraction features, enabling precise discrimination of millimetre-scale droplets and thin films. Collectively, these results demonstrate that the SWIR OPD-based sensor array achieves high accuracy, sensitivity, and spatial resolution for oil-leak detection under realistic and complex seawater conditions, highlighting its potential for practical marine environmental monitoring.

#### Identification of textile fabrics

According to official European Union data, approximately 5 million tons of clothing are discarded annually within the European Union, which is equivalent to an average of 12 kg per person. Yet only about 1% is recycled into new garments, while the remainder is incinerated or landfilled, thereby intensifying environmental degradation (*47*). Accurate, rapid, and nondestructive fiber identification is essential for effective waste textile recycling. Traditional classification methods, such as manual sorting, burn tests, microscopic examination, and chemical analysis, face serious drawbacks (Fig. 5A). Manual sorting is labor-intensive and prone to errors. Burn tests and chemical analysis are destructive and may create secondary pollutants. Microscopic analysis is time-consuming and difficult to scale. These limitations highlight the urgent need for innovative classification techniques that provide speed, accuracy, and nondestructive performance.

**Fig. 5.**
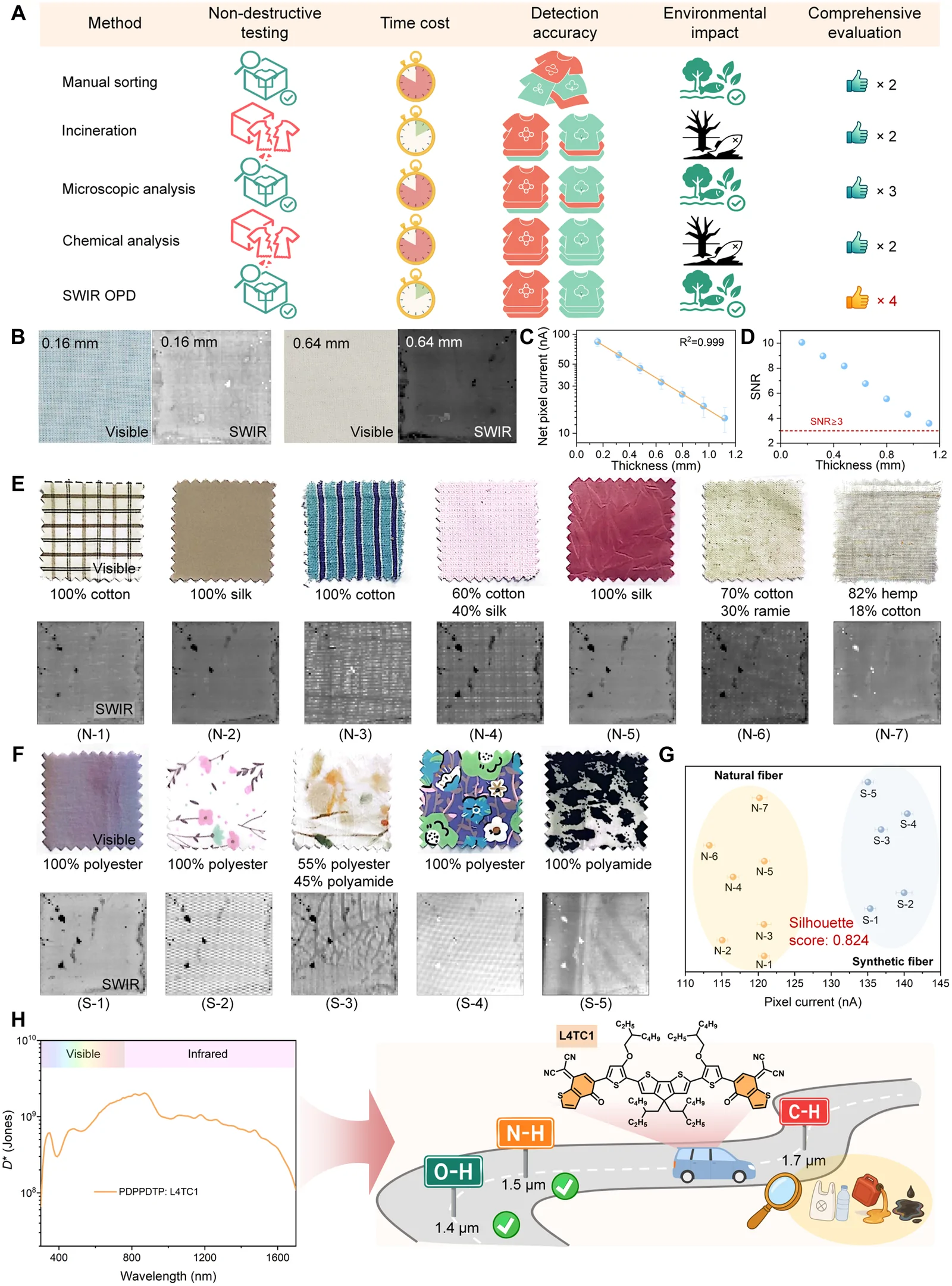
Identification of textile fabrics. (**A**) Comparison of textile fabric identification methods, including manual sorting, incineration, microscopic analysis, chemical analysis, and imaging with the OPD-integrated SWIR sensor array. The comprehensive evaluation (indicated by the number of thumbs-up symbols) for each method is obtained based on a combined assessment of four key evaluation indices relevant to textile fabric identification. (**B**) SWIR imaging of 100% cotton fabrics with thicknesses of 0.16 mm and 0.64 mm (also presented in fig. S42), alongside corresponding photographs of the samples. The fabric samples measure 5 cm by 5 cm, and the imaging area is 3.2 cm by 3.2 cm. (**C**) Net photocurrent as a function of cotton fabric thickness with the corresponding fitted curve. Error bars represent the standard error of the mean. Quantitative analysis is performed using the average net photocurrent values from a 15 by 16 pixel matrix in the central region of the sample image. (**D**) Variation of SNR with fabric thickness for 100% cotton samples. Image sensing of natural fibers (**E**) and synthetic fibers (**F**) under the illumination of 1.4 μm LED without ambient light, and the corresponding photos of fabric swatches. The fabric samples measure 5 cm by 5 cm, and the imaging area is 3.2 cm by 3.2 cm. (**G**) Average pixel currents of natural and synthetic fibers and their clustering analysis. Error bars represent the standard error of the mean. Quantitative analysis is performed using the average photocurrent values from a 15 by 16 pixel matrix in the central region of the sample image. (**H**) The D* curves of SWIR OPDs based on PDPPDTP: L4TC1 under zero bias. The broad response range covers O–H, N–H, and C–H overtones, promising molecular fingerprint detection in various environments.

Natural fibers such as cotton, wool, and silk are rich in O–H or N–H bonds, originating from cellulose in plant-based fibers and peptide linkages in protein-based fibers. In contrast, most synthetic fibers, such as polyester and acrylic, contain few or no O–H or N–H bonds, while polyamide-based fibers such as nylon contain N–H bonds but lack the strong O–H absorption characteristic of natural cellulose (*48*). As we mentioned above, these functional groups exhibit distinct first-overtone absorptions around 1.4 μm in the SWIR, so illumination near this wavelength produces stronger attenuation in fibers rich in cellulose or protein than in polyester or nylon. The resulting intensity contrasts can be quantified with a SWIR OPD-based sensor array to enable rapid, nondestructive classification of natural and synthetic textiles.

Given the variability in textile thickness, it is essential to establish its influence on OPD response. Under 1.4 μm illumination, the net photocurrent (dark-subtracted) was found to decrease exponentially with thickness (Fig. 5, B and C and fig. S42) according to

$$J_{P}\left(t\right)=J_{0}e^{-\mu_{\mathrm{eff}}t}$$ (3)

where *J*_P_(*t*) is the net pixel photocurrent at thickness *t* (mm), *J*_0_ is the extrapolated zero-thickness photocurrent (A), and μ_eff_ is the effective attenuation coefficient (mm^−1^) that reflects absorption and scattering. Fitting yielded μ_eff_ = 1.89 mm^−1^ and *J*_0_ = 113.67 nA. The SNR decreased systematically from ∼10 at 0.16 mm to ∼3.6 at 1.12 mm, and according to the Rose criterion (*49*), which regards SNR ≥ 3 as the minimum threshold for reliable detection (Fig. 5D), signals observed up to 1.12 mm can be assigned to genuine peaks rather than random noise with a confidence above 99.7%, thereby establishing the detection limit beyond 1.12 mm under the present conditions.

All textile samples used for classification were prepared with thicknesses in the 0.3–0.4 mm range, which lies within the high-SNR regime established above and thereby avoids confounding effects from excessive optical attenuation. Consequently, we selected twelve pure natural or synthetic fabrics, dried to minimize moisture interference, and imaged them under 1.4 μm LED illumination using an OPD-based sensor array, converting colored swatches to grayscale according to the measured photocurrent (Fig. 5, E and F). Statistical analysis of identical regions from each grayscale image yielded average pixel currents in the range of 113–141 nA with standard deviations of 0.74–1.11 nA, corresponding to less than 1% of the mean values and reflecting the high precision and reproducibility of the measurements. K-means clustering (K = 2, Euclidean distance) classified the twelve averaged currents into two well-separated groups. Cluster 0 (synthetic fibers) comprised five samples with currents of 135.04–140.44 nA with a mean of 137.53 nA. Cluster 1 (natural fibers) comprised seven samples with currents of 113.29–120.84 nA with a mean of 118.21 nA (Fig. 5G). The inter-cluster difference of approximately 19.3 nA was over 20 times greater than the average measurement uncertainty of 0.84 nA, providing a SNR of 7.3 relative to error bars, ensuring robust, error-resilient classification precision. Cluster validity indices confirmed high reliability, with a silhouette score of 0.824 (ranging from −1 to 1, with unity representing optimal quality), indicating strong intra-cluster cohesion and excellent separation between natural and synthetic fibers, with no negative values suggestive of misclassification. Collectively, these results demonstrate that OPD-based sensor arrays enable precise and reliable molecular-level classification of textiles, achieving rapid, nondestructive discrimination between natural and synthetic fibers and supporting sustainable recycling within a circular economy framework.

These application demonstrations show that SWIR OPD-based sensor arrays, by identifying O–H, and N–H absorptions, enable pollution monitoring as well as waste identification and sorting. Extending device response beyond ∼1.6 μm enables direct detection of C–H overtones characteristic of hydrocarbons and polymer backbones, while enhanced sensitivity near 1.5–1.6 μm facilitates the detection of N–H features and their reliable discrimination from the stronger O–H bands of water. Accordingly, the strong electron-donating unit 2,6-bis(3-alkoxythiophen-2-yl)cyclopentadithiophene (COT) was coupled with the TC1 end group to synthesize L4TC1 (fig. S7) (*15*), which exhibits an ultra-narrow *E*_g_^opt^ of 0.68 eV and an absorption onset beyond 2 μm (fig. S43), surpassing the ∼1.7 μm photoresponse limit of Ge and common InGaAs. Furthermore, the PDPPDTP (*50*): L4TC1-based SWIR OPDs exhibit a spectral response extending to >1.7 μm (detection limit of the instrument) with a peak responsivity of 0.012 A W^−1^ at 1.18 μm as well as an average *i*_n_ of 2.18 × 10^−12^ A Hz^−1/2^ (fig. S44), corresponding to D* values exceeding 10^8^ Jones over the 0.3–1.7 μm range and reaching a maximum of 1.06 × 10^9^ Jones at 1.18 μm (Fig. 5H). The broad response range covers O–H, N–H, and C–H overtones, and thus holds promise for imaging applications like waste plastic sorting and microplastic identification.

## DISCUSSION

In summary, this work couples terminal-guided SWIR molecular engineering with solution-processed active-matrix integration to realize a high-performance OPD-based SWIR imager with photoresponse beyond 1.4 μm, enabling efficient and nondestructive monitoring of environmental pollution and waste. The developed SWIR semiconductors featuring (halogenated) thiophene-fused quinoid terminals possess both ultra-narrow bandgaps and low energetic disorder, enabling the corresponding SWIR OPDs to exhibit a broad spectral response of 0.3–1.6 μm, simultaneously high *R* and D* values beyond 1.4 μm, as well as a large LDR. Finally, the resulting SWIR imager leverages vibrational-overtone molecular signatures to achieve pixel-level detection of simulated seawater oil spills with accurate quantification across the full concentration range and a high SNR of 4.59, as well as reliable differentiation between natural and synthetic fibers with a silhouette score of 0.824 and an SNR of 7.3, underscoring its potential for practical marine monitoring and textile recycling. This work provides a feasible route to low-cost, consumer-grade, high-performance SWIR sensing systems with broadband photoresponse extending beyond 1.4–1.7 μm, supporting progress toward wide-scale smart resource management and sustainable environmental monitoring.

## MATERIALS AND METHODS

### Synthesis of materials

The derivatives TCx-Br (x = 1, 2) and XTC1-Br (X = Cl, Br) of TCx and XTC1 were synthesized from 6,7-dihydrobenzo[b]thiophen-4(5H)-one via distinct multi-step routes. TC1-Br was obtained through bromination, cyclization, aromatization, and Knoevenagel condensation, while TC2-Br involved a Wolff-Kishner reduction and oxidation before the same sequence. XTC1-Br were prepared by halogen substitution, followed by the same synthetic procedures as for TC1-Br. Then, LTCx and LTC1-X were obtained by Stille coupling reaction between the bis-stannylated BTP unit (BTP-2SnMe_3_) and TCx-Br/XTC1-Br, respectively. The detailed synthetic routes are shown in Supplementary.

### Device fabrication and characterization

Indium tin oxide (ITO)-coated glass substrates were sequentially cleaned and treated by UV–ozone (20 min). Two OPD architectures were fabricated.

Inverted architecture (ITO/ZnO/active layer/MoO_3_/Ag). A ∼ 30 nm ZnO electron-transport layer was deposited from a precursor solution by spin-coating (4000 rpm, 30 s) and annealed at 200°C for 30 min. Active layers were processed in nitrogen: for PTB7-Th:L2-eC9/LTC1/LTC2, chlorobenzene solutions (1:1, w/w; 24 mg mL^−1^ total) containing 4 vol% 1-chloronaphthalene (1-CN) were spin-coated at 1400 rpm for 30 s and annealed at 80°C for 5 min; for L4TC1 devices, PDPPDTP:L4TC1 (1:1.2, w/w) was dissolved in chlorobenzene with 0.2 vol% 1-CN (8 mg mL^−1^ total) and spin-coated at 1400 rpm for 30 s. Subsequently, MoO_3_ (∼5 nm) and Ag (∼80 nm) were thermally evaporated under high vacuum (∼1 × 10^−5^ Pa).

Conventional architecture (ITO/PEDOT: PSS/active layer/PDINN/Ag). A ∼ 30 nm PEDOT: PSS layer (Heraeus Clevios P VP AI 4083; 0.45 μm filtered) was spin-coated at 4000 rpm for 30 s and baked at 150°C for 15 min. PTB7-Th: LTC1-X (X = Cl, Br) blends (1:1, w/w) were prepared in chlorobenzene with 3 vol% 1-CN and spin-coated at 1200 rpm for 30 s in a nitrogen glovebox, followed by thermal annealing at 80°C for 5 min to yield ∼130 ± 5 nm active layers. A PDINN layer (1 mg mL^−1^ in methanol) was spin-coated at 4000 rpm for 30 s. Finally, Ag (∼80 nm) was thermally evaporated under high vacuum (∼1 × 10^−5^ Pa).

EQE spectra and *R* were measured using a solar-cell spectral-response system (QE-R3011, Enli Technology Co., Ltd.). The *i*_n_ was recorded using an SR570 current preamplifier connected to an Agilent 35670A FFT analyzer. The reverse bias was applied using the Bias Voltage function of the SR570, and the voltage magnitude was monitored using a multimeter. Current density-voltage characteristics under AM 1.5G (100 mW cm^−2^) were obtained with an AAA-grade solar simulator (XES-70S1, SAN-EI Electric Co., Ltd.), calibrated by a KG5-filtered silicon reference cell (National Institute of Metrology). The active device area for all OPDs was ∼0.04 cm^2^, verified by optical microscopy.

### Active-matrix SWIR imaging

The a-Si TFT array substrate with patterned ITO pixel electrodes (LinkZill Technology Co., Ltd.) underwent sequential cleaning in deionized water, acetone, and isopropanol (10 min each), followed by ultraviolet-ozone treatment for 20 min. As the purchased readout systems are limited to collecting holes, the hole transport layer of OPDs needed to be deposited directly on the patterned ITO pixel electrodes of the TFT array substrate. Therefore, OPDs with the conventional structure of ITO/PEDOT: PSS/PTB7-Th: LTC1-Cl/PDINN/Ag were employed to integrate with TFT array under the same preparation conditions as those described above for the OPD devices. Finally, device encapsulation employed UV-curable adhesive and a piece of glass to ensure environmental stability.

The SWIR imaging system consisted of a light source with four 1.4 μm LEDs (the light intensity varied slightly across different positions of the sample), imaging targets (waste textile samples or oil-water mixtures), the OPD-integrated TFT array, a commercial matrix readout system (Zill Box, LinkZill) with a built-in flip option for output images, and a mobile terminal for data visualization. Electrical interfacing between the TFT array and readout circuitry was achieved through flexible printed cables. During operation, row-wise scanning applied a 10 V gate voltage for pixel activation. Pixel currents were digitized through 8-bit analog-to-digital conversion and transmitted row-sequentially to the mobile interface in both raw data and reconstructed image modes via wireless protocols. The observed slight inhomogeneity of grayscale across the array mainly stemmed from subtle differences in the light intensity received by the pixel points at different positions and spatial variations in the thickness of the spin-coated active layer. A small fraction of pixels exhibited malfunction (“dead pixels”) in the acquired images, primarily located near the edges of the array. These dead pixels arose from contact imperfections during the wiring/interconnection process, rather than from intrinsic defects of the photoactive material, and they were excluded from analysis. For quantitative evaluation of the pixel current in the imaging results, the mean value within each region was used as the representative signal, while the standard deviation was taken as the uncertainty.

### Oil spill detection

Commercial No. 0 diesel obtained from a local gas station and seawater collected from the Bohai Sea were used to simulate marine oil-spill conditions. The oil fraction ϕ_oil_ was defined on a volumetric basis according to

$$\phi_{\text{oil}}=\frac{V_{\text{oil}}}{V_{\text{oil}}+V_{\text{water}}}$$ (4)

where *V*_oil_ and *V*_water_ are the volumes of diesel oil and seawater, respectively. The total solution volume was fixed at 10 mL. To emulate emulsification in real seawater, 100 ppm sodium dodecyl sulfate (SDS) was added to the oil–water mixtures, followed by mechanical stirring at 1000 rpm for 1 h to produce stable emulsions before measurement. An active-matrix sensor array based on SWIR OPDs was used to record the photocurrent under 1.4 μm LED illumination. The net photocurrent was defined as

$$J_{P}=J_{\text{on}}-J_{\text{dark}}$$ (5)

where *J*_on_ and *J*_dark_ are the currents measured under illumination and in the dark, respectively. The photocurrent–concentration relationship was modeled using Eq. 2, and linear fitting was performed by plotting ln*J*_P_ versus ϕ_oil_. Baseline deviation was quantified by comparing the extrapolated zero-content photocurrent *J*_0_ with the measured blank *J*_blank_ according to

$$\text{Deviation}\left(\%\right)=\frac{|J_{0}-J_{\text{blank}}|}{J_{\text{blank}}}\times 100\%$$ (6)

The SNR was defined relative to the water baseline as

$$\text{SNR}=\frac{J_{P}^{\text{oil}}-J_{P}^{\text{water}}}{\sigma \left(J_{P}^{\text{water}}\right)}$$ (7)

where $J_{P}^{\text{oil}}$ and $J_{P}^{\text{water}}$ denote the mean net photocurrents of oil and water, respectively, and $\sigma \left(J_{P}^{\text{water}}\right)$ is the standard deviation of the water photocurrent. Reliable detection was judged according to the Rose criterion (SNR ≥ 3). Droplet-size tests were performed with diameters in the millimeter range to evaluate the spatial resolution limit. Note: In the experiments for detecting oil droplets of different sizes, the petri dish was suspended above the detector using an iron ring wrapped with aluminum foil as a supporting frame, as shown in Fig. 4D. This setup avoided direct mechanical pressure on the detector during long-term measurements, thereby ensuring device stability.

### Clustering analysis

Average pixel currents obtained from OPD-based images were clustered using the K-means algorithm (k = 2, Euclidean distance). Each data point was assigned to the nearest cluster centroid, and the classification threshold was defined as the midpoint between the two centroids. For this dataset, the centroids were 1.1821 × 10^−7^ A and 1.3753 × 10^−7^ A, giving a threshold of

$$T=\frac{\mu_{L+}\mu_{H}}{2}=1.2787\times 10^{-7}\;A\left(\approx 127.9\;\text{nA}\right)$$ (8)

The quality of clustering was evaluated using the silhouette coefficient, calculated as

$$s=\frac{b-a}{\text{max}\left(a,b\right)}$$ (9)

where a is the mean intra-cluster distance of a sample and *b* is the mean nearest-cluster distance. The silhouette coefficient ranges from −1 to 1. Values close to 1 indicate well-separated and compact clusters. Values near 0 indicate overlapping clusters or little structure. To assess the robustness of the classification against measurement uncertainty, a SNR was introduced and defined for each sample as

$$r_{i}=\frac{|x_{i}-T|}{e_{i}}$$ (10)

where *x_i_* is the sample value, *e_i_* is the associated error bar (standard deviation), and *T* is the classification threshold. The SNR across all samples was 7.3, quantifying the worst-case margin of classification confidence.

## Supplementary Materials

### The PDF file includes:

Supplementary Text Figs. S1 to S44 Tables S1 to S4 Legend for movie S1 Notes S1 to S4 References

## References

[1] Q. Duan, J. Li, Classification of common household plastic wastes combining multiple methods based on near-infrared spectroscopy. ACS EST Engg. 1, 1065–1073 (2021).

[2] L. G. Weyer, Near-infrared spectroscopy of organic substances. Appl. Spectrosc. Rev. 21, 1–43 (1985).

[3] THE 17 GOALS | Sustainable development. https://sdgs.un.org/goals.

[4] J.-P. Lange, Managing plastic waste─sorting, recycling, disposal, and product redesign. ACS Sustain. Chem. Eng. 9, 15722–15738 (2021).

[5] J. Michel, J. Liu, L. C. Kimerling, High-performance Ge-on-Si photodetectors. Nat. Photon. 4, 527–534 (2010).

[6] F. Teng, K. Hu, W. Ouyang, X. Fang, Photoelectric detectors based on inorganic p-type semiconductor materials. Adv. Mater. 30, e1706262 (2018). 10.1002/adma.201706262 29888448

[7] Y. Chen, Y. Zheng, J. Wang, X. Zhao, G. Liu, Y. Lin, Y. Yang, L. Wang, Z. Tang, Y. Wang, Y. Fang, W. Zhang, X. Zhu, Ultranarrow-bandgap small-molecule acceptor enables sensitive SWIR detection and dynamic upconversion imaging. Sci. Adv. 10, eadm9631 (2024). 10.1126/sciadv.adm9631 PMC11152131 38838154

[8] C. Wang, X. Zhang, W. Hu, Organic photodiodes and phototransistors toward infrared detection: Materials, devices, and applications. Chem. Soc. Rev. 49, 653–670 (2020). 10.1039/c9cs00431a 31829375

[9] C. Fuentes-Hernandez, W.-F. Chou, T. M. Khan, L. Diniz, J. Lukens, F. A. Larrain, V. A. Rodriguez-Toro, B. Kippelen, Large-area low-noise flexible organic photodiodes for detecting faint visible light. Science 370, 698–701 (2020). 10.1126/science.aba2624 33154137

[10] T. Kamijo, A. J. J. M. van Breemen, X. Ma, S. Shanmugam, T. Bel, G. de Haas, B. Peeters, R. Petre, D. Tordera, R. Verbeek, H. B. Akkerman, L. M. Hagelsieb, F. de Roose, I. Lieberman, F. Yamaguchi, R. A. J. Janssen, E. A. Meulenkamp, A. J. Kronemeijer, G. H. Gelinck, A touchless user interface based on a near-infrared-sensitive transparent optical imager. Nat. Electron. 6, 451–461 (2023).

[11] Y. Ma, X. Xu, T. Li, Z. Wang, N. Li, X. Zhao, W. Wei, X. Zhan, L. Shen, Amplified narrowband perovskite photodetectors enabled by independent multiplication layers for anti-interference light detection. Sci. Adv. 11, eadq1127 (2025). 10.1126/sciadv.adq1127 PMC12124361 40446029

[12] C. Li, H. Wang, F. Wang, T. Li, M. Xu, H. Wang, Z. Wang, X. Zhan, W. Hu, L. Shen, Ultrafast and broadband photodetectors based on a perovskite/organic bulk heterojunction for large-dynamic-range imaging. Light Sci. Appl. 9, 31 (2020). 10.1038/s41377-020-0264-5 PMC7054320 32194945

[13] C. Yan, S. Barlow, Z. Wang, H. Yan, A. K.-Y. Jen, S. R. Marder, X. Zhan, Non-fullerene acceptors for organic solar cells. Nat. Rev. Mater. 3, 18003 (2018).

[14] D. Nodari, Z. Qiao, F. Furlan, O. J. Sandberg, K. Vandewal, N. Gasparini, Towards high and reliable specific detectivity in visible and infrared perovskite and organic photodiodes. Nat. Rev. Mater. 10, 842–856 (2025).

[15] T. Li, G. Hu, L. Tao, J. Jiang, J. Xin, Y. Li, W. Ma, L. Shen, Y. Fang, Y. Lin, Sensitive photodetection below silicon bandgap using quinoid-capped organic semiconductors. Sci. Adv. 9, eadf6152 (2023). 10.1126/sciadv.adf6152 PMC10058242 36989368

[16] Y. Zhang, J. Chen, J. Yang, M. Fu, Y. Cao, M. Dong, J. Yu, S. Dong, X. Yang, L. Shao, Z. Hu, H. Cai, C. Liu, F. Huang, Sensitive SWIR organic photodetectors with spectral response reaching 1.5 μm. Adv. Mater. 36, e2406950 (2024). 10.1002/adma.202406950 39152933

[17] J. Wang, Y. Xie, K. Chen, H. Wu, J. M. Hodgkiss, X. Zhan, Physical insights into non-fullerene organic photovoltaics. Nat. Rev. Phys. 6, 365–381 (2024).

[18] O. J. Sandberg, C. Kaiser, S. Zeiske, N. Zarrabi, S. Gielen, W. Maes, K. Vandewal, P. Meredith, A. Armin, Mid-gap trap state-mediated dark current in organic photodiodes. Nat. Photonics 17, 368–374 (2023).

[19] P. Jacoutot, A. D. Scaccabarozzi, D. Nodari, J. Panidi, Z. Qiao, A. Schiza, A. D. Nega, A. Dimitrakopoulou-Strauss, V. G. Gregoriou, M. Heeney, C. L. Chochos, A. A. Bakulin, N. Gasparini, Enhanced sub-1 eV detection in organic photodetectors through tuning polymer energetics and microstructure. Sci. Adv. 9, eadh2694 (2023). 10.1126/sciadv.adh2694 PMC10246890 37285428

[20] J. Xu, Y. Zhang, J. Liu, L. Wang, NIR-II absorbing monodispersed oligomers based on N-B←N unit. Angew. Chem. Int. Ed. Engl. 62, e202310838 (2023). 10.1002/anie.202310838 37635075

[21] B. Yin, X. Zhou, Y. Li, G. Hu, W. Wei, M. Yang, S. Jeong, W. Deng, B. Wu, Y. Cao, B. Huang, L. Pan, X. Yang, Z. Fu, Y. Fang, L. Shen, C. Yang, H. Wu, L. Lan, F. Huang, Y. Cao, C. Duan, Sensitive organic photodetectors with spectral response up to 1.3 μm using a quinoidal molecular semiconductor. Adv. Mater. 36, e2310811 (2024). 10.1002/adma.202310811 38358297

[22] W. Liu, W. Deng, W. Wang, H. Wu, C. Gao, Y. Xie, J. Zhao, X. Dong, Z. Zhao, Z. Zheng, Y. Chi, L. Duan, X. Zhan, Y. Zou, H. Wu, J. Peng, Y. Cao, Ultrahigh-radiance near-infrared organic light-emitting diodes. Nat. Photonics 19, 650–657 (2025).

[23] W. Guo, Y. Duan, W. Liu, X. Zhang, J. Gao, Z. You, Y. Jiang, Z. Ma, X. Zhan, Y. Liu, Ultranarrow bandgap electron acceptor with low reorganization energy and refined crystallization enables efficient self-powered photodetectors with spectral response over 1100 nm. CCS Chem. 7, 1731–1744 (2024).

[24] R. R. Birge, G. A. Schick, D. F. Bocian, Calculation of molecular polarizabilities using a semiclassical slater-type orbital-point dipole interaction (STOPDI) model. J. Chem. Phys. 79, 2256–2264 (1983).

[25] M. N. Bellido, On the polarizability of the sulfur atom. Chem. Phys. Lett. 122, 562–566 (1985).

[26] H. Yao, L. Ye, J. Hou, B. Jang, G. Han, Y. Cui, G. M. Su, C. Wang, B. Gao, R. Yu, H. Zhang, Y. Yi, H. Y. Woo, H. Ade, J. Hou, Achieving highly efficient nonfullerene organic solar cells with improved intermolecular interaction and open-circuit voltage. Adv. Mater. 29, 1700254 (2017). 10.1002/adma.201700254 28370383

[27] Y. Cui, P. Zhu, X. Shi, X. Liao, Y. Chen, Theoretical study of excited state charge transfer characteristics based on A–D–A and A–DA′D–A type nonfullerene acceptors. J. Phys. Chem. C 125, 10250–10259 (2021).

[28] S. Kuila, H. Miranda-Salinas, J. Eng, C. Li, M. R. Bryce, T. J. Penfold, A. P. Monkman, Rigid and planar π-conjugated molecules leading to long-lived intramolecular charge-transfer states exhibiting thermally activated delayed fluorescence. Nat. Commun. 15, 9611 (2024). 10.1038/s41467-024-53740-1 PMC11544105 39511188

[29] S. Simon, M. Duran, J. J. Dannenberg, How does basis set superposition error change the potential surfaces for hydrogen-bonded dimers? J. Chem. Phys. 105, 11024–11031 (1996).

[30] S. Grimme, J. Antony, S. Ehrlich, H. Krieg, A consistent and accurate ab initio parametrization of density functional dispersion correction (DFT-D) for the 94 elements H-Pu. J. Chem. Phys. 132, 154104 (2010). 10.1063/1.3382344 20423165

[31] G. Zhang, X.-K. Chen, J. Xiao, P. C. Y. Chow, M. Ren, G. Kupgan, X. Jiao, C. C. S. Chan, X. Du, R. Xia, Z. Chen, J. Yuan, Y. Zhang, S. Zhang, Y. Liu, Y. Zou, H. Yan, K. S. Wong, V. Coropceanu, N. Li, C. J. Brabec, J.-L. Bredas, H.-L. Yip, Y. Cao, Delocalization of exciton and electron wavefunction in non-fullerene acceptor molecules enables efficient organic solar cells. Nat. Commun. 11, 3943 (2020). 10.1038/s41467-020-17867-1 PMC7414148 32770068

[32] C. W. Bunn, H. P. Klug, Chemical crystallography. J. Phys. Chem. 50, 490–491 (1946).

[33] C. Li, J. Zhou, J. Song, J. Xu, H. Zhang, X. Zhang, J. Guo, L. Zhu, D. Wei, G. Han, J. Min, Y. Zhang, Z. Xie, Y. Yi, H. Yan, F. Gao, F. Liu, Y. Sun, Non-fullerene acceptors with branched side chains and improved molecular packing to exceed 18% efficiency in organic solar cells. Nat. Energy 6, 605–613 (2021).

[34] Z. Zhang, Y. Li, G. Cai, Y. Zhang, X. Lu, Y. Lin, Selenium heterocyclic electron acceptor with small urbach energy for as-cast high-performance organic solar cells. J. Am. Chem. Soc. 142, 18741–18745 (2020). 10.1021/jacs.0c08557 33085460

[35] S. Karuthedath, J. Gorenflot, Y. Firdaus, N. Chaturvedi, C. S. P. De Castro, G. T. Harrison, J. I. Khan, A. Markina, A. H. Balawi, T. A. D. Peña, W. Liu, R.-Z. Liang, A. Sharma, S. H. K. Paleti, W. Zhang, Y. Lin, E. Alarousu, S. Lopatin, D. H. Anjum, P. M. Beaujuge, S. De Wolf, I. McCulloch, T. D. Anthopoulos, D. Baran, D. Andrienko, F. Laquai, Intrinsic efficiency limits in low-bandgap non-fullerene acceptor organic solar cells. Nat. Mater. 20, 378–384 (2021). 10.1038/s41563-020-00835-x 33106652

[36] G. Yu, J. Gao, J. C. Hummelen, F. Wudl, A. J. Heeger, Polymer photovoltaic cells: Enhanced efficiencies via a network of internal donor-acceptor heterojunctions. Science 270, 1789–1791 (1995).

[37] S. Gielen, C. Kaiser, F. Verstraeten, J. Kublitski, J. Benduhn, D. Spoltore, P. Verstappen, W. Maes, P. Meredith, A. Armin, K. Vandewal, Intrinsic detectivity limits of organic near-infrared photodetectors. Adv. Mater. 32, e2003818 (2020). 10.1002/adma.202003818 33078513

[38] Z. Wu, N. Li, N. Eedugurala, J. D. Azoulay, D.-S. Leem, T. N. Ng, Noise and detectivity limits in organic shortwave infrared photodiodes with low disorder. npj Flex. Electron. 4, 6 (2020).

[39] X. Li, Q. Zhang, J. Yu, Y. Xu, R. Zhang, C. Wang, H. Zhang, S. Fabiano, X. Liu, J. Hou, F. Gao, M. Fahlman, Mapping the energy level alignment at donor/acceptor interfaces in non-fullerene organic solar cells. Nat. Commun. 13, 2046 (2022). 10.1038/s41467-022-29702-w PMC9018783 35440117

[40] J. B. Barton, R. F. Cannata, S. M. Petronio, InGaAs NIR focal plane arrays for imaging and DWDM applications. Proc. SPIE 4721, 37–47 (2002).

[41] T. Li, Y. Qu, Y. Peng, H. Liu, Y. Yang, J. Zhang, H. Hou, L. Li, Q. Lin, X. Lu, Y. Yang, Y. Fang, Y. Lin, Organic semiconductors with halogenated quinoid terminals for sensitive short-wave infrared detection and imaging. Adv. Mater. 38, e22336 (2026). 10.1002/adma.202522336 41580947

[42] D. Geng, K. Wang, L. Li, K. Myny, A. Nathan, J. Jang, Y. Kuo, M. Liu, Thin-film transistors for large-area electronics. Nat. Electron. 6, 963–972 (2023).

[43] S. E. Chang, Potential impacts of an impending oil spill. Nat. Sustain. 4, 1023–1024 (2021).

[44] M. Xie, Y. Li, Z. Zhang, Q. Fu, H. Jiang, Remote sensing of the oil spills caused by ships: A review. Mar. Pollut. Bull. 214, 117754 (2025). 10.1016/j.marpolbul.2025.117754 40037103

[45] H. Pan, K. Tang, J. Zhuo, Y. Lu, J. Chen, Z. Lv, Underwater acoustic technology-based monitoring of oil spill: A review. J. Mar. Sci. Eng. 11, 870 (2023).

[46] S. Y. Venyaminov, F. G. Prendergast, Water (H_2_O and D_2_O) molar absorptivity in the 1000–4000 cm^–1^ range and quantitative infrared spectroscopy of aqueous solutions. Anal. Biochem. 248, 234–245 (1997). 10.1006/abio.1997.2136 9177749

[47] Textiles strategy-european commission (An official website of the European Union, 2025); https://environment.ec.europa.eu/strategy/textiles-strategy_en.

[48] J.-R. Riba, R. Cantero, T. Canals, R. Puig, Circular economy of post-consumer textile waste: Classification through infrared spectroscopy. J. Clean. Prod. 272, 123011 (2020).

[49] A. Rose, The sensitivity performance of the human eye on an absolute scale. J. Opt. Soc. Am. 38, 196–208 (1948). 10.1364/josa.38.000196 18901781

[50] K. H. Hendriks, W. Li, M. M. Wienk, R. A. J. Janssen, Small-bandgap semiconducting polymers with high near-infrared photoresponse. J. Am. Chem. Soc. 136, 12130–12136 (2014). 10.1021/ja506265h 25101518

[51] B. L. Chenard, J. R. McConnell, J. S. Swenton, An efficient route to benzo[b]thiophenequinones, quinone bisketals, and quinone monoketals via anodic oxidation of methoxylated benzo[b]thiophenes. Studies directed at the synthesis of functionalized benzo[b]thiophene-4,7-quinones. J. Org. Chem. 48, 4312–4317 (1983).

[52] Z. Jiang, N. Liu, G. Dong, Y. Jiang, Y. Liu, X. He, Y. Huang, S. He, W. Chen, Z. Li, J. Yao, Z. Miao, W. Zhang, C. Sheng, Scaffold hopping of sampangine: Discovery of potent antifungal lead compound against Aspergillus fumigatus and Cryptococcus neoformans. Bioorg. Med. Chem. Lett. 24, 4090–4094 (2014). 10.1016/j.bmcl.2014.07.064 25115626

[53] Z. Zhang, Y. Yang, T. Li, H. Hou, Y. Geng, Q. Lin, Y. Lin, Deuterating narrow-bandgap organic semiconductors for sensitive short-wave infrared photodetection. Angew. Chem. Int. Ed. Engl. 64, e202507637 (2025). 10.1002/anie.202507637 40392543

[54] M. J. Frisch, G. W. Trucks, H. B. Schlegel, G. E. Scuseria, M. A. Robb, J. R. Cheeseman, G. Scalmani, V. Barone, G. A. Petersson, H. Nakatsuji, X. Li, M. Caricato, A. V. Marenich, J. Bloino, B. G. Janesko, R. Gomperts, B. Mennucci, H. P. Hratchian, J. V. Ortiz, A. F. Izmaylov, J. L. Sonnenberg, D. Williams-Young, F. Ding, F. Lipparini, F. Egidi, J. Goings, B. Peng, A. Petrone, T. Henderson, D. Ranasinghe, V. G. Zakrzewski, J. Gao, N. Rega, G. Zheng, W. Liang, M. Hada, M. Ehara, K. Toyota, R. Fukuda, J. Hasegawa, M. Ishida, T. Nakajima, Y. Honda, O. Kitao, H. Nakai, T. Vreven, K. Throssell, J. A. Montgomery Jr., J. E. Peralta, F. Ogliaro, M. J. Bearpark, J. J. Heyd, E. N. Brothers, K. N. Kudin, V. N. Staroverov, T. A. Keith, R. Kobayashi, J. Normand, K. Raghavachari, A. P. Rendell, J. C. Burant, S. S. Iyengar, J. Tomasi, M. Cossi, J. M. Millam, M. Klene, C. Adamo, R. Cammi, J. W. Ochterski, R. L. Martin, K. Morokuma, O. Farkas, J. B. Foresman, D. J. Fox, Gaussian 16, Revision A.03, Gaussian, Inc., Wallingford CT, (2016).

[55] J. B. Johnson, Thermal agitation of electricity in conductors. Phys. Rev. 32, 97–109 (1928).

[56] G. G. Malliaras, J. R. Salem, P. J. Brock, C. Scott, Electrical characteristics and efficiency of single-layer organic light-emitting diodes. Phys. Rev. B 58, R13411–R13414 (1998).

[57] R. A. Street, Y. Yang, B. C. Thompson, I. McCulloch, Capacitance spectroscopy of light induced trap states in organic solar cells. J. Phys. Chem. C 120, 22169–22178 (2016).

[58] Y. Fang, A. Armin, P. Meredith, J. Huang, Accurate characterization of next-generation thin-film photodetectors. Nat. Photon. 13, 1–4 (2019).

[59] V. Pecunia, T. D. Anthopoulos, A. Armin, B. Bouthinon, M. Caironi, A. Castellanos-Gomez, Y. Chen, K. Cho, C. Clegg, X. Fang, P. Fendel, B. Fowler, G. Gelinck, H. Gottlob, P. Guyot-Sionnest, R. Hannebauer, G. Hernandez-Sosa, M. C. Hersam, L. Hirsch, J. C. Ho, F. H. Isikgor, J. Joimel, H. J. Kim, G. Konstantatos, J. Labram, M. C. Lemme, K. Leo, E. Lhuillier, E. Lidorikis, M. A. Loi, P. E. Malinowski, P. Merken, T. Mueller, B. Nasrollahi, D. Natali, T. N. Ng, T.-Q. Nguyen, S. K. Park, L.-M. Peng, P. Samorì, E. H. Sargent, L. Shen, S. Shishido, I. Shorubalko, P. Sonar, S. D. Stranks, S. F. Tedde, K. Vandewal, M. Verhaegen, S. Walia, F. Yan, T. Yokota, F. Zhang, Guidelines for accurate evaluation of photodetectors based on emerging semiconductor technologies. Nat. Photon. 19, 1178–1188 (2025).

[60] I. Park, C. Kim, R. Kim, N. Li, J. Lee, O. K. Kwon, B. Choi, T. N. Ng, D.-S. Leem, High performance shortwave infrared organic photodetectors adopting thiadiazole quinoxaline-based copolymers. Adv. Opt. Mater. 10, 2200747 (2022).

[61] X. Yu, H. Lin, Z. He, X. Du, Z. Chen, G. Yang, C. Zheng, S. Tao, Efficient near-infrared organic photodetectors with spectral response up to 1600 nm for accurate alcohol concentration detection. ACS Appl. Mater. Interfaces 15, 16918–16929 (2023). 10.1021/acsami.2c22724 36947683

[62] H. Lu, W. Liu, G. Ran, Z. Liang, H. Li, N. Wei, H. Wu, Z. Ma, Y. Liu, W. Zhang, X. Xu, Z. Bo, High-pressure fabrication of binary organic solar cells with high molecular weight D18 yields record 19.65 % efficiency. Angew. Chem. Int. Ed. Engl. 62, e202314420 (2023). 10.1002/anie.202314420 37881111

[63] L. J. A. Koster, V. D. Mihailetchi, R. Ramaker, P. W. M. Blom, Light intensity dependence of open-circuit voltage of polymer:Fullerene solar cells. Appl. Phys. Lett. 86, 123509 (2005).

[64] S. R. Cowan, A. Roy, A. J. Heeger, Recombination in polymer-fullerene bulk heterojunction solar cells. Phys. Rev. B 82, 245207 (2010).

[65] S. Wheeler, F. Deledalle, N. Tokmoldin, T. Kirchartz, J. Nelson, J. R. Durrant, Influence of surface recombination on charge-carrier kinetics in organic bulk heterojunction solar cells with nickel oxide interlayers. Phys. Rev. Appl. 4, 024020 (2015).

