# Supplementary material for: Solution-processed organic infrared photodetectors operating beyond 1.4 μm for sensitive environmental monitoring
Source: Sci Adv. 2026 Oct 7;12(41):eaeg4558. doi: 10.1126/sciadv.aeg4558 (PMC13644758; doi:10.1126/sciadv.aeg4558)
Supplement: Supplementary file 1 [file sciadv.aeg4558_sm.pdf]

Science Advances
AAAS

Supplementary Materials for

Solution-processed organic infrared photodetectors operating beyond 1.4 μm for sensitive environmental monitoring

Yifei Geng *et al.*

Corresponding author: Yuze Lin, linyz@iccas.ac.cn

Sci. Adv. **12**, eaeg4558 (2026)
DOI: 10.1126/sciadv.aeg4558

The PDF file includes:

Supplementary Text
Figs. S1 to S44
Tables S1 to S4
Legend for movie S1
Notes S1 to S4
References

Other Supplementary Material for this manuscript includes the following:

Movie S1

Supplementary Text

Materials

Unless stated otherwise, all chemical reagents and solvents were commercially acquired and used without additional purification. PTB7-Th was purchased from 1-Materials Inc. PDPPDTP, 5-bromobenzo[b]thiophene-4,7-dione, 6-bromobenzo[b]thiophene-4,7-dione, BTP-2SnMe$_3$, COT-2SnMe$_3$, and L2-eC9 were synthesized according to the literature procedure (*15*, *50–53*).

Synthesis

TC1-Br. To a solution of 5-bromobenzo[b]thiophene-4,7-dione (729 mg, 3.0 mmol) and malononitrile (594.5 mg, 9.0 mmol) in anhydrous dichloromethane (30 mL), 1 M titanium tetrachloride in anhydrous dichloromethane (9.0 mL, 9.0 mmol) was added dropwise under nitrogen at 0 °C. After the mixture was stirred at 0 °C for 0.5 h, anhydrous pyridine (711.9 mg, 9.0 mmol) in anhydrous dichloromethane (2 mL) was added dropwise under nitrogen at 0 °C. The mixture was stirred at room temperature for 2 h. Water (30 mL) was added, and the mixture was extracted with dichloromethane (2 × 30 mL). The organic phase was dried over anhydrous MgSO$_4$ and filtered. After removing the solvent from the filtrate, the residue was purified by column chromatography on silica gel using petroleum ether/dichloromethane (1: 1, *v*/*v*) as the eluent yielding an orange solid (260 mg, 30%). ^{1}H NMR (300 MHz, Chloroform-*d*) δ 8.15 (s, 1H), 7.79 (q, *J* = 5.2 Hz, 2H). ^{13}C NMR (75 MHz, Chloroform-*d*) δ 172.90, 148.40, 139.65, 137.34, 135.98, 134.73, 134.26, 127.82, 113.13, 111.87, 83.03. MS (EI): *m/z* 289.9 (M$^+$).

TC2-Br. The synthetic routes are similar to that of TC1-Br, using 6-bromobenzo[b]thiophene-4,7-dione as the starting material (orange solid, 28%). ^{1}H NMR (300 MHz, Chloroform-*d*) δ 8.33 (d, *J* = 5.3 Hz, 1H), 8.13 (s, 1H), 7.88 (d, *J* = 5.4 Hz, 1H). ^{13}C NMR (100 MHz, Chloroform-d) δ 170.87, 147.65, 140.37, 136.89, 135.56, 134.61, 134.18, 128.43, 113.41, 112.03, 84.31. MS (EI): *m/z* 289.9 (M$^+$).

ClTC1-Br: The 5,5-dibromo-6,7-dihydrobenzo[b]thiophen-4(5H)-one was chlorinated with N-Chlorosuccinimide (NCS) to give 5,5-dibromo-2-chloro-6,7-dihydrobenzo[b]thiophen-4(5H)-one. The subsequent synthetic procedures followed those described for TC1-Br (red solid, 35%). ^{1}H NMR (400 MHz, Chloroform-*d*) δ 8.12 (s, 1H), 7.59 (s, 1H). ^{13}C NMR (100 MHz, Chloroform-*d*) δ 171.95, 147.45, 141.38, 139.23, 135.99, 135.19, 134.03, 126.43, 113.08, 111.63, 83.13. MS (EI): *m/z* 325.9 (M$^+$).

BrTC1-Br: The 5,5-Dibromo-6,7-dihydrobenzo[b]thiophen-4(5H)-one was brominated with N-Bromosuccinimide (NBS) to give 2,5,5-tribromo-6,7-dihydrobenzo[b]thiophen-4(5H)-one. The subsequent synthetic procedures followed those described for TC1-Br (red solid, 33%). ^{1}H NMR (400 MHz, Chloroform-*d*) δ 8.11 (s, 1H), 7.74 (s, 1H). ^{13}C NMR (100 MHz, Chloroform-*d*) δ 171.84, 147.40, 139.82, 138.01, 135.93, 134.02, 130.15, 124.72, 113.16, 111.69, 83.27. MS (EI): *m/z* 370.1 (M$^+$).

LTC1. To a three-necked round bottom flask were added BTP-2SnMe$_3$ (155 mg, 0.1 mmol), TC1-Br (72.8 mg, 0.25 mmol), and toluene (20 mL). The mixture was deoxygenated with nitrogen for 15

min, and $Pd(PPh_3)_4$ (5.7 mg, 0.005 mmol) was then added under nitrogen. The mixture was refluxed for 12 h and then cooled down to room temperature. Water (30 mL) was added, and the mixture was extracted with dichloromethane (2 × 30 mL). The organic phase was dried over anhydrous $MgSO_4$ and filtered. After removing the solvent from the filtrate, the residue was purified by column chromatography on silica gel using petroleum ether/dichloromethane (1:1) as the eluent yielding a brown-black solid (60 mg, 41%). ^{1}H NMR (300 MHz, Chloroform-*d*) δ 8.01-7.68 (m, 6H), 4.65 (d, *J* = 7.8 Hz, 4H), 3.12 (d, *J* = 25.0 Hz, 4H), 2.00 (m, 6H), 1.48-1.18 (m, 26H), 1.16-0.74 (m, 36H), 0.72-0.56 (m, 12H). ^{13}C NMR (100 MHz, Chloroform-*d*) δ 178.74, 149.80, 149.66, 142.47, 142.41, 140.24, 139.74, 139.15, 135.22, 134.02, 132.50, 131.55, 130.31, 125.42, 122.38, 120.84, 114.68, 113.13, 111.89, 72.10, 49.76, 37.65, 31.81, 31.79, 31.23, 31.15, 29.61, 29.44, 29.41, 29.28, 29.21, 29.19, 29.13, 28.49, 26.71, 22.99, 22.69, 14.07, 14.03. MS (MALDI-TOF): *m/z* 1445.8 (M^+).

The synthetic routes of LTC2 (brown black solid, 35%), LTC1-Cl (brown black solid, 13%), LTC1-Cl (brown black solid, 15%), L4TC1 (brown black solid, 46%) are similar to that of LTC1.

LTC2. ^{1}H NMR (300 MHz, Chloroform-*d*) δ 8.38 (d, *J* = 5.3 Hz, 2H), 7.91 (d, *J* = 5.3 Hz, 2H), 7.82 (s, 2H), 4.65 (d, J = 7.9 Hz, 4H), 3.09 (t, J = 8.2 Hz, 4H), 2.14-1.95 (m, 6H), 1.36-1.23 (m, 26H), 1.04-0.84 (m, 36H), 0.65 (m, 12H). ^{13}C NMR (100 MHz, Chloroform-*d*) δ 178.51, 148.68, 147.78, 145.86, 141.94, 141.32, 137.66, 137.60, 136.67, 135.08, 132.98, 130.12, 129.20, 127.94, 126.43, 114.79, 113.36, 112.79, 82.38, 55.64, 39.02, 32.07, 31.71, 31.02, 30.59, 30.48, 30.35, 30.15, 29.74, 29.59, 29.53, 29.50, 29.35, 28.14, 28.00, 25.49, 25.32, 22.94, 22.91, 22.86, 22.63, 22.62, 14.29, 14.14, 13.91, 13.87. MS (MALDI-TOF): *m/z* 1447.8 (M^+).

LTC1-Cl. ^{1}H NMR (400 MHz, Chloroform-*d*) δ 7.79 (s, 2H), 7.61 (s, 2H), 4.64 (d, *J* = 8.0 Hz, 4H), 3.07 (t, *J* = 8.2 Hz, 4H), 2.09-1.98 (m, 6H), 1.47-1.21 (m, 26H), 0.93 (m, 36H), 0.65 (m, 12H). ^{13}C NMR (100 MHz, Chloroform-*d*) δ 178.76, 147.62, 145.87, 141.47, 139.81, 139.29, 137.49, 136.23, 136.06, 132.93, 130.22, 128.96, 126.97, 125.90, 114.33, 112.80, 112.67, 79.68, 55.43, 53.44, 41.36, 38.86, 36.09, 34.68, 33.73, 31.92, 31.61, 31.57, 30.90, 30.29, 29.99, 29.72, 29.58, 29.44, 29.36, 29.20, 29.08, 28.02, 27.69, 25.29, 22.81, 22.77, 22.72, 22.67, 22.63, 22.47, 20.46, 19.45, 18.77, 14.34, 14.15, 14.01, 13.79, 13.75, 11.45. MS (MALDI-TOF): *m/z* 1518.8 (M^+).

LTC1-Br. ^{1}H NMR (400 MHz, Chloroform-*d*) δ 7.72 (s, 2H), 7.69 (s, 2H), 4.58 (s, 4H), 3.00 (t, J = 8.3 Hz, 4H), 1.91 (s, 6H), 1.30-1.12 (m, 26H), 0.86-0.74 (m, 36H), 0.58 (m, 12H). ^{13}C NMR (100 MHz, Chloroform-*d*) δ 178.67, 147.59, 145.88, 141.48, 139.93, 138.84, 137.50, 136.18, 132.94, 130.24, 129.66, 128.96, 126.90, 126.56, 123.04, 114.40, 112.86, 112.68, 79.78, 77.35, 77.03, 76.71, 55.44, 38.87, 31.92, 31.56, 30.91, 30.42, 30.29, 30.14, 29.98, 29.58, 29.44, 29.36, 29.18, 28.02, 22.81, 22.77, 22.71, 22.48, 22.46, 14.16, 14.01, 13.80, 13.76. MS (MALDI-TOF): *m/z* 1607.6 (M^+).

L4TC1. ^{1}H NMR (300 MHz, Chloroform-*d*) δ 7.74 (d, *J* = 5.2 Hz, 2H), 7.71-7.68 (m, 4H), 7.62 (s, 2H), 7.42 (t, *J* = 2.0 Hz, 2H), 4.19 (d, *J* = 5.3 Hz, 4H), 1.99-1.85 (m, 6H), 1.65 (dt, *J* = 13.3, 6.7 Hz, 10H), 1.40 (d, *J* = 7.3 Hz, 8H), 0.99 (dt, *J* = 18.8, 6.5 Hz, 28H), 0.68 (dt, *J* = 22.6, 6.9 Hz, 12H). ^{13}C NMR (100 MHz, Chloroform-*d*) δ 179.00, 154.91, 149.92, 145.05, 142.13, 140.69, 140.48, 134.43, 132.77, 132.20, 130.75, 130.31, 123.45, 122.40, 121.42, 114.68, 113.13, 112.77, 72.15, 71.60, 52.68, 42.07, 39.59, 36.52, 34.48, 30.49, 29.45, 29.31, 28.07, 24.00, 22.98, 22.94, 14.04, 11.34, 11.24. MS

(MALDI-TOF): *m/z* 1243.8 (M^+).

General characterization

UV-Vis absorption spectra of solutions (in $CHCl_3$) and thin films (on quartz substrates) were recorded using a UH5700 spectrophotometer (Hitachi High-Tech Science Corporation). ^{1}H and ^{13}C nuclear magnetic resonance (NMR) spectra were collected on Bruker AVANCE (300 MHz and 400 MHz) spectrometers. Mass spectra were acquired using a Q Exactive GC mass spectrometer (Thermo Scientific) and an Autoflex III MALDI-TOF mass spectrometer (Bruker Daltonics). Ultraviolet photoelectron spectroscopy (UPS) measurements were performed on an AXIS ULTRA DLD system (Kratos) using He I (21.22 eV) excitation and a –9 V sample bias under a vacuum of 3.0×10^{-8} Torr. Low-energy inverse photoemission spectroscopy (LEIPS) was conducted on a customized ULVAC-PHI instrument operated in Bremsstrahlung isochromatic mode.

Molecular modelling

Density functional theory (DFT) calculations were performed using the Gaussian 16 package at the B3LYP/6-31G(d,p) level (*54*), with molecular geometries optimized after replacing long alkyl side chains with methyl groups to reduce computational cost. The Multiwfn wavefunction analysis tool was employed to calculate the Mulliken electronegativity and electrostatic potential (ESP) distribution, while the charge transfer amount (Q_{CT}) from fragment D to fragment A during molecular electron excitation was determined using the interfragment charge transfer (IFCT) method. The geometry optimization of isolated dimer molecules was performed using DFT-D (density functional theory including the D3 version of Grimme's dispersion correction (*30*)) to accurately account for weak intermolecular interactions, with long alkyl side chains replaced by methyl groups to reduce computational cost. Intermolecular binding energies based on the optimized dimers were calculated with correction for basis set superposition error (BSSE) (*29*).

Grazing incidence wide-angle x-ray scattering measurement

Grazing Incidence Wide-Angle X-ray Scattering (GIWAXS) measurements were carried out using a Xenocs-SAXS/WAXS system equipped with a Pilatus3 R 300K two-dimensional detector and an X-ray wavelength of 1.5418 Å. Samples were prepared by spin coating on silicon wafers. The molecular stacking distance (d-spacing) was calculated using Eq. S1:

$$d = 2\pi/q \tag{S1}$$

The crystalline coherence length (CCL) was determined based on Scherrer analysis using the Eq. S2 (*55*):

$$\mathrm{CCL} = 2\pi K/\mathrm{FWHM} \tag{S2}$$

where FWHM is the full width at half maximum of the corresponding diffraction peak and *K* is the shape factor (set to 0.9).

Space-charge limited current measurement

Electron-only devices were fabricated with the architecture of ITO/ZnO/active layer (acceptor or PTB7-Th: acceptor)/PDINN/Ag. The acceptor layer was deposited by spin-coating its CB solution (24 mg mL^{-1}) onto the ZnO layer at 1400 rpm for 30 s. For blend films (PTB7-Th: acceptor), the active layer was prepared under the same conditions as the OPD devices. Subsequently, a PDINN layer (1 mg mL^{-1} in methanol) was spin-coated on top at 4000 rpm for 30 s. Finally, a silver electrode (~80 nm) was thermally evaporated under high vacuum (~10^{-5} Pa).

Hole-only devices were fabricated using the structure ITO/PEDOT: PSS/PTB7-Th: acceptor/Au. A ~30 nm thick PEDOT: PSS layer was spin-coated onto the ITO substrate from an aqueous solution (Baytron P VP AI 4083) at 4000 rpm for 30 s and subsequently annealed at 150 °C for 15 min. The active layer was deposited under the same conditions as for the OPD devices. Gold electrodes (~30 nm) were then thermally evaporated at a slow rate (1 Å/5 s) under high vacuum (~10^{-5} Pa) to minimize Au penetration into the active layer.

The carrier mobility is extracted from the quadratic regime of the *J-V* curve by fitting the Mott–Gurney equation (*56*):

$$J = \frac{9}{8}\mu\varepsilon_0\varepsilon_r\frac{V^2}{L^3} \tag{S3}$$

where J is current density, μ is carrier mobility, ε_0 is the permittivity of free space, ε_r is the relative dielectric constant, L is the thickness of the active layer, and V is the effective voltage, defined as:

$$V = V_{appl} - V_{bi} \tag{S4}$$

where V_{appl} is the applied voltage, and V_{bi} is the built-in voltage.

Transient photovoltage and transient photocurrent measurements

Background illumination was provided by a solar simulator (Enlitech, SS-F5-3A) equipped with a KG5 filter, while pulsed excitation was delivered by a 1064 nm laser (AO-S-1064, CNI). Photovoltage transients were recorded using an oscilloscope (MDO3014, Tektronix) under open-circuit conditions. Photocurrent transients were measured with a 50 Ω load under short-circuit conditions. The integrated *transient photocurrent* (TPC) signal reflects the total charge generated by the solar simulator. Carrier lifetimes were extracted by fitting the Transient photovoltage (TPV) voltage-time (*V-t*) curves with an exponential decay model.

Noise-equivalent power measurement

Noise-equivalent power (NEP) measurements were conducted using the same instrumentation employed for noise current characterization. LEDs with wavelengths of 1.05 and 1.3 μm were used separately as illumination sources, each modulated at 7 Hz via a function generator. The optical power density was controlled by adjusting the LED driver voltage, while absolute intensity calibration was performed using a commercial InGaAs photodiode (Thorlabs FGA21). The reverse bias was applied using the Bias Voltage function of the SR570 current preamplifier, and the voltage magnitude was monitored using a multimeter.

Linear dynamic range (LDR) measurement

A 1.3 µm light-emitting diode (LED) was employed as the light source, and driven by the lock-in amplifier (Sine Scientific Instruments OE1022) at a modulation frequency of 10 kHz, with the incident light intensity varied stepwise by adjusting the driving voltage. The actual incident light intensity at each step was calibrated using a reference InGaAs photodiode (Thorlabs FGA21) placed at the same position under identical optical path and modulation conditions. Photocurrents of the devices were measured using the lock-in amplifier. For LDR characterization under reverse bias, the device is connected through an SR570 current preamplifier, with a reverse bias voltage applied via the Bias Voltage function. The output is fed into a lock-in amplifier to measure the voltage signal. The photocurrent at various light intensities is then obtained by multiplying the sensitivity setting of the SR570 by the voltage reading from the lock-in amplifier. The LDR is calculated as follows:

$$\mathrm{LDR} = 20 \times \log\frac{J_{\mathrm{max}}}{J_{\mathrm{min}}} \quad \text{(S5)}$$

Here, J_{max} and J_{min} represent the current density values at the highest and lowest light intensities within the linear response range.

Response time and cut-off frequency measurement

A 1.05 or 1.3 µm LED (20 mW cm^{-2}) was used as the illumination source and driven by a square wave voltage at a fixed frequency for response time measurements and at varying frequencies for cut-off frequency measurements. The output current from the photodetector was fed into a low-noise current preamplifier (Stanford SR570), converted into a voltage signal, and subsequently recorded by an oscilloscope (Tektronix MDO3014). The rise and fall times (t_{rise}/t_{fall}) as well as the −3 dB cut-off frequency ($f_{-3\ dB}$) were extracted from the recorded response curves. The response time under reverse bias was obtained by applying reverse bias to the device via the function generator functionality embedded in the oscilloscope.

Trap density measurements

Trap density of states (tDOS) was performed on Keysight 4980A LCR meter and analyzed by using the thermal admittance spectroscopy method. tDOS can be derived from angular frequency-dependent capacitance according to the following Eq. S6:

$$N_{\mathrm{t}}(E_{\omega}) = -\frac{V_{\mathrm{bi}}}{eW}\frac{dC}{d\omega}\frac{\omega}{k_B T} \quad \text{(S6)}$$

where V_{bi} and W are the built-in potential and depletion width, respectively, which were extracted from Mott-Schottky analysis. e is the elementary charge, C is the capacitance, k_B is the Boltzmann constant, and T is the temperature. ω is the angular frequency defined by the following formula:

$$E_{\omega} = k_B \mathrm{T} \ln\left(\frac{\omega_0}{\omega}\right) \quad \text{(S7)}$$

where ω_0 is the attempt-to-escape frequency (*57*).

Supplementary Figures

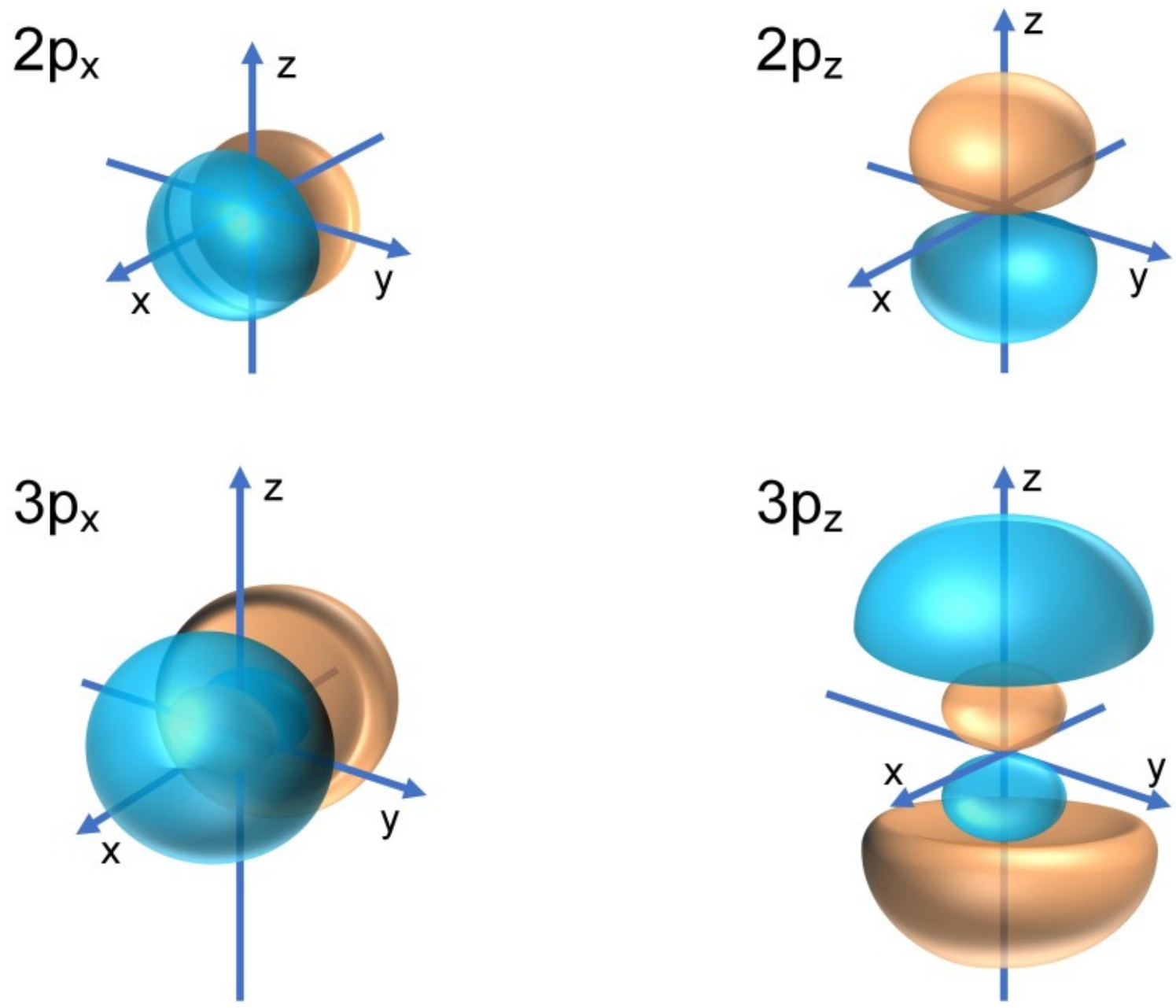

Fig. S1. Schematic representations of the valence p-orbitals: the $2p_x/2p_z$ orbitals of the carbon atom and the $3p_x/3p_z$ orbitals of the sulfur atom.

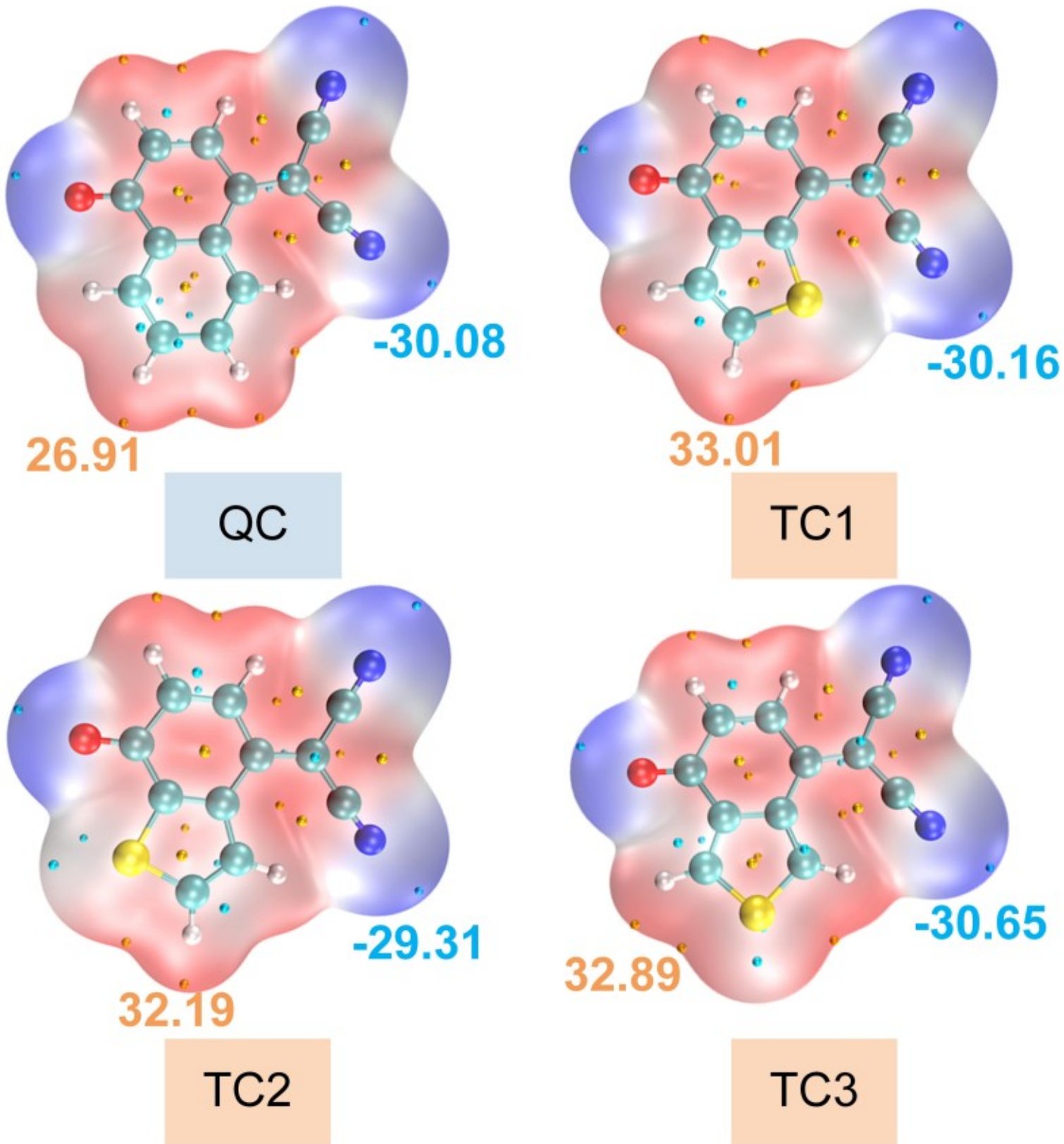

Fig. S2. ESP maps of the QC and TCx (x = 1, 2, 3) terminal groups. The orange spheres represent the local maxima of the potential, and the blue spheres represent the local minima. The values labeled in the maps correspond to the highest and lowest potential values among all extrema, with units of kcal mol^{-1}.

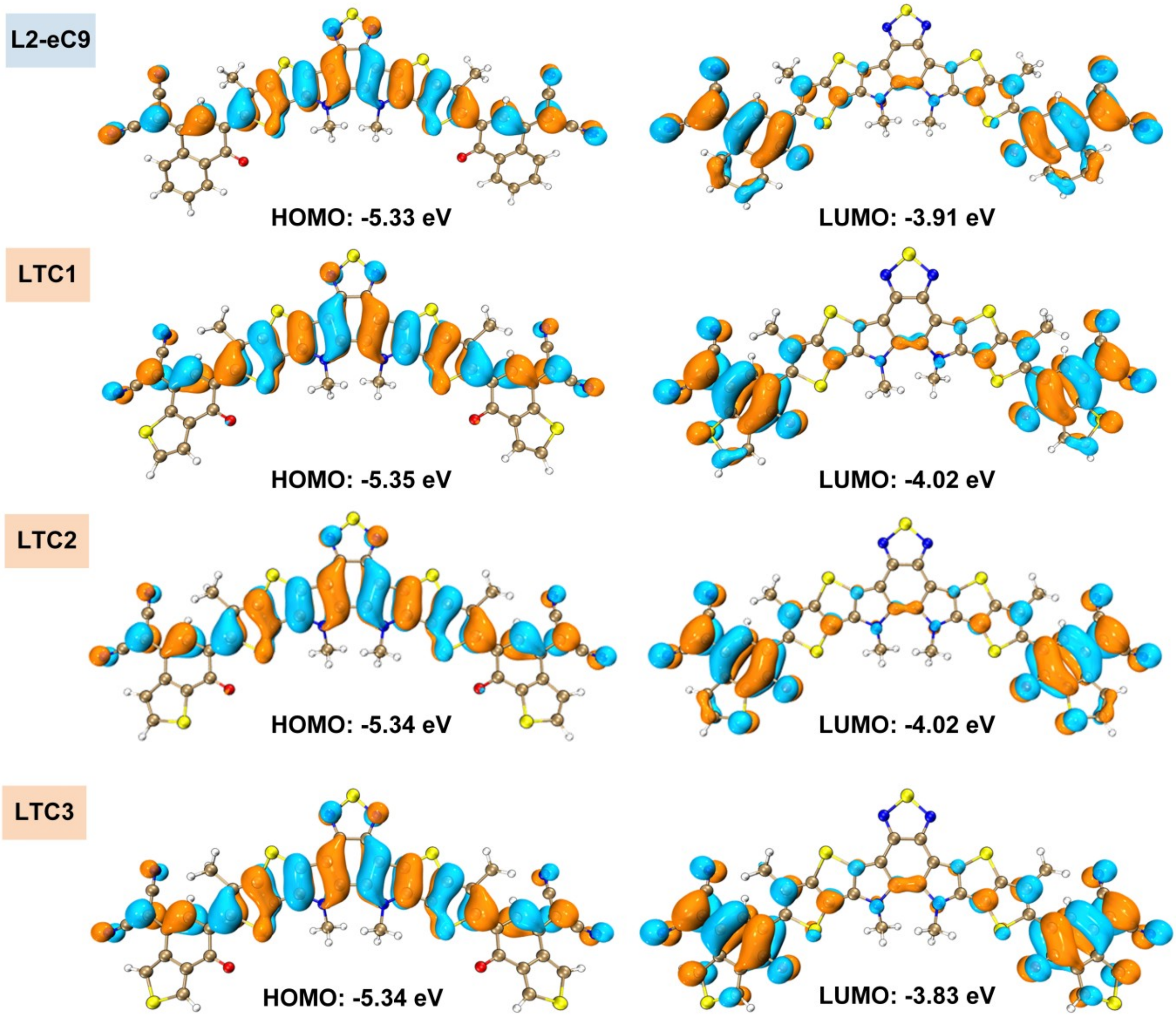

Fig. S3. Calculated HOMO and LUMO orbitals and energy levels of L2-eC9 and LTCx (x = 1, 2, 3).

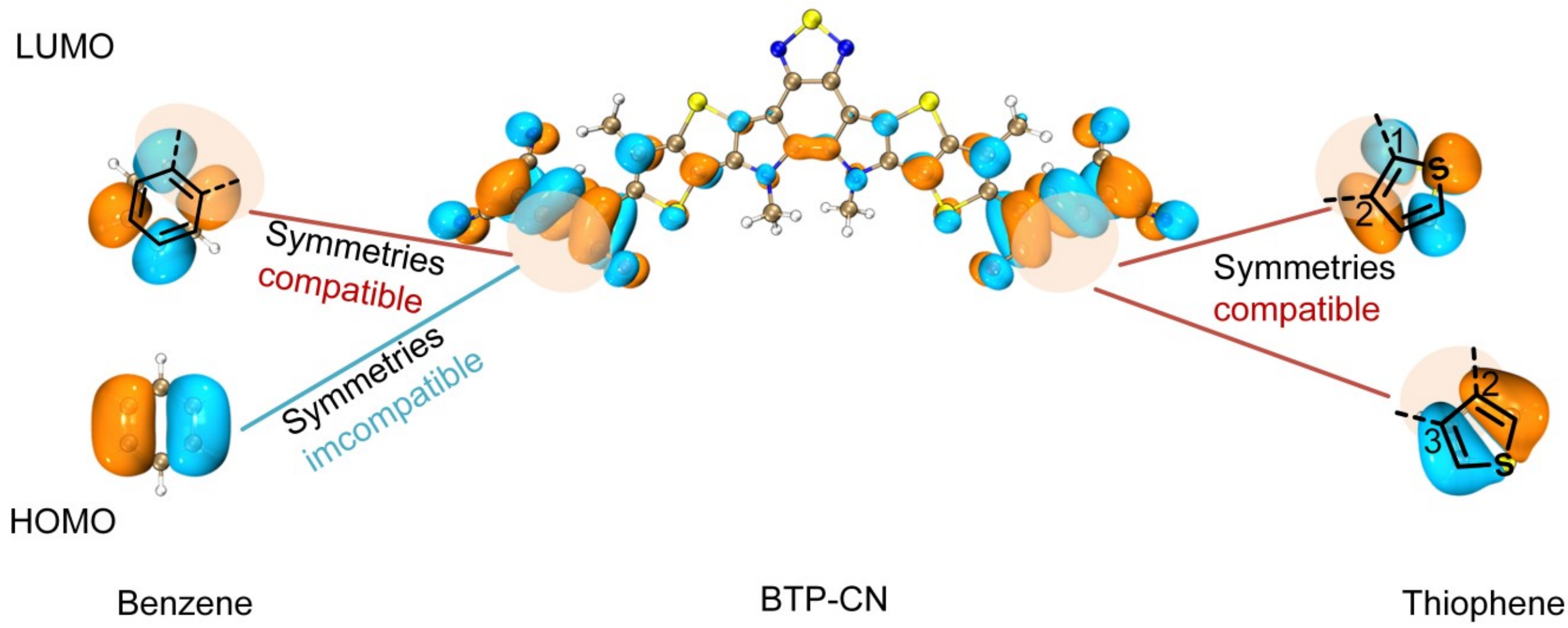

Fig. S4. Frontier molecular orbitals of thiophene, benzene, and BTP-CN calculated by DFT, along with schematic diagrams illustrating their orbital symmetry matching during molecular orbital combination.

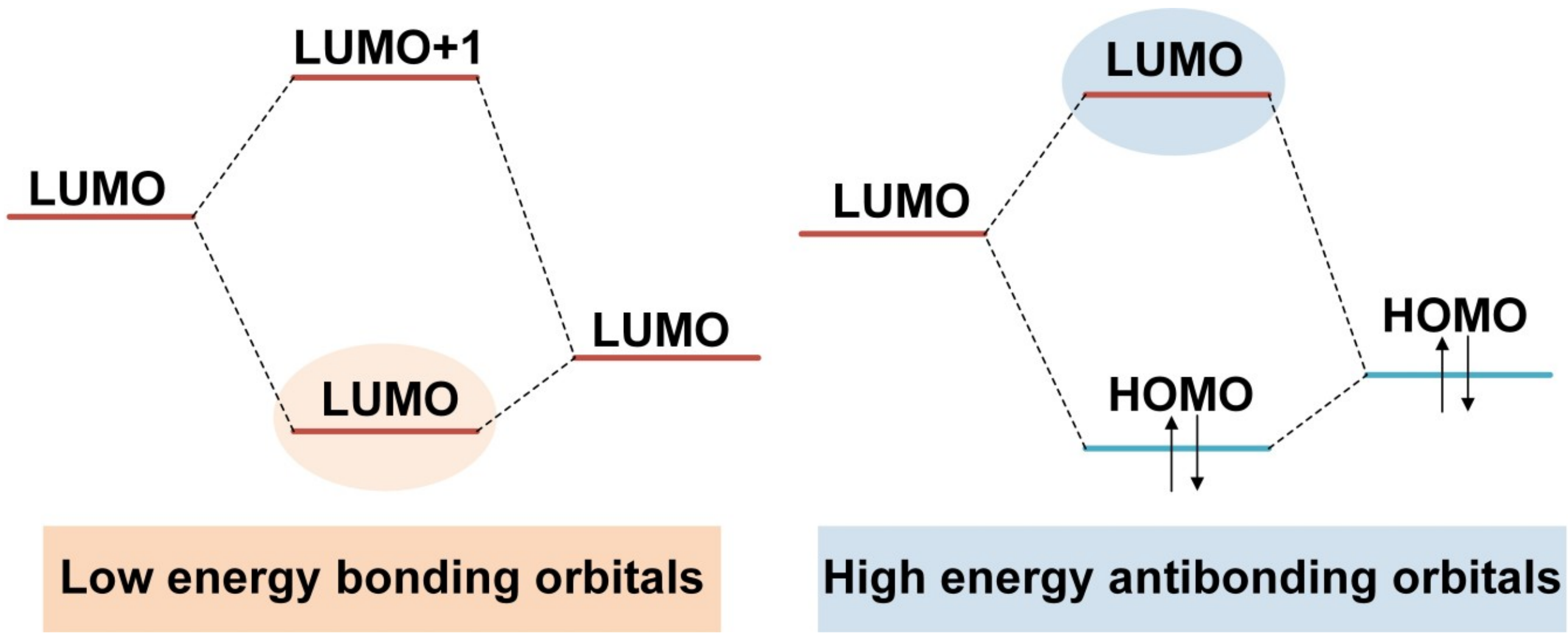

Fig. S5. Schematic illustration of the composition of molecular orbitals.

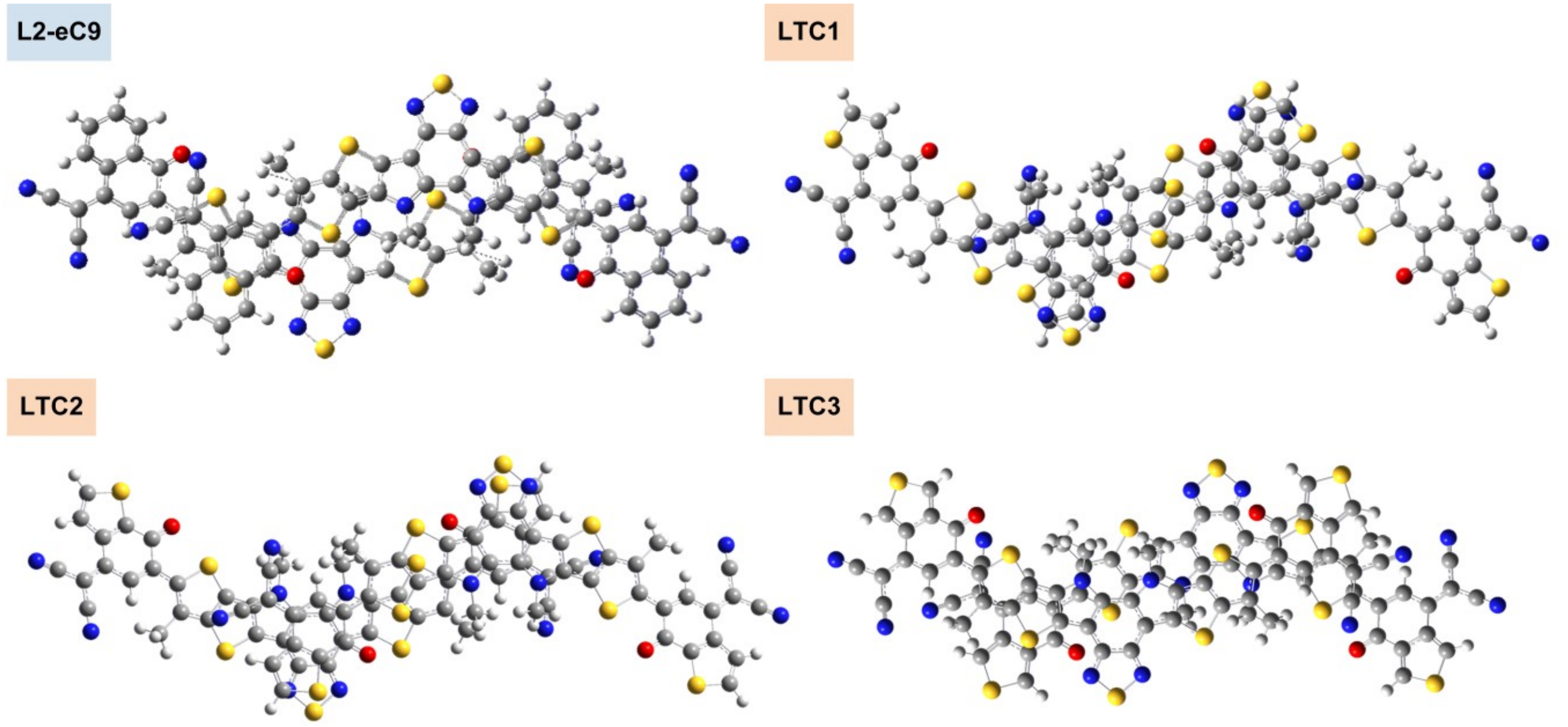

Fig. S6. Optimized DFT-D calculated packing geometry of L2-eC9 and LTCx (x=1, 2, 3) dimers.

Synthetic route

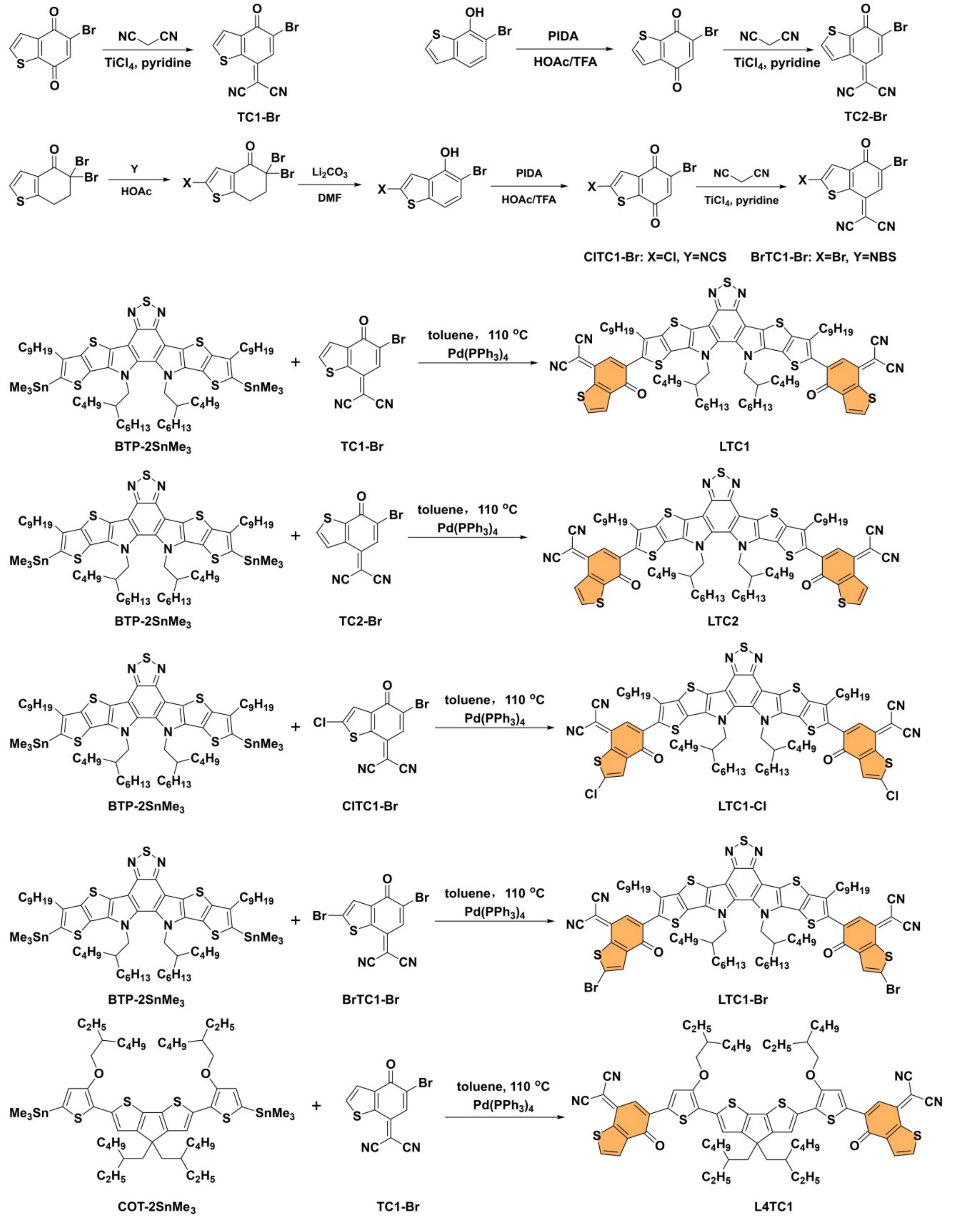

Fig. S7. Synthetic routes for TCx-Br (x=1, 2), ClTC1-Br, BrTC1-Br, LTC1, LTC2, LTC1-Cl, LTC1-Br, and L4TC1.

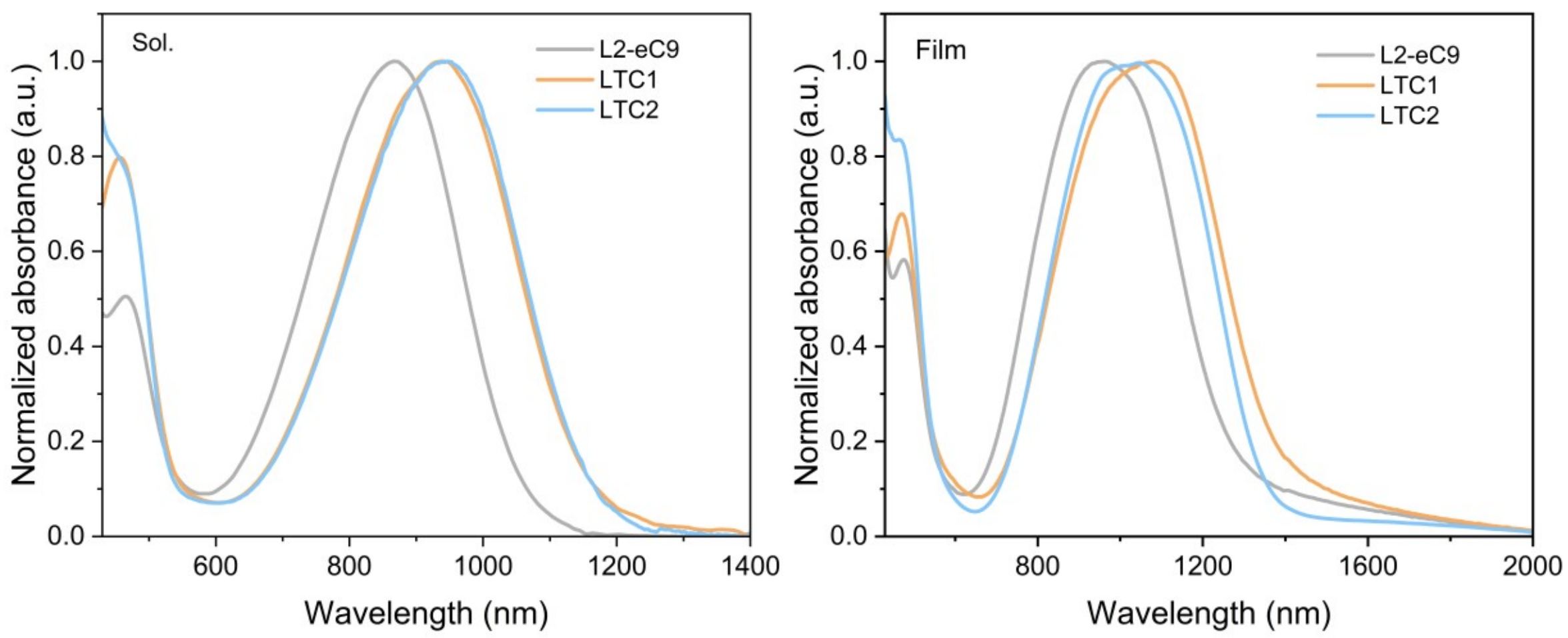

Fig. S8. Absorption spectra of L2-eC9, LTC1, and LTC2 in dilute chloroform solution and $CHCl_3$-cast thin films.

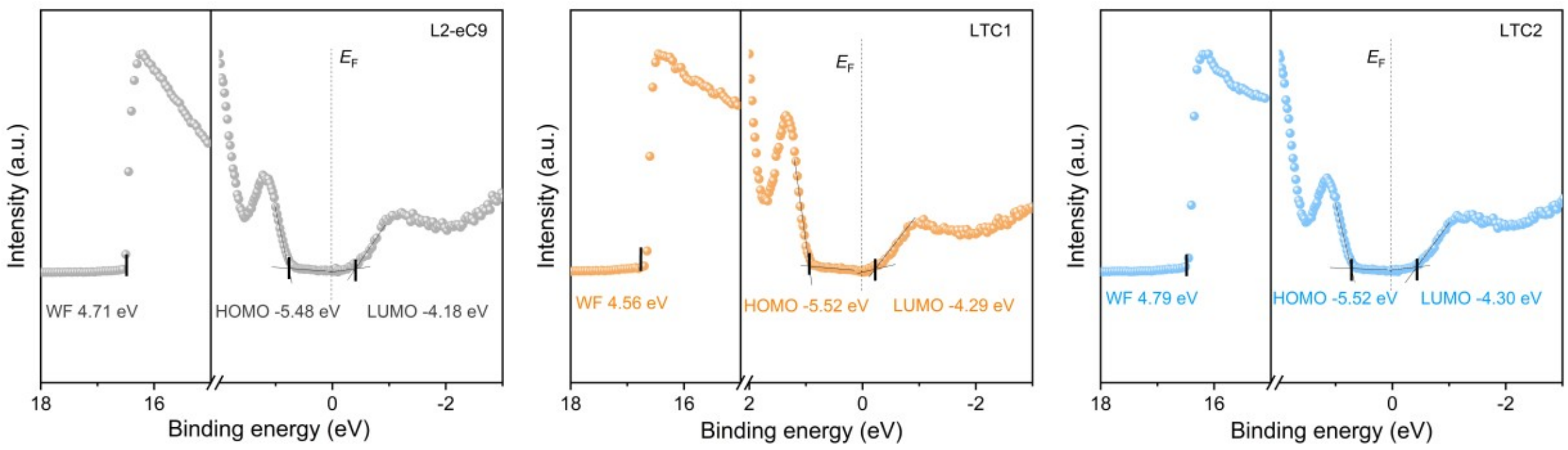

Fig. S9. UPS and LEIPS spectra of L2-eC9, LTC1, and LTC2 with respect to the E_F at 0 eV. Work function (WF) values are labeled.

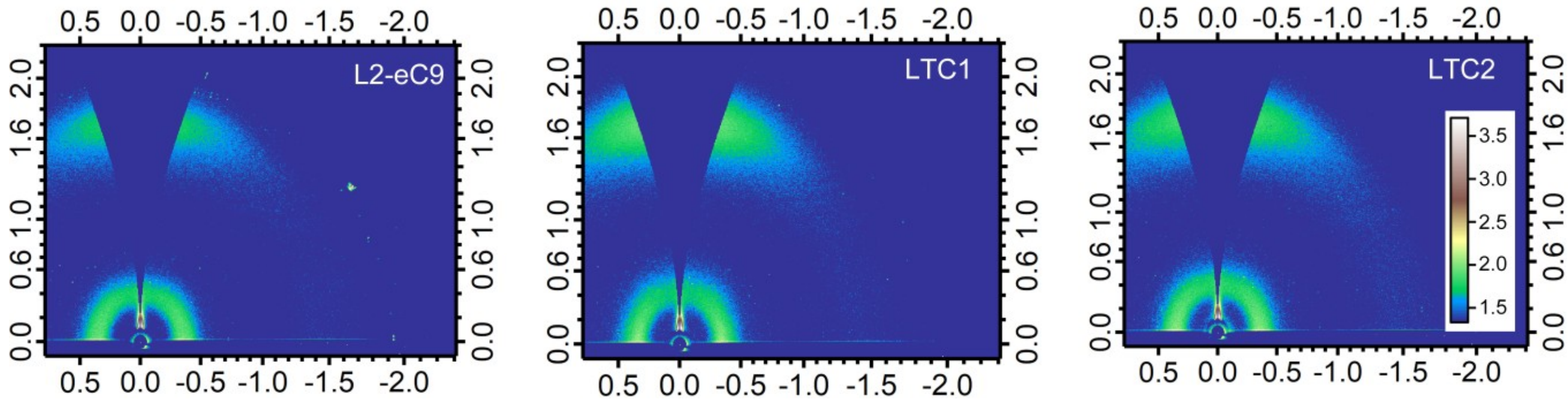

Fig. S10. 2D GIWAXS patterns of L2-eC9, LTC1, and LTC2 films.

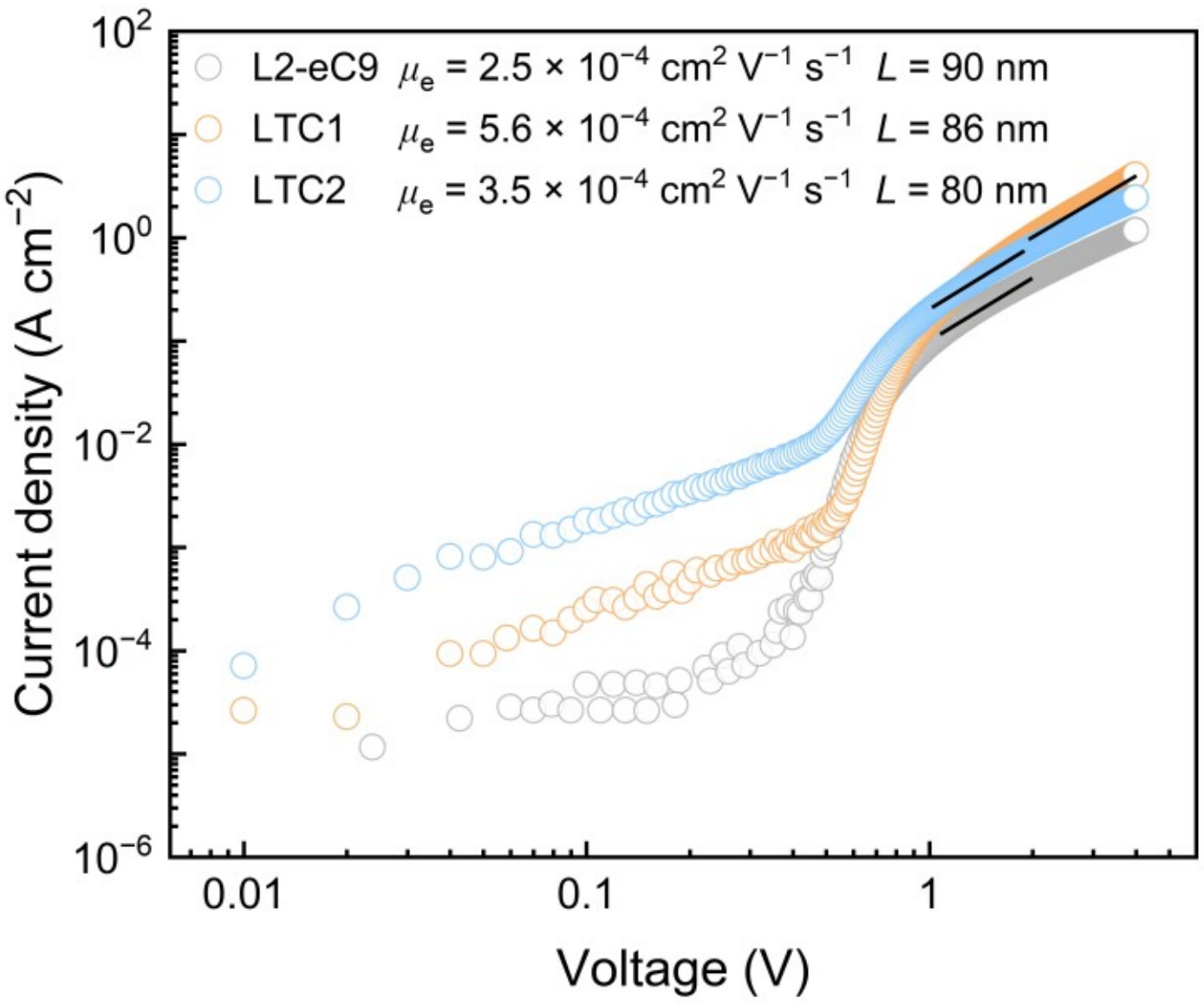

Fig. S11. Current density-voltage curves in the dark for electron-only devices based on L2-eC9, LTC1, and LTC2.

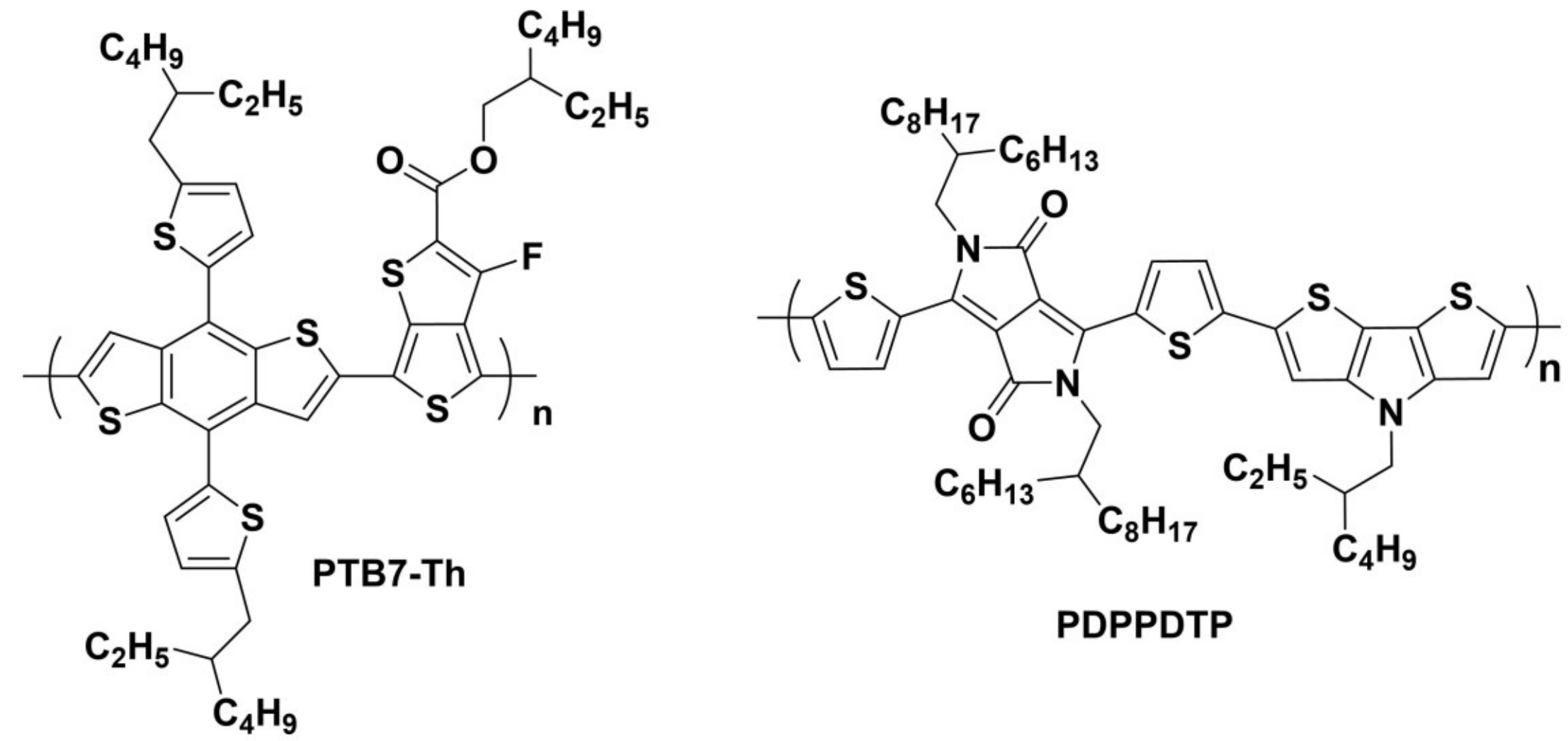

Fig. S12. Chemical structures of PTB7-Th and PDPPDTP.

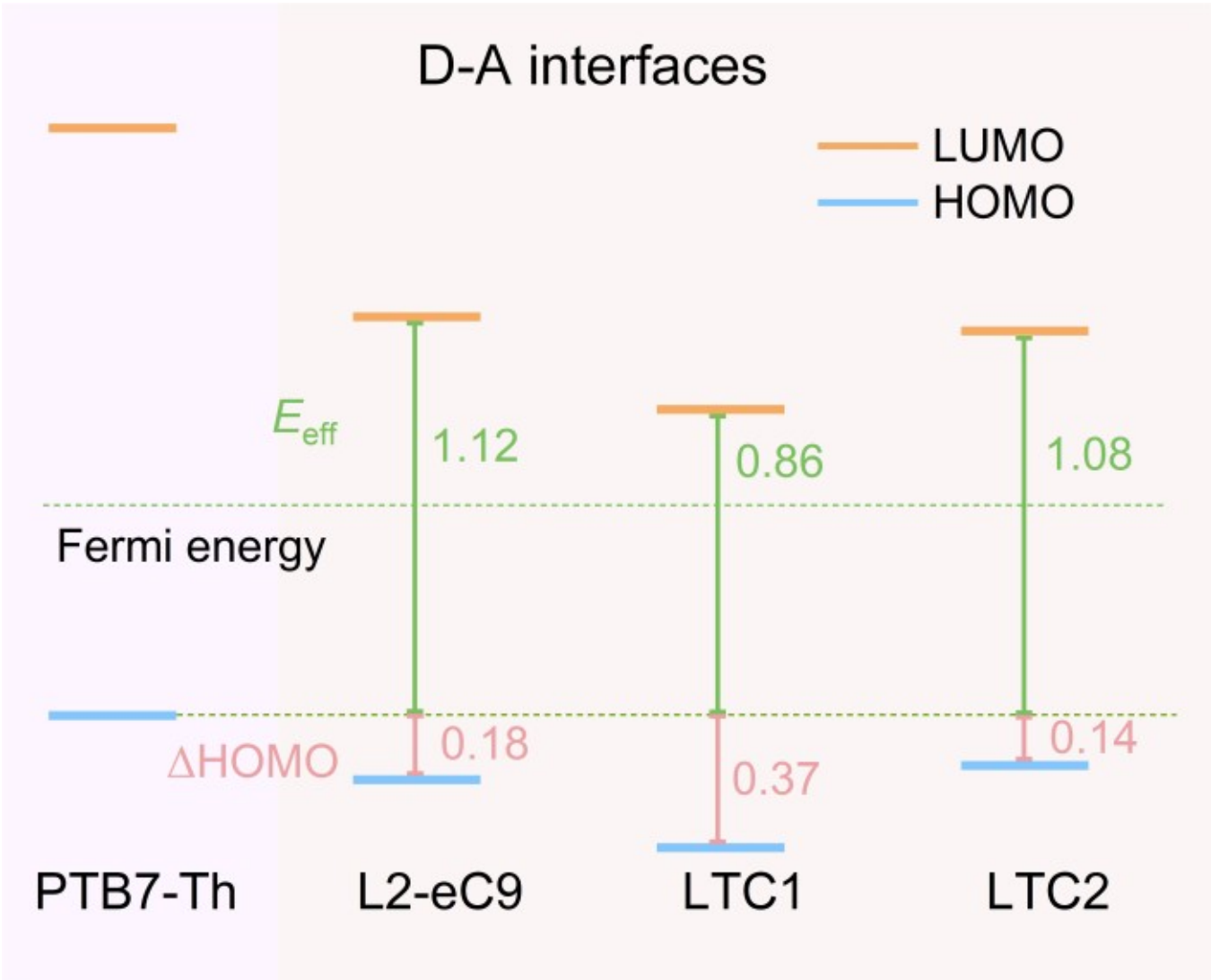

Fig. S13. E_{eff} and HOMO offset between donor PTB7-Th and acceptor (ΔHOMO) values at different D-A interfaces.

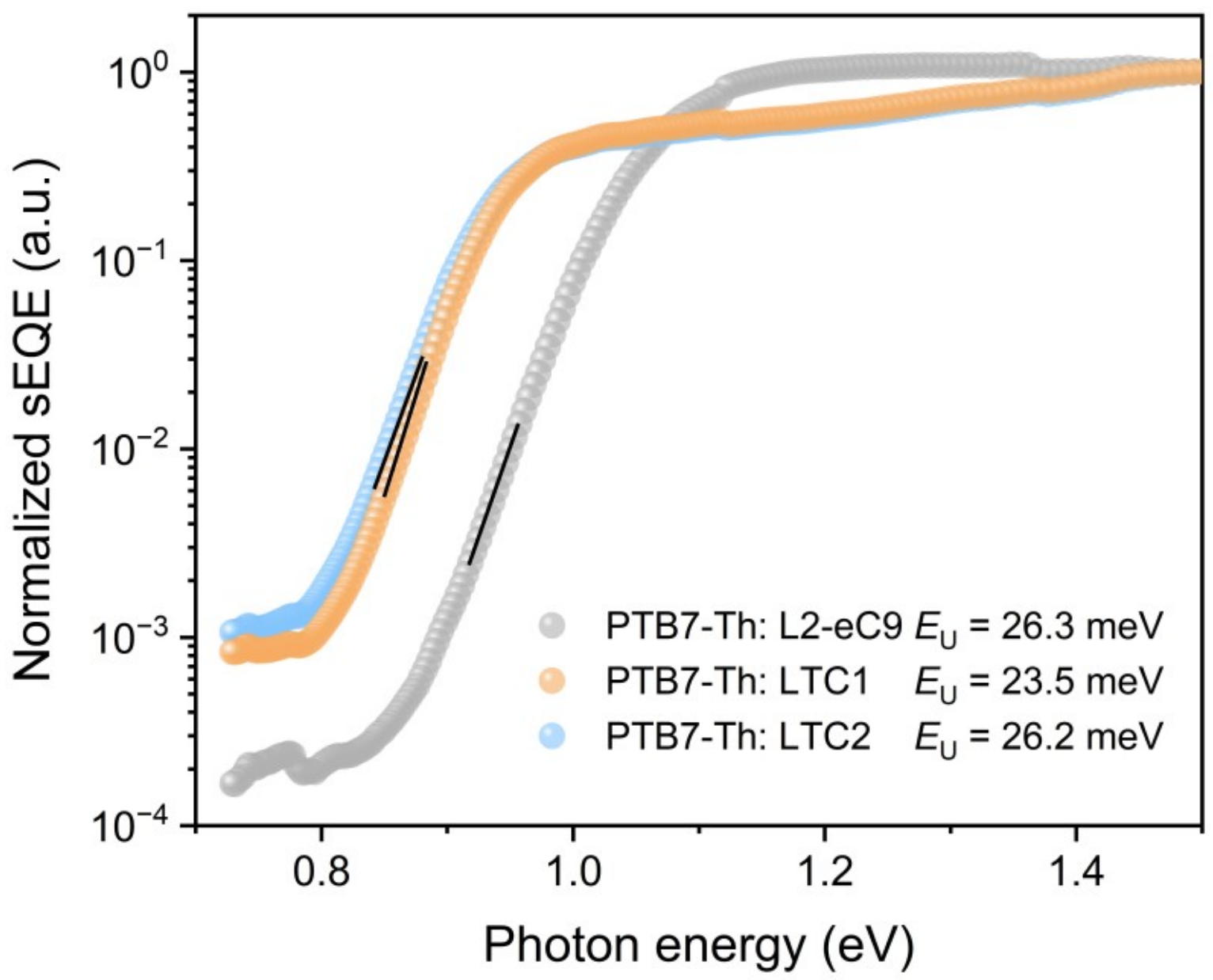

Fig. S14. Normalized sEQE spectra and the exponential fitting of E_U for SWIR OPDs based on PTB7-Th: L2-eC9, PTB7-Th: LTC1, and PTB7-Th: LTC2.

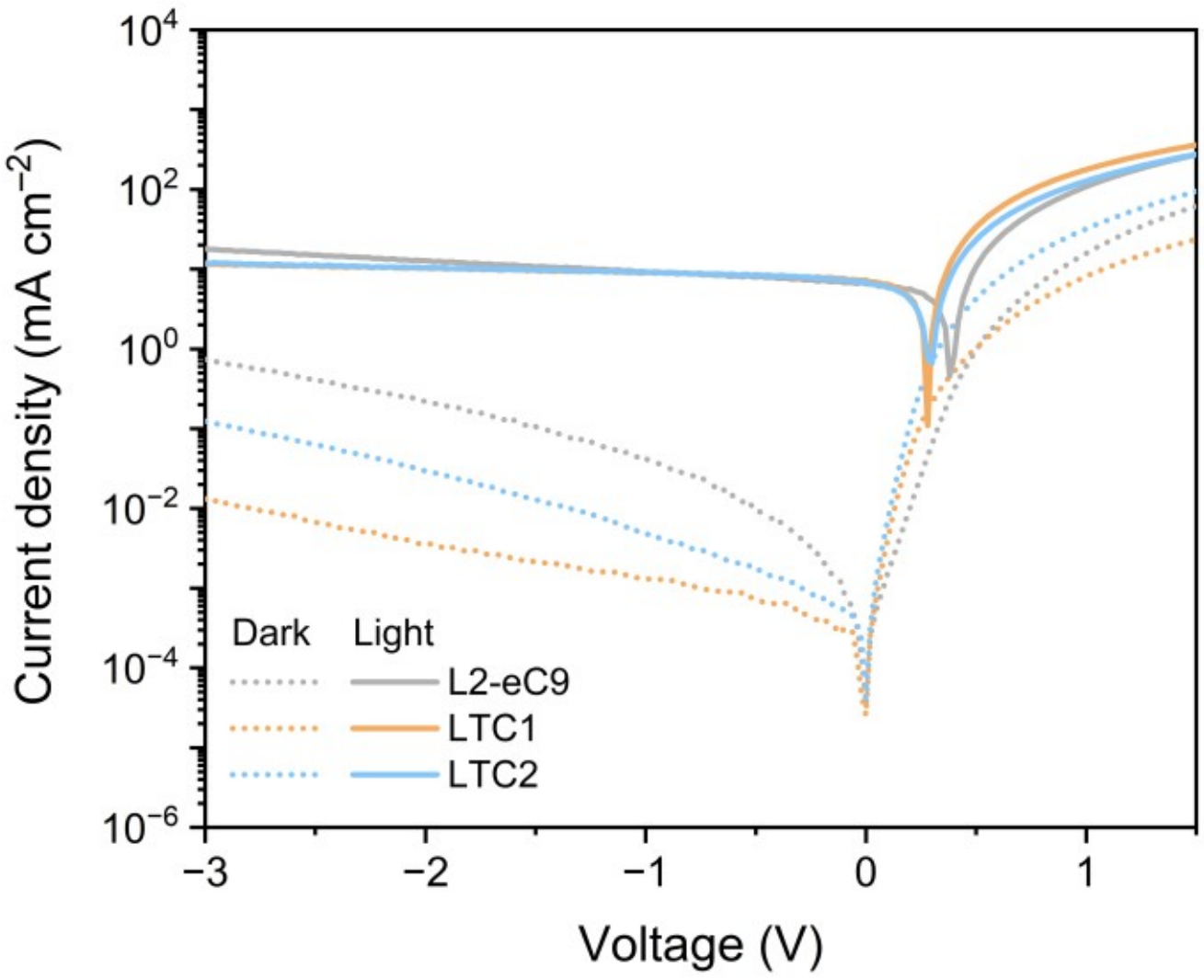

Fig. S15. ***J-V*** **characteristics measured in dark and under AM 1.5G irradiation (100 mW cm^{-2}) of SWIR OPDs based on PTB7-Th: L2-eC9 and PTB7-Th: LTCx (x= 1, 2).**

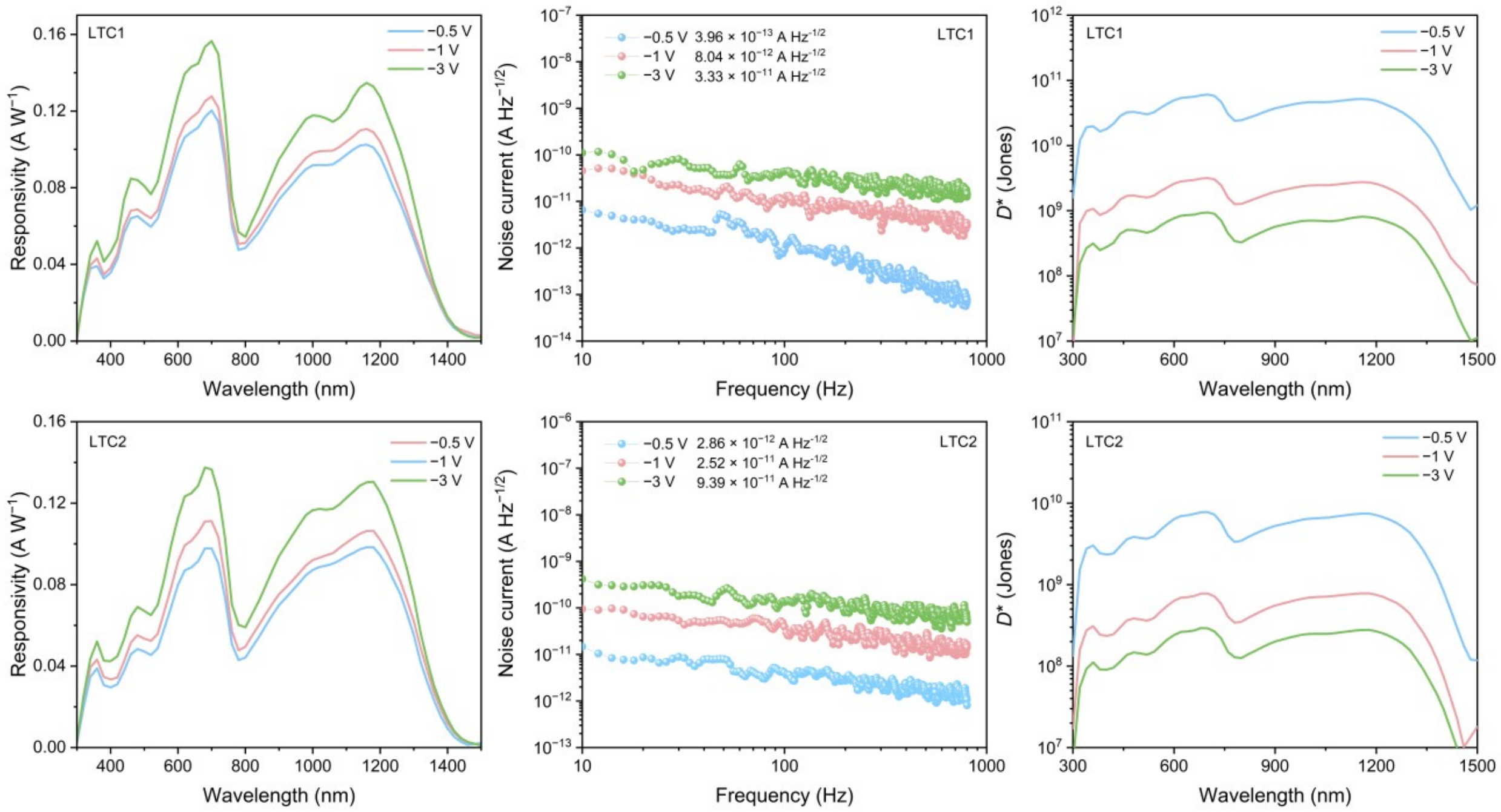

Fig. S16. Responsivity, noise current, and specific detectivity of SWIR OPDs based on PTB7-Th: LTC1 and PTB7-Th: LTC2 under reverse bias conditions. The noise current used for D^* calculation was extracted at 210 Hz, consistent with the modulation frequency employed in EQE measurements.

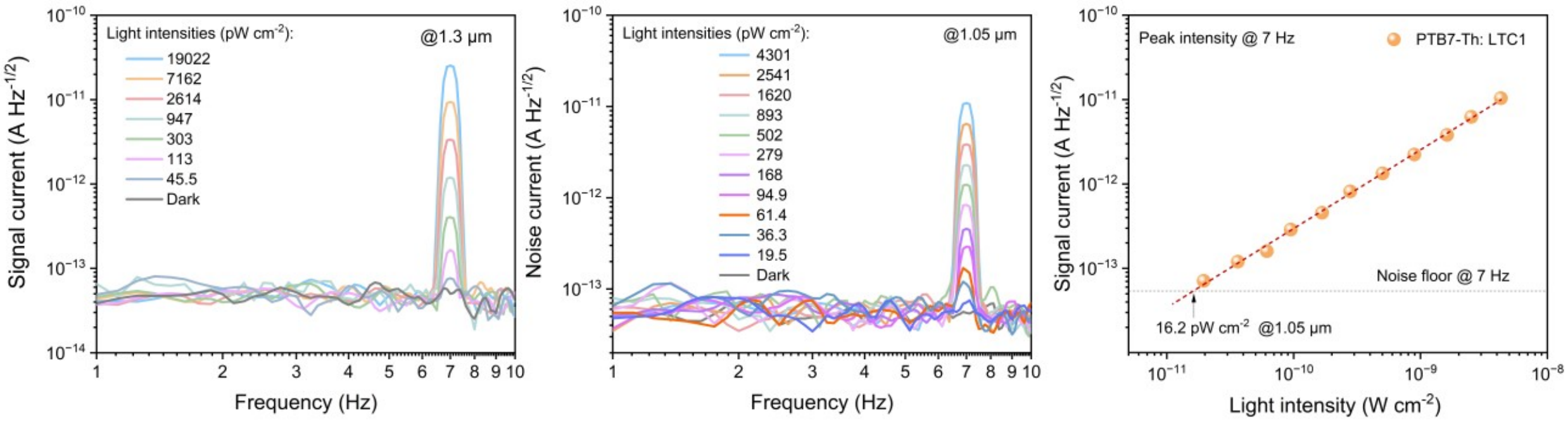

Fig. S17. Signal currents under 1.05 μm and 1.3 μm light illumination modulated at 7 Hz with various light intensities for PTB7-Th: LTC1-based SWIR OPDs operating at zero bias, and the corresponding peak signal intensities at 7 Hz as a function of light intensities for 1.05 μm.

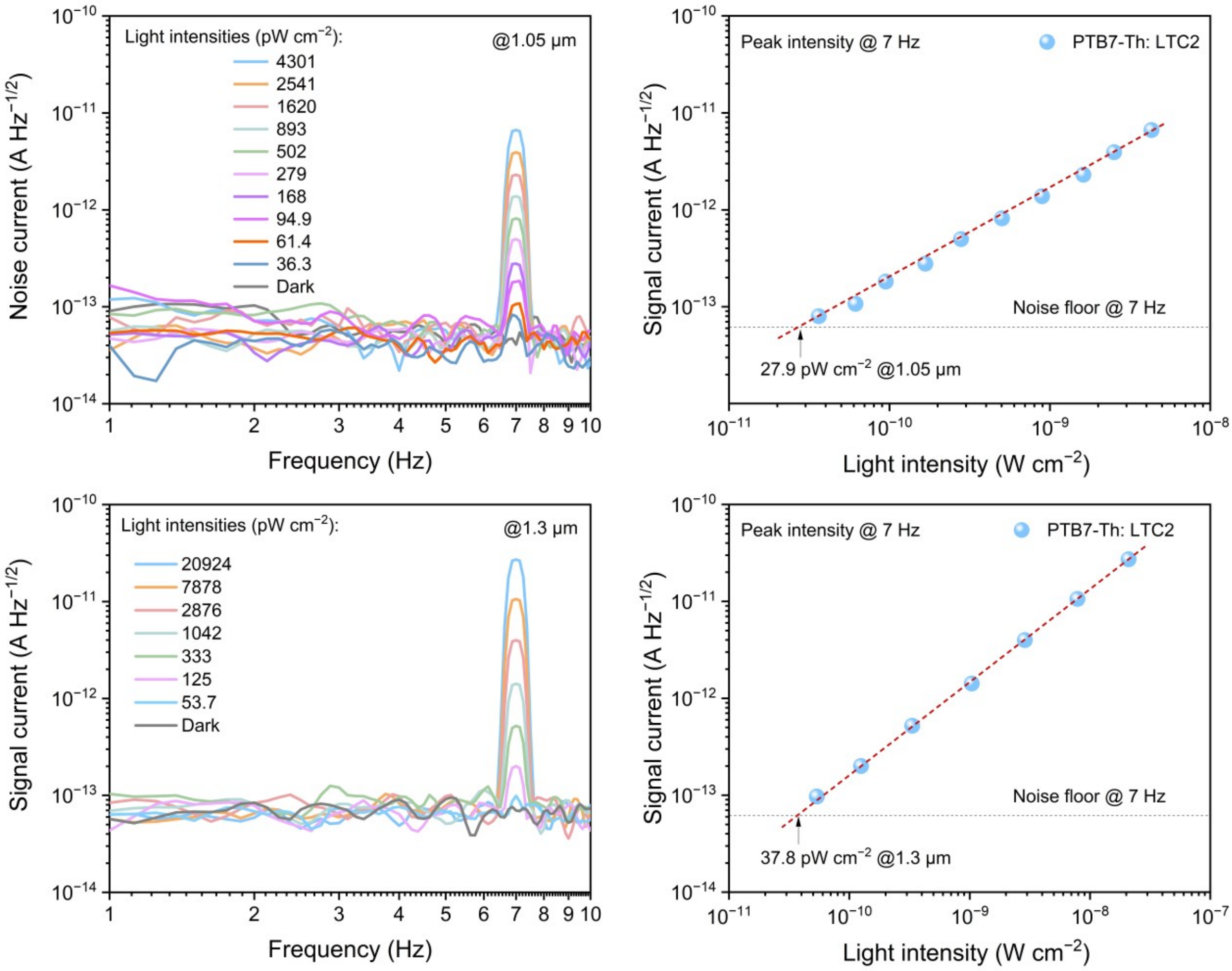

Fig. S18. Signal currents of PTB7-Th: LTC2-based SWIR OPDs operating at zero bias under 1.05 μm and 1.3 μm light illumination modulated at 7 Hz at various light intensities, and the corresponding peak signal intensities at 7 Hz as a function of light intensity.

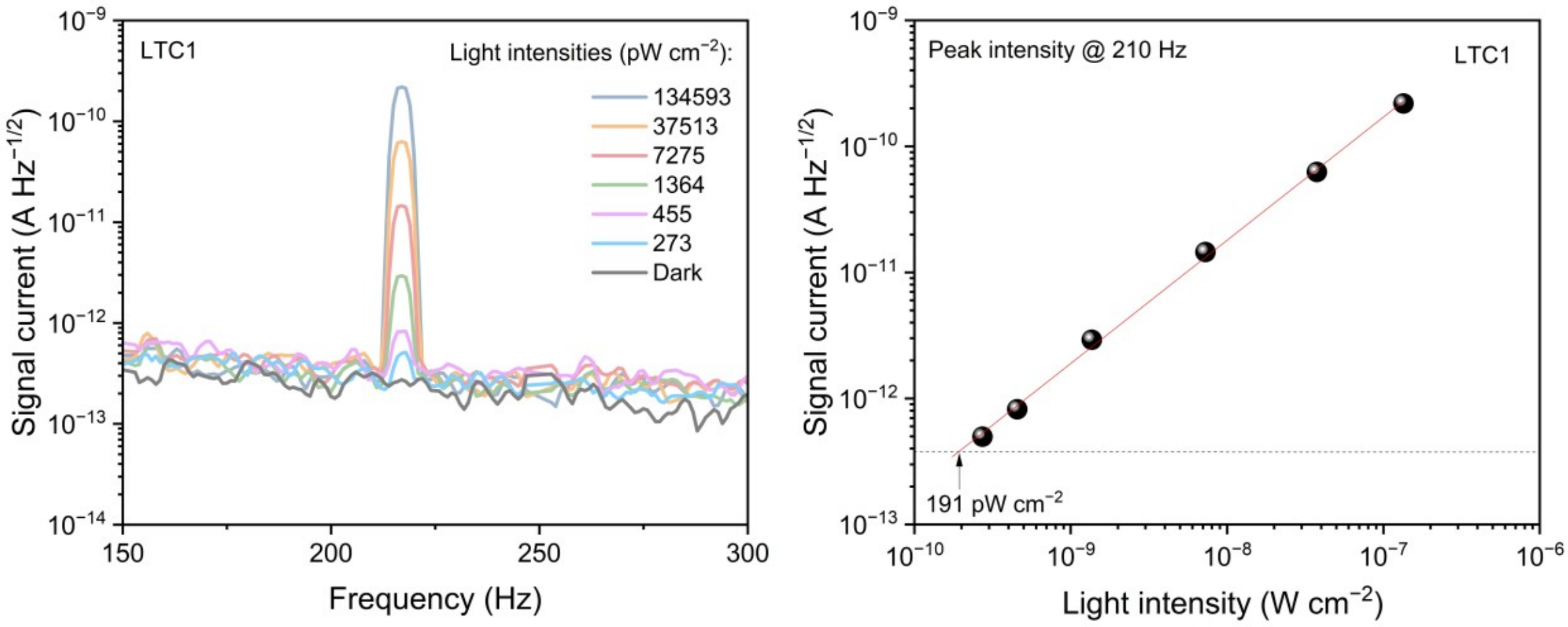

Fig. S19. Signal currents of PTB7-Th: LTC1-based SWIR OPDs measured under a reverse bias of −0.5 V and 1.3 μm light illumination modulated at 210 Hz over a range of light intensities, together with the corresponding peak signal intensities at 210 Hz as a function of light intensity.

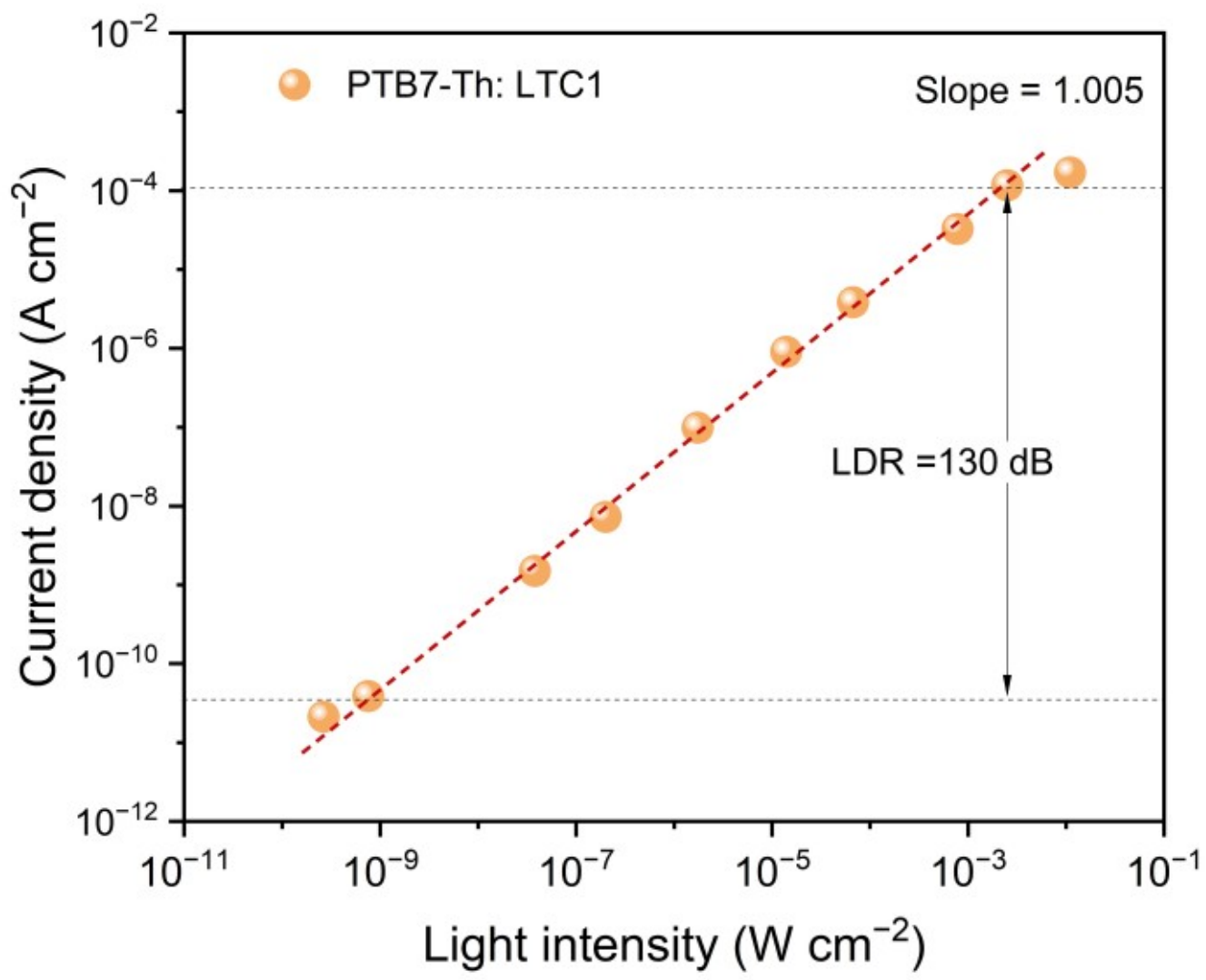

Fig. S20. LDR measurements of PTB7-Th: LTC1-based SWIR OPDs under a reverse bias of −0.5 V at 1.3 μm with various light intensities.

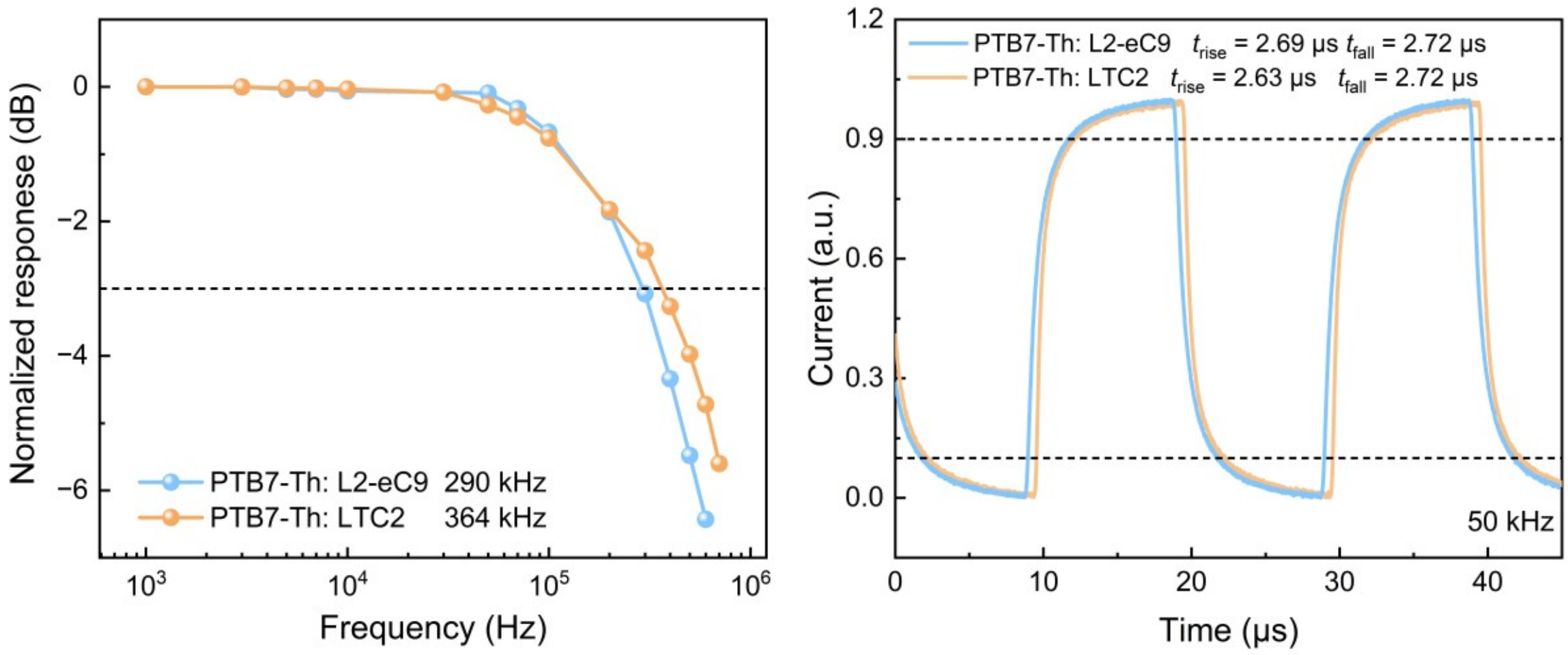

Fig. S21. The cut-off frequency and response time of OPDs based on PTB7-Th: L2-eC9 operating at zero bias under 1.05 μm LED illumination and PTB7-Th: LTC2 under 1.3 μm LED illumination.

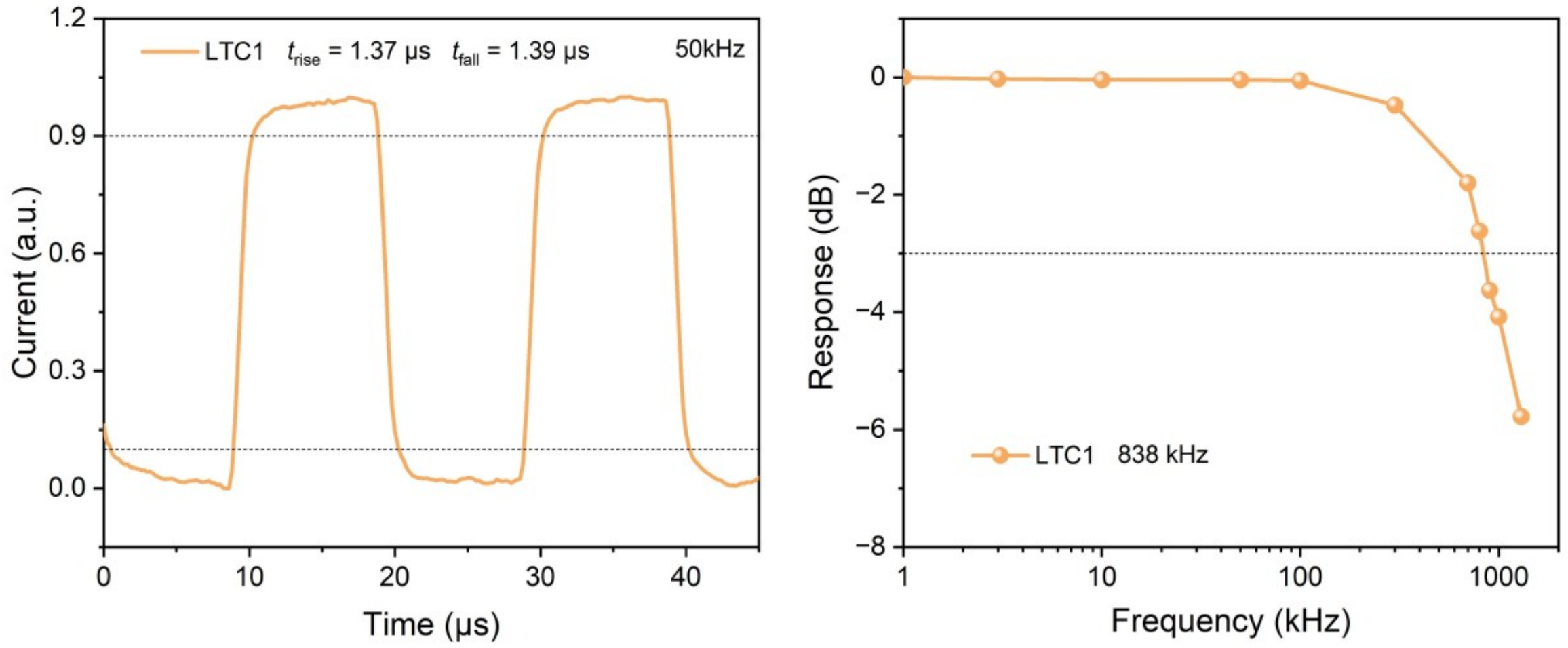

Fig. S22. The response time and cut-off frequency of OPDs based on PTB7-Th: LTC1 under a reverse bias of −0.5 V and 1.3 μm LED illumination.

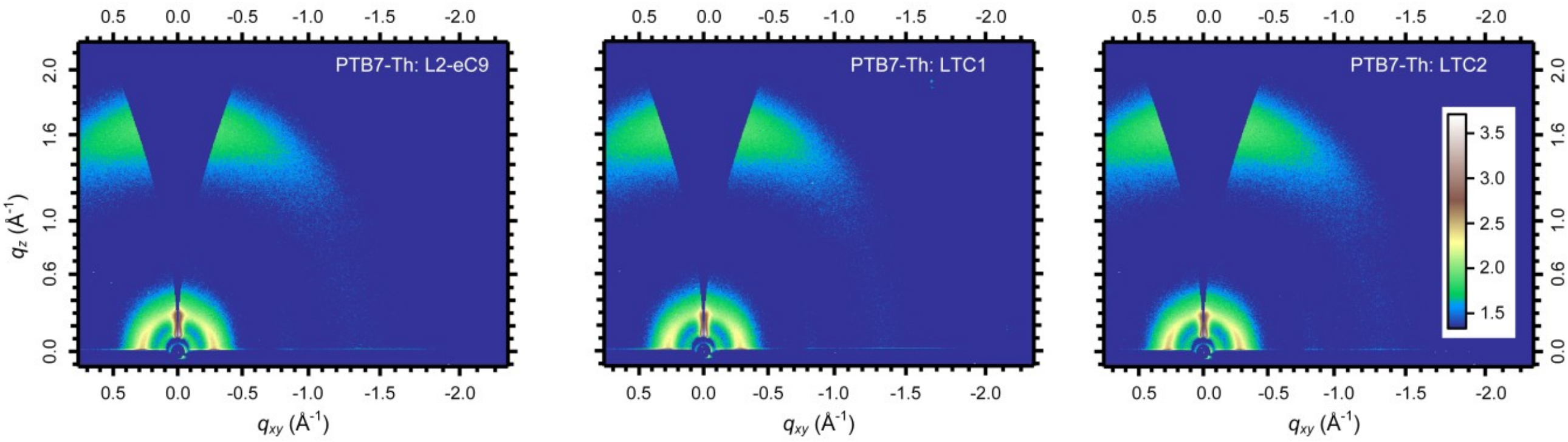

Fig. S23. 2D GIWAXS patterns of PTB7-Th: L2-eC9, PTB7-Th: LTC1, and PTB7-Th: LTC2 blend films.

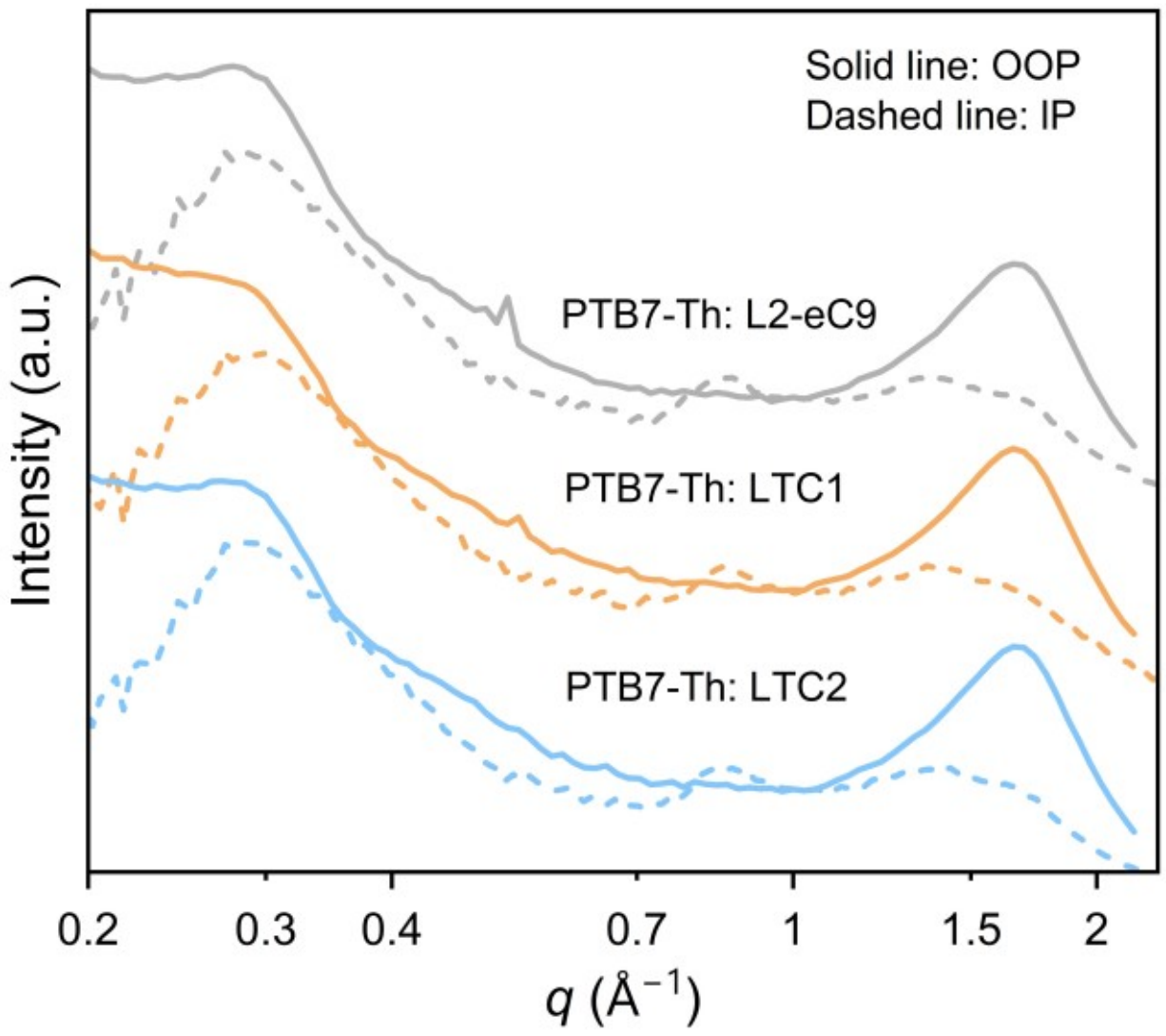

Fig. S24. Line-cut profiles in the IP and OOP directions of PTB7-Th: L2-eC9, PTB7-Th: LTC1, and PTB7-Th: LTC2 blend films.

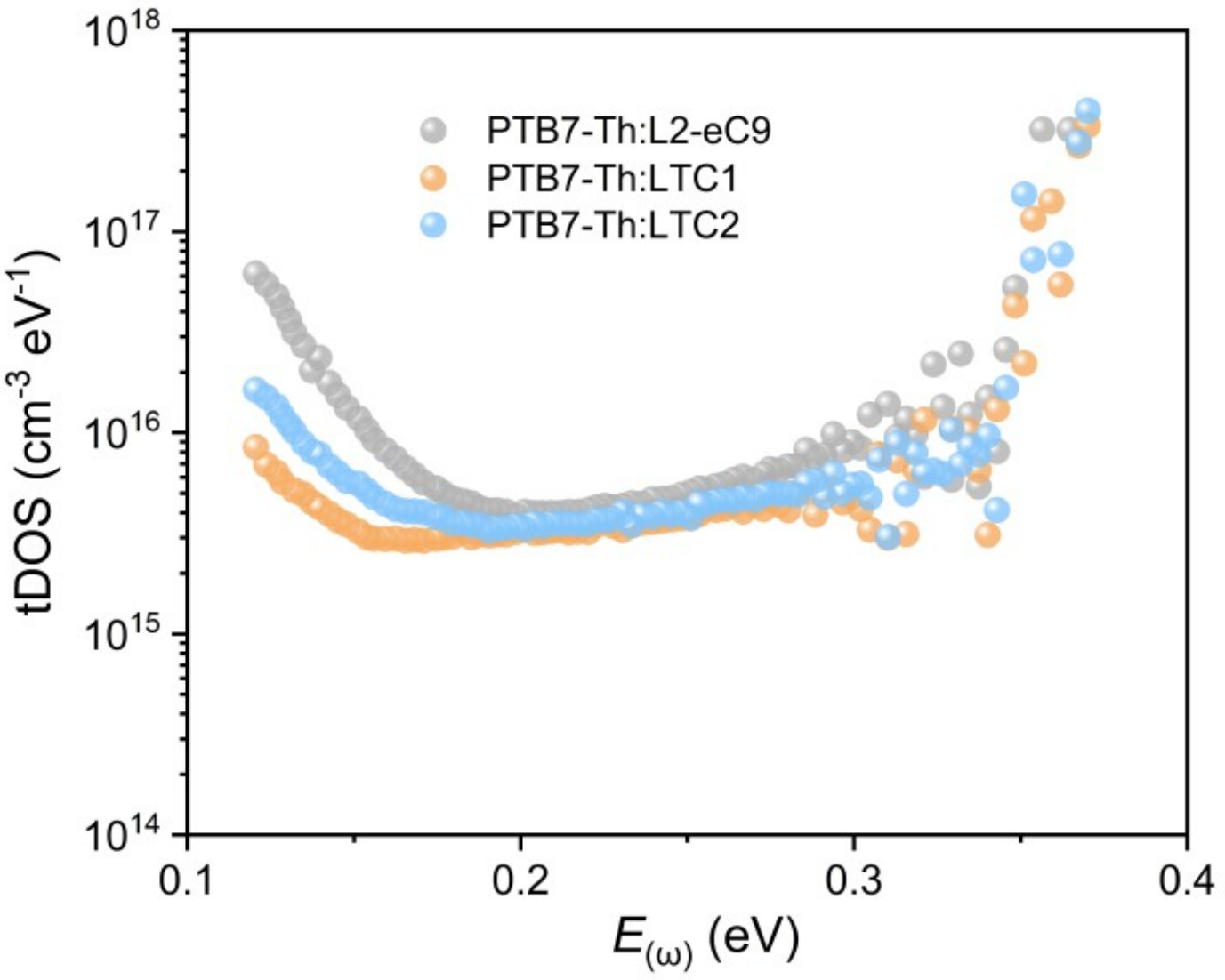

Fig. S25. The tDOS curves of the OPDs based on PTB7-Th: L2-eC9, PTB7-Th: LTC1, and PTB7-Th: LTC2.

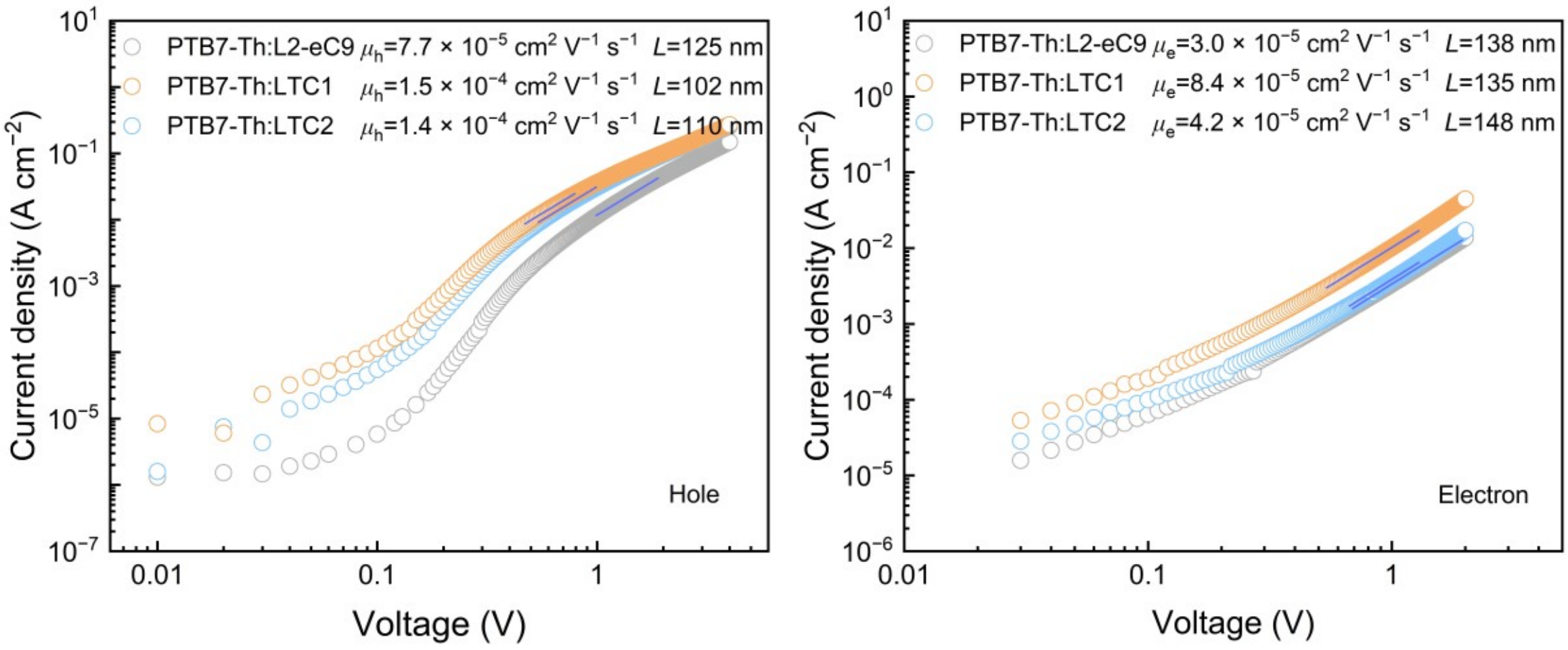

Fig. S26. ***J-V*** **curves in the dark for hole-only and electron-only devices based on PTB7-Th: L2-eC9 and PTB7-Th: LTCx (x=1, 2).**

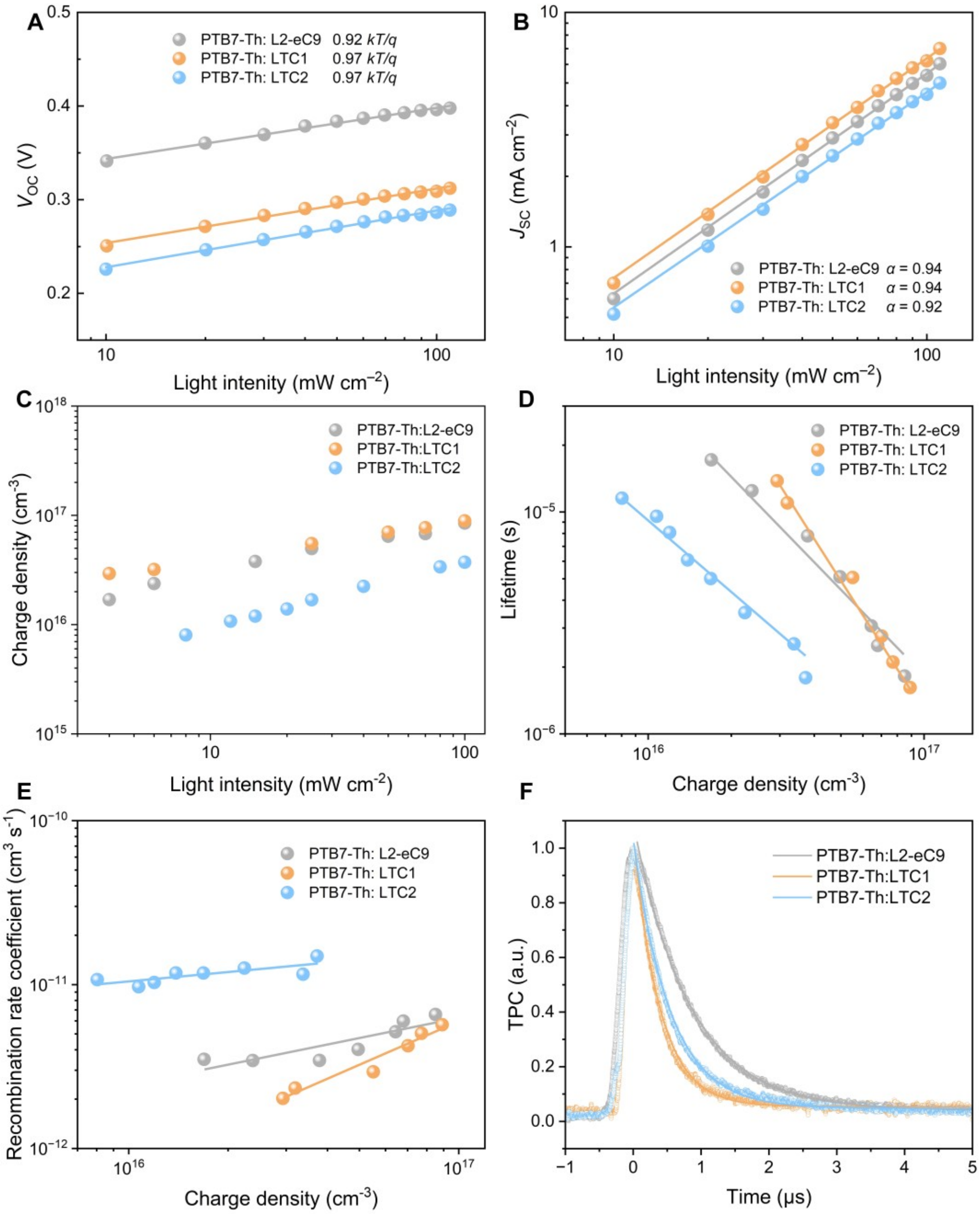

Fig. S27. Carrier dynamics of OPDs. Light-intensity dependence of (**A**) V_{OC} and (**B**) J_{SC} for OPDs based on PTB7-Th: L2-eC9, PTB7-Th: LTC1, and PTB7-Th: LTC2. Charge density versus light intensity (**C**), charge lifetime versus charge density (**D**), carrier recombination rate coefficient versus charge density (**E**), and TPC curves (**F**) under 1064 nm excitation of the same OPDs. The recombination orders of the PTB7-Th: L2-eC9, PTB7-Th: LTC1, and PTB7-Th: LTC2 devices are 2.40, 2.88, and 2.19, derived from the slopes of 0.40, 0.88, and 0.19 obtained by log-log fitting in (E), respectively.

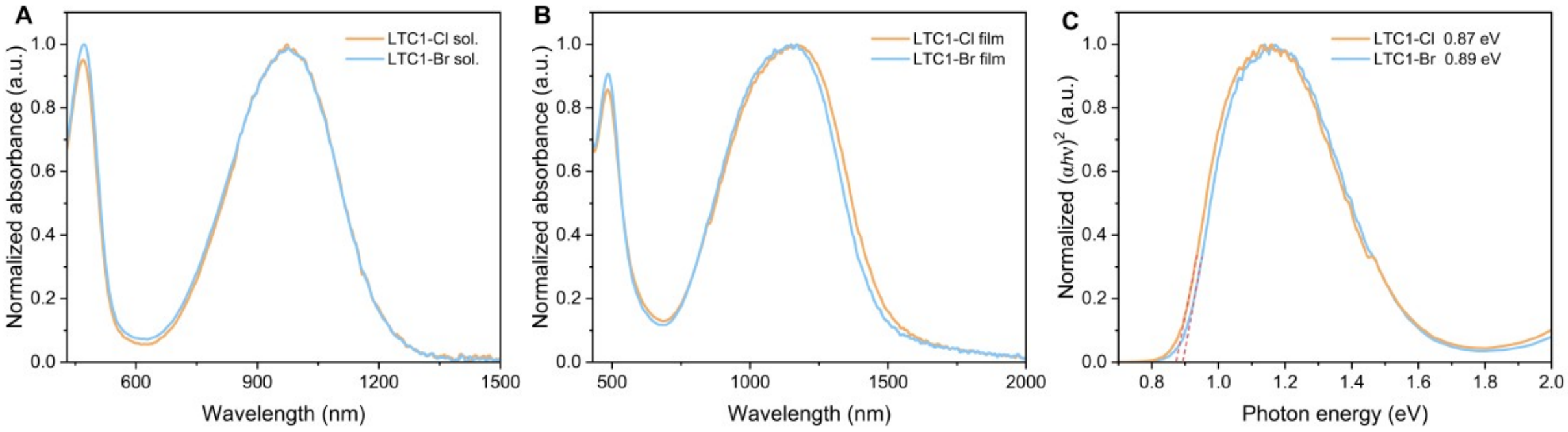

Fig. S28. Absorption spectra and optical bandgaps of LTC1-Cl and LTC1-Br. A, Absorption spectra of LTC1-Cl and LTC1-Br in dilute chloroform solution. **B,** Absorption spectra of $CHCl_3$-cast thin films of LTC1-Cl and LTC1-Br. **C,** The corresponding E_g^{opt} values of LTC1-Cl and LTC1-Br.

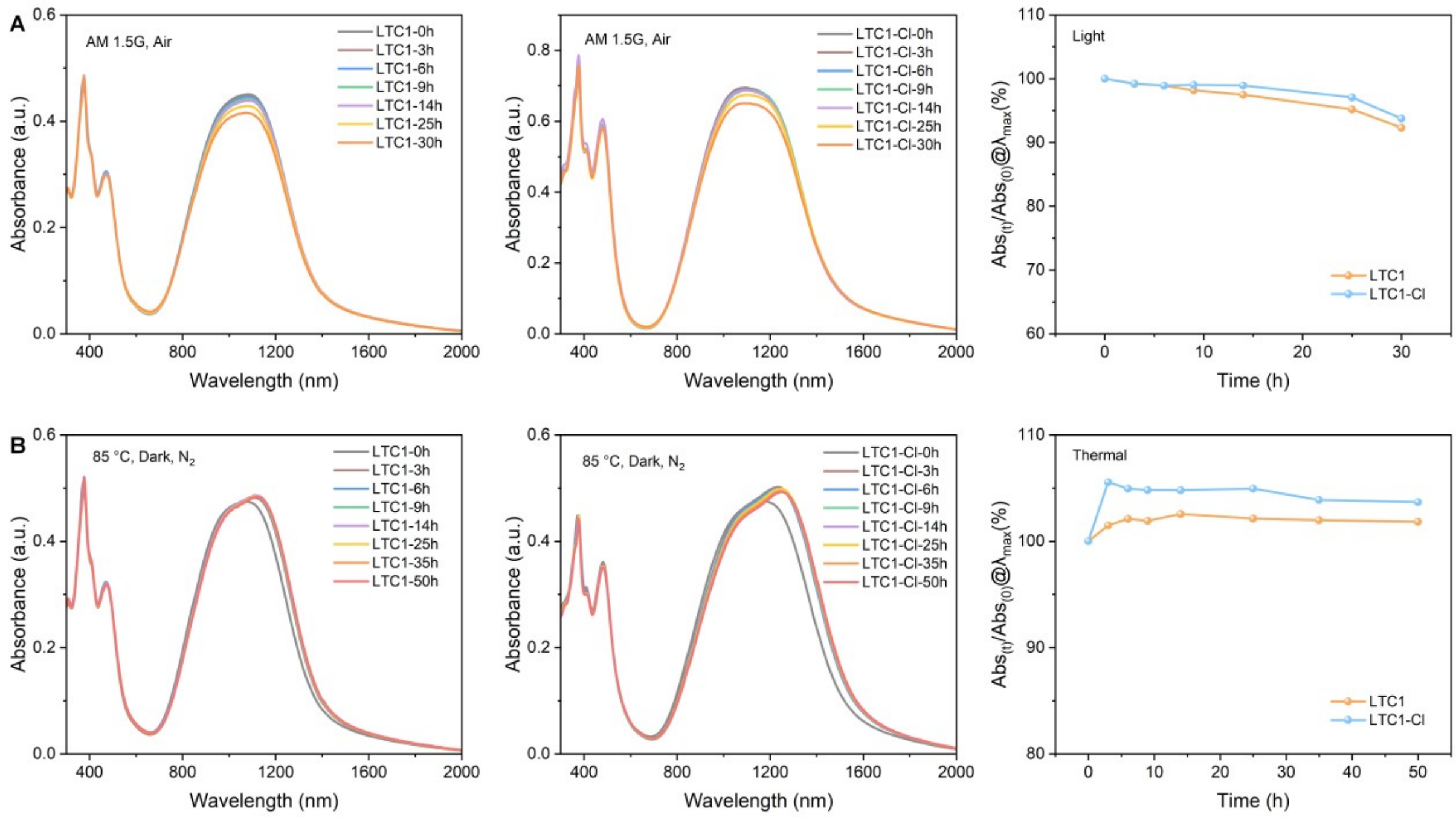

Fig. S29. Light and thermal stability of materials. The absorption spectra of LTC1 and LTC1-Cl thin films at different time and the corresponding absorption peak intensities normalized to their initial values under (**A**) continuous AM 1.5G irradiation (100 mW cm^{-2}) in air, and (**B**) dark conditions at 85 °C in N_2. Note: LTC1 film exhibited a moderate redshift in the maximum absorption peak accompanied by an enhancement in absorbance during the heating process, suggesting that thermal treatment may promote stronger intermolecular interactions and more ordered molecular packing or aggregation.

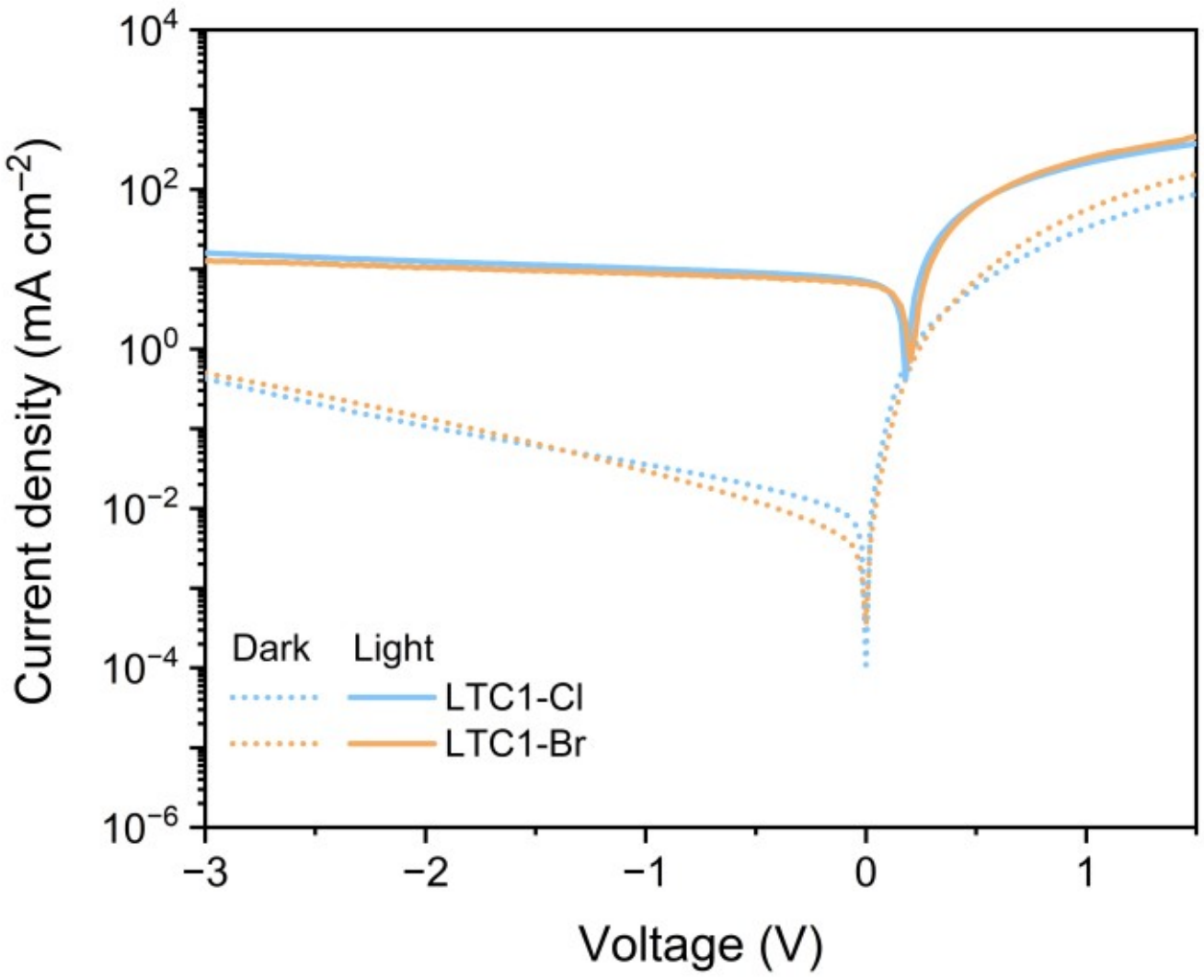

Fig. S30. ***J-V*** **characteristics measured in dark and under AM 1.5G irradiation (100 mW cm^{-2}) of SWIR OPDs based on PTB7-Th: LTC1-Cl and PTB7-Th: LTC1-Br.**

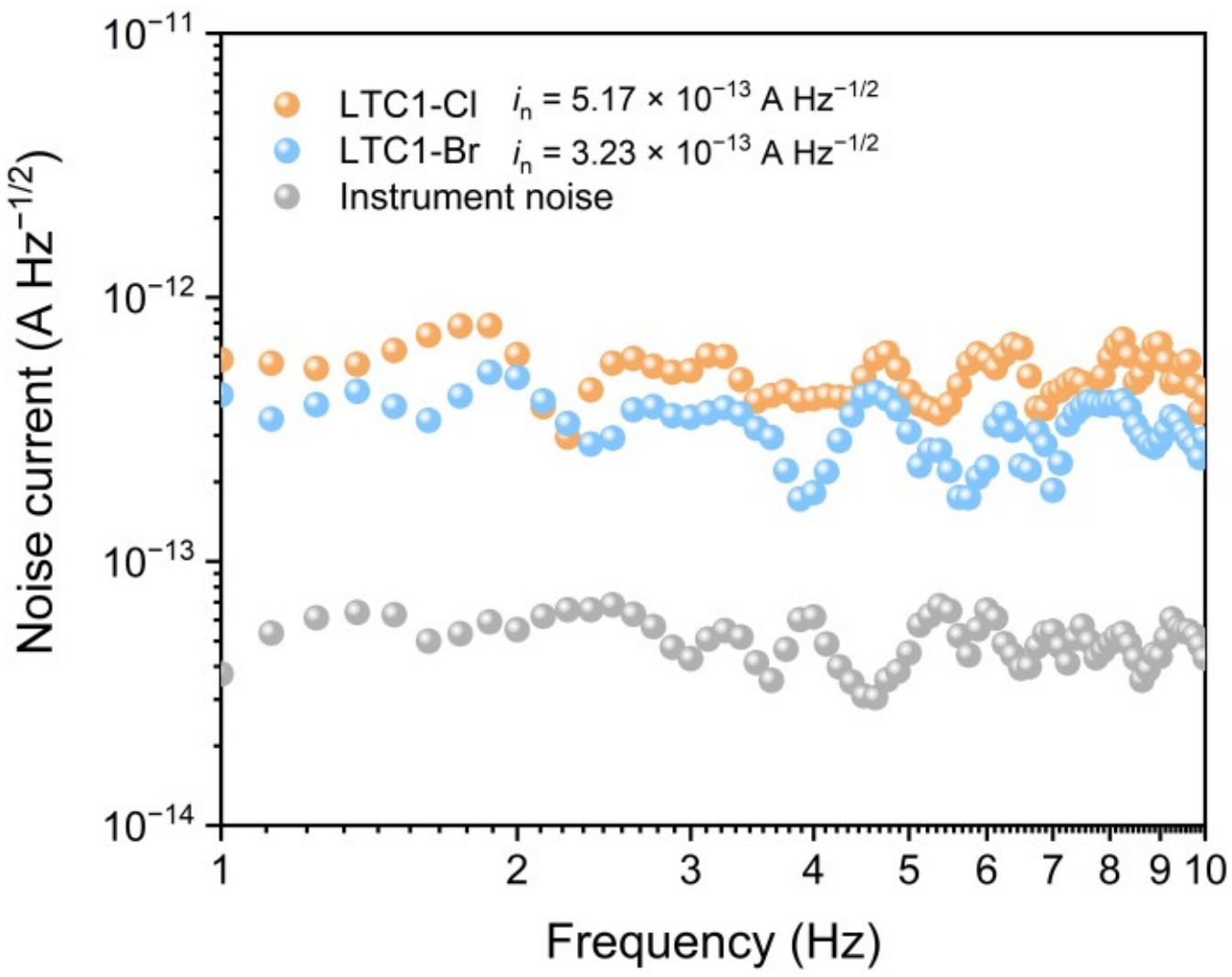

Fig. S31. The measured noise currents of SWIR OPDs based on PTB7-Th: LTC1-Cl and PTB7-Th: LTC1-Br under zero bias.

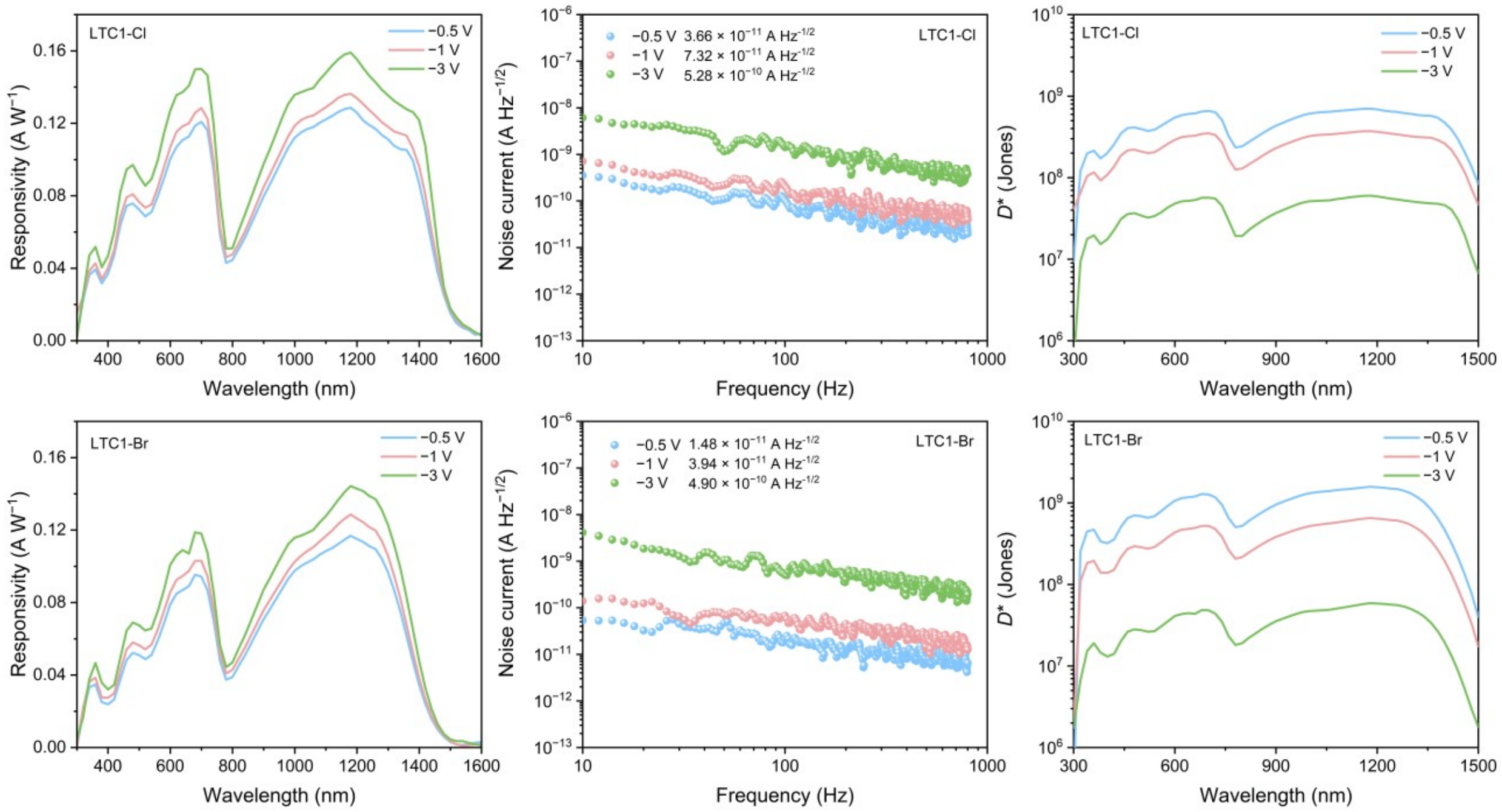

Fig. S32. Responsivity, noise current, and specific detectivity of SWIR OPDs based on PTB7-Th: LTC1-Cl and PTB7-Th: LTC1-Br under reverse bias conditions. The noise current used for D^* calculation was extracted at 210 Hz, consistent with the modulation frequency employed in EQE measurements.

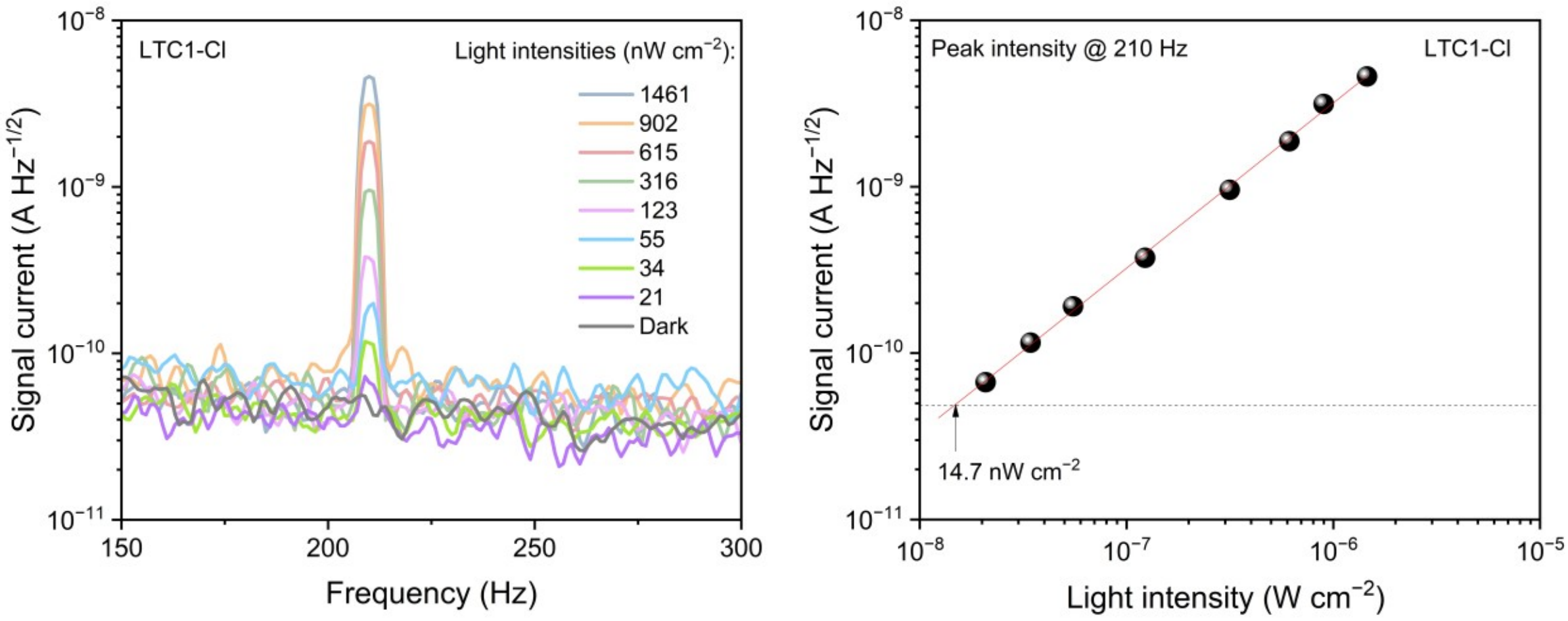

Fig. S33. Signal currents of PTB7-Th: LTC1-Cl-based SWIR OPDs measured under a reverse bias of −0.5 V and 1.3 μm light illumination modulated at 210 Hz over a range of light intensities, together with the corresponding peak signal intensities at 210 Hz as a function of light intensity.

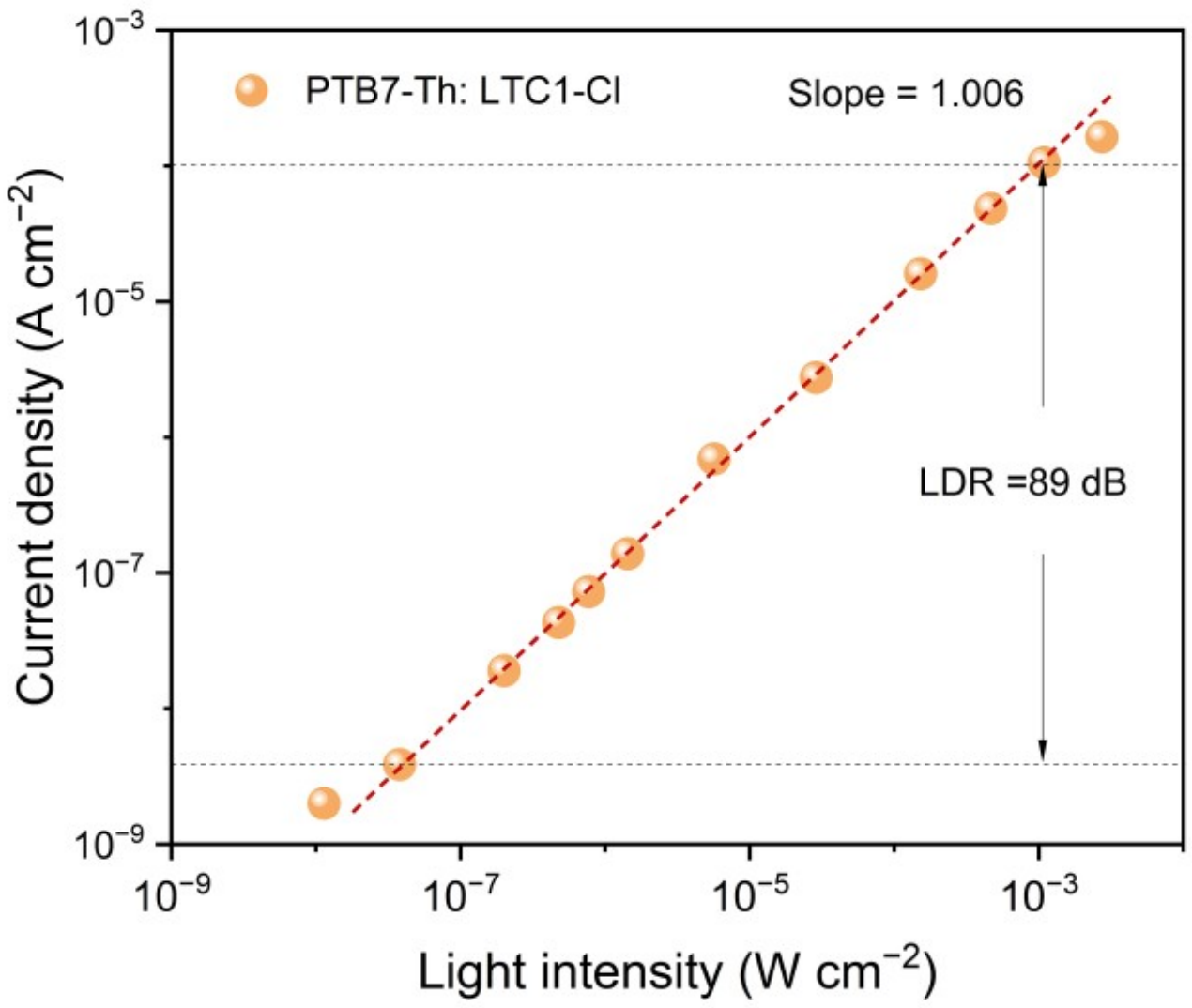

Fig. S34. LDR measurements of PTB7-Th: LTC1-Cl-based SWIR OPDs under a reverse bias of −0.5 V at 1.3 μm with various light intensities.

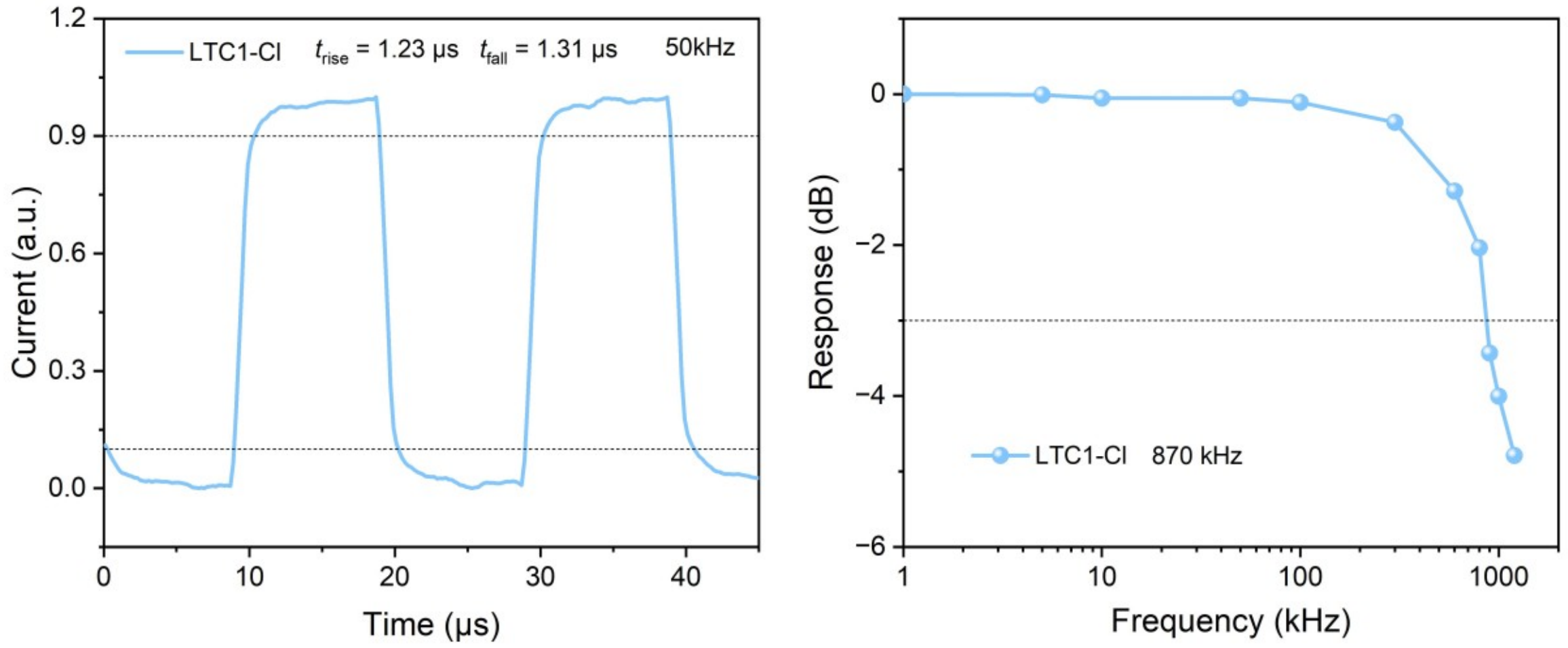

Fig. S35. The response time and cut-off frequency of OPDs based on PTB7-Th: LTC1-Cl under a reverse bias of −0.5 V and 1.3 μm LED illumination.

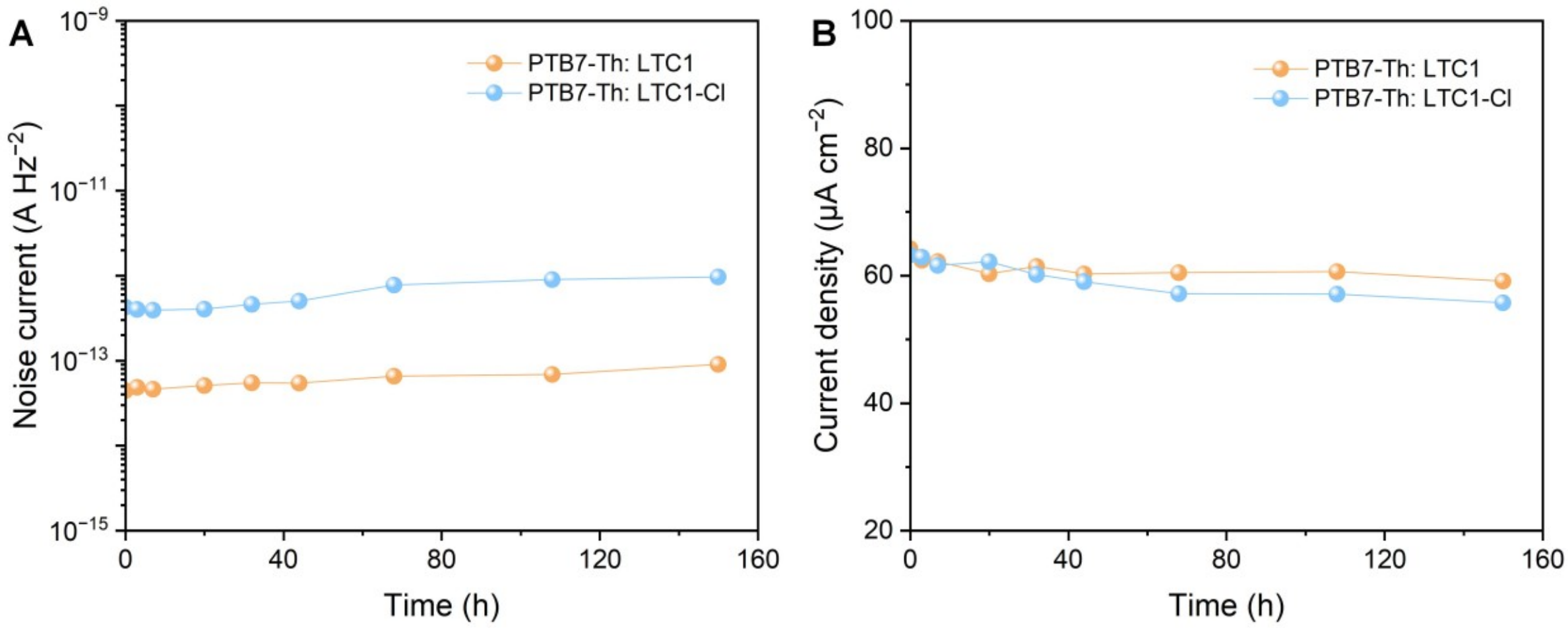

Fig. S36. Operational photostability of the PTB7-Th: LTC1- and PTB7-Th: LTC1-Cl-based OPD under continuous AM 1.5G irradiation at 1 mW cm^{-2} in N_2 atmosphere. (**A**) The noise currents in the dark at zero bias and (**B**) the photocurrent densities at zero bias under AM 1.5G irradiation at 1 mW cm^{-2}.

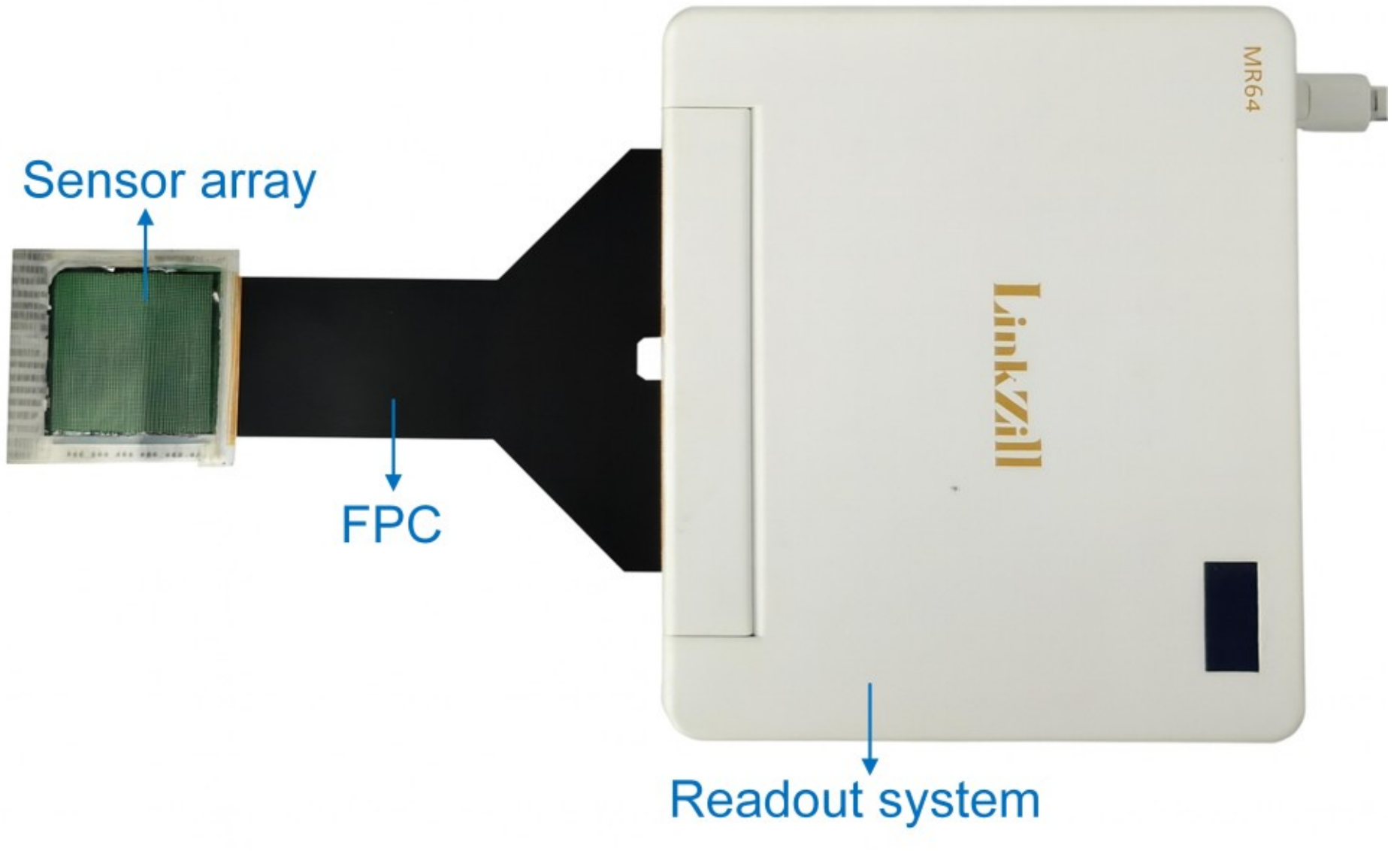

Fig. S37. A photograph of the active-matrix SWIR imaging system using an OPD-integrated TFT sensor array as the key element. FPC is flexible printed cables.

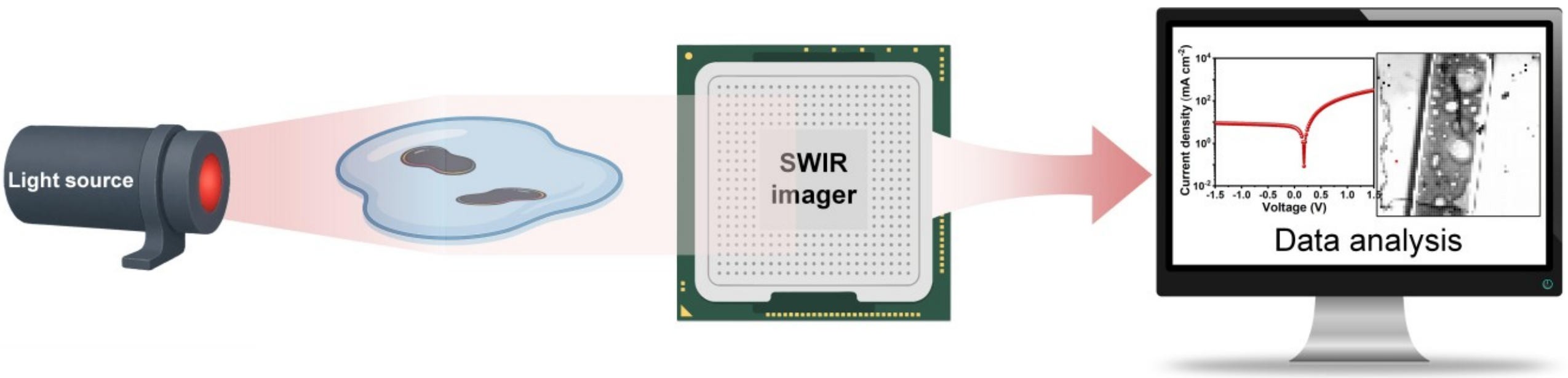

Fig. S38. Schematic diagram of the oil spill monitoring with the OPD-integrated SWIR sensor array.

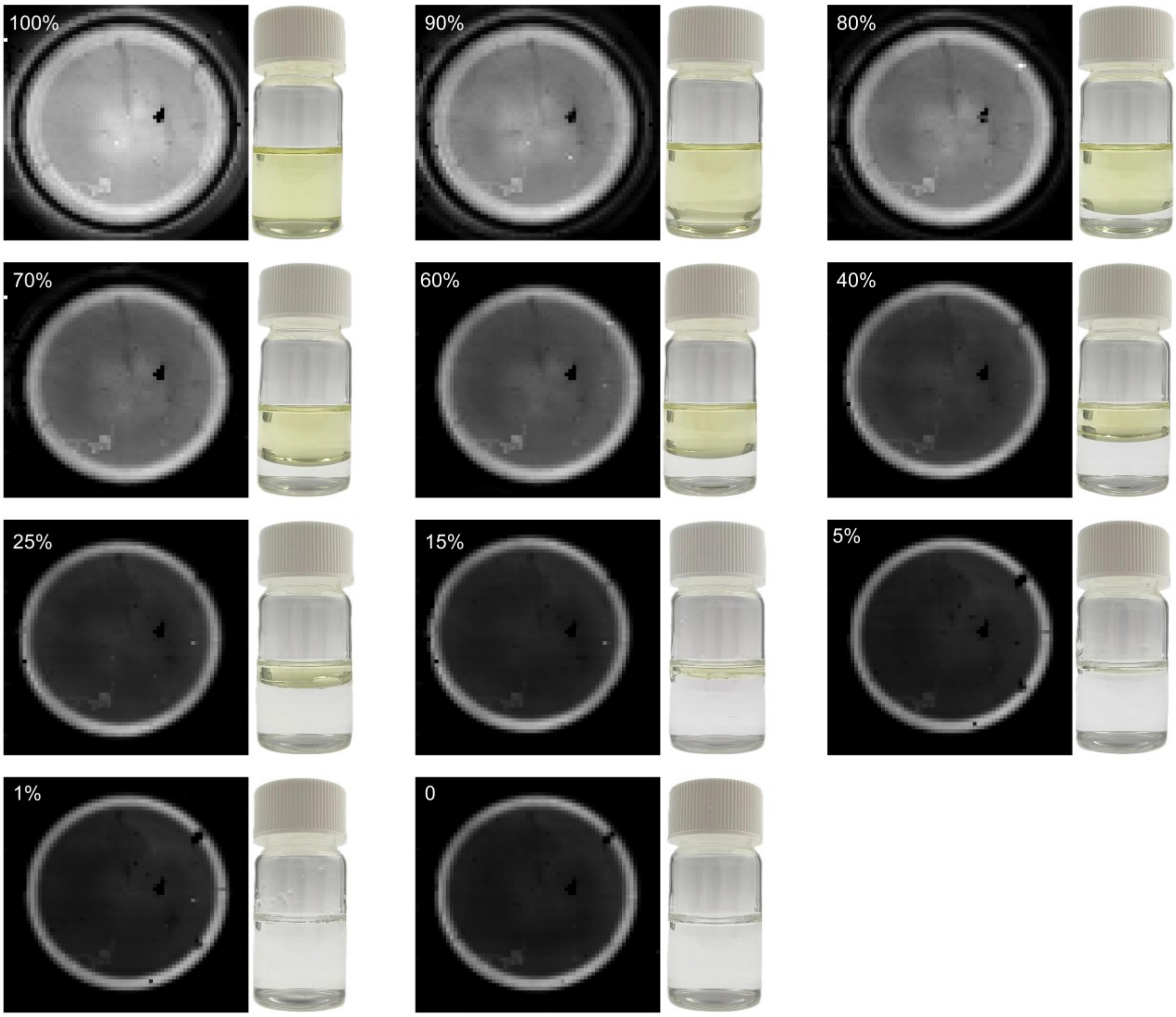

Fig. S39. SWIR imaging of oil-water mixtures with different oil fractions, along with the corresponding photographs. The SWIR images of oil-water mixtures with oil fractions of 90%, 60%, and 5% are also presented in Fig. 4B. The SWIR images were captured from an upward view after the container lid was removed, with an imaging area of 3.2 cm × 3.2 cm. All containers were 20 mL sample vials (body diameter: 2.7 cm) containing 10 mL of liquid in total.

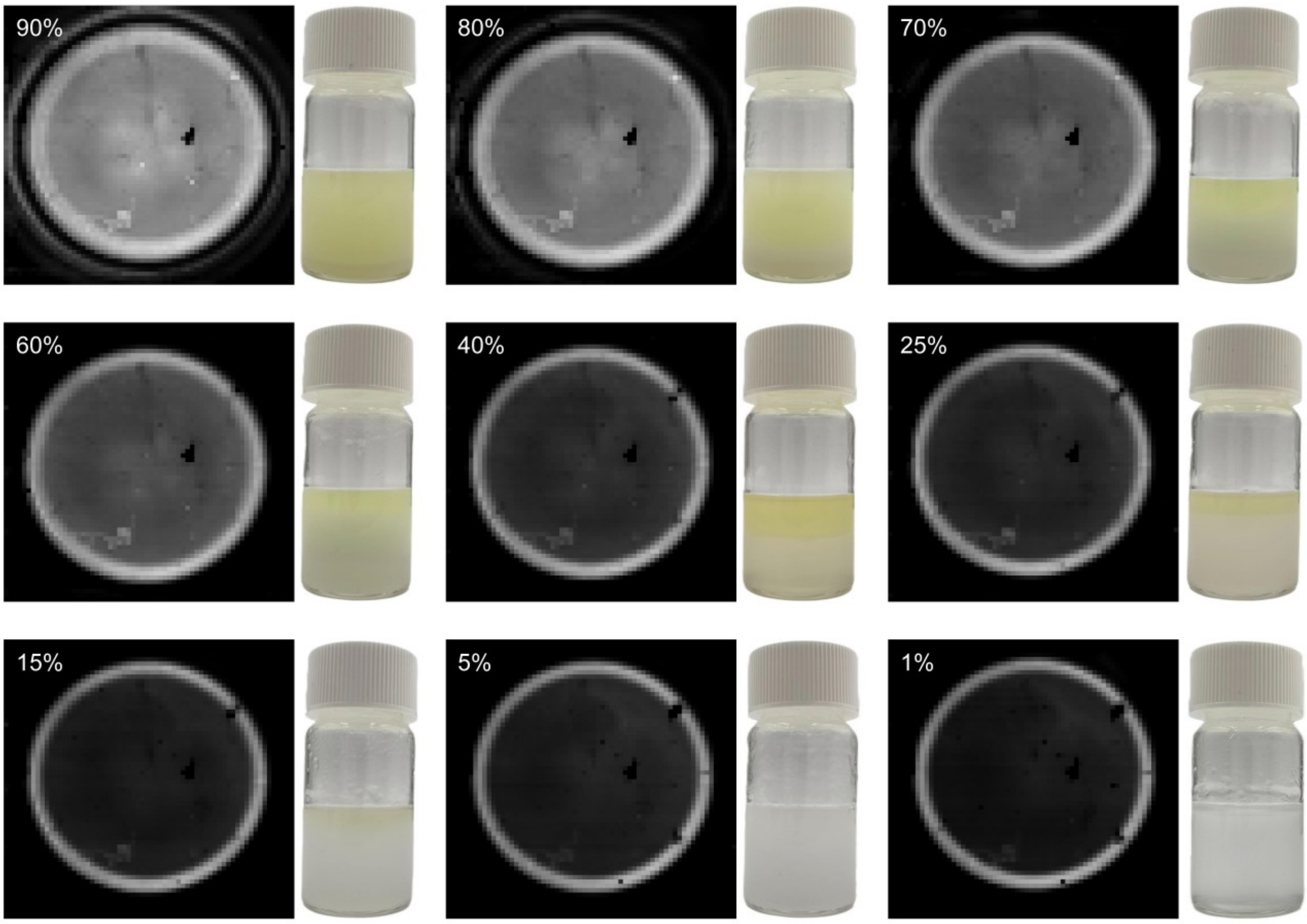

Fig. S40. SWIR imaging of oil–water mixtures with different oil fractions after 1 h emulsification, along with the corresponding photographs. The SWIR images of oil-water mixtures with oil fractions of 90%, 60%, and 5% are also presented in Fig. 4B. The SWIR images were captured from an upward view after the container lid was removed, with an imaging area of 3.2 cm × 3.2 cm. All containers were 20 mL sample vials (body diameter: 2.7 cm) containing 10 mL of liquid in total. Note: Although emulsification changes the dispersion state of the oil-water mixtures, the overall oil fraction remains unchanged relative to the corresponding initial mixtures. Therefore, the SWIR images before and after emulsification appear visually similar, with only subtle differences.

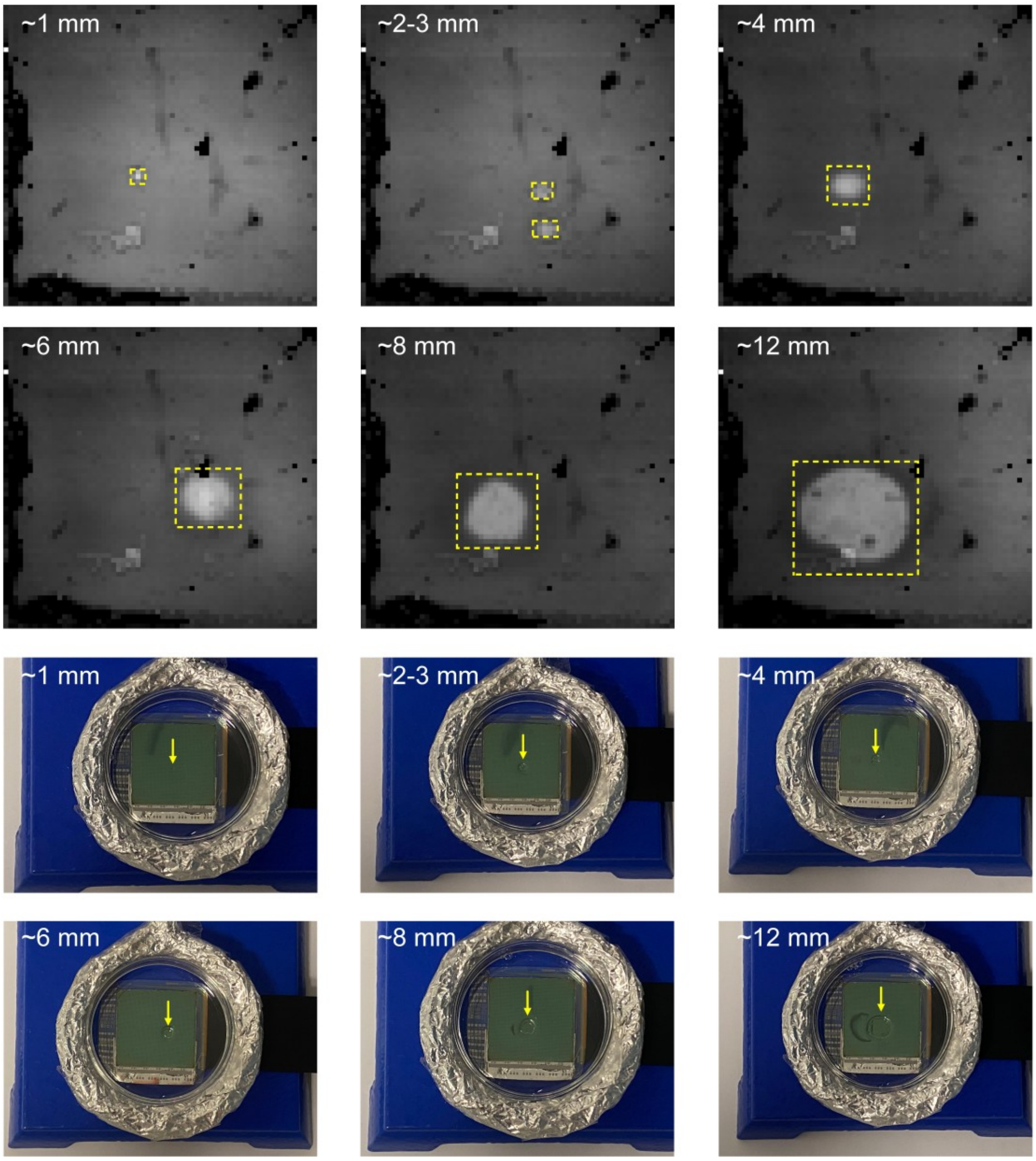

Fig. S41. Detection of oil droplets with diameters ranging from ~1 mm to ~12 mm under 1.4 µm illumination, shown with corresponding imaging and optical photographs. The images of oil droplets with diameters of ~1 mm, ~4 mm, and ~8 mm are also presented in Fig. 4D. The imaging area is 3.2 cm × 3.2 cm, and the samples are contained in culture dishes with a diameter of 6 cm, each holding 10 mL of solution.

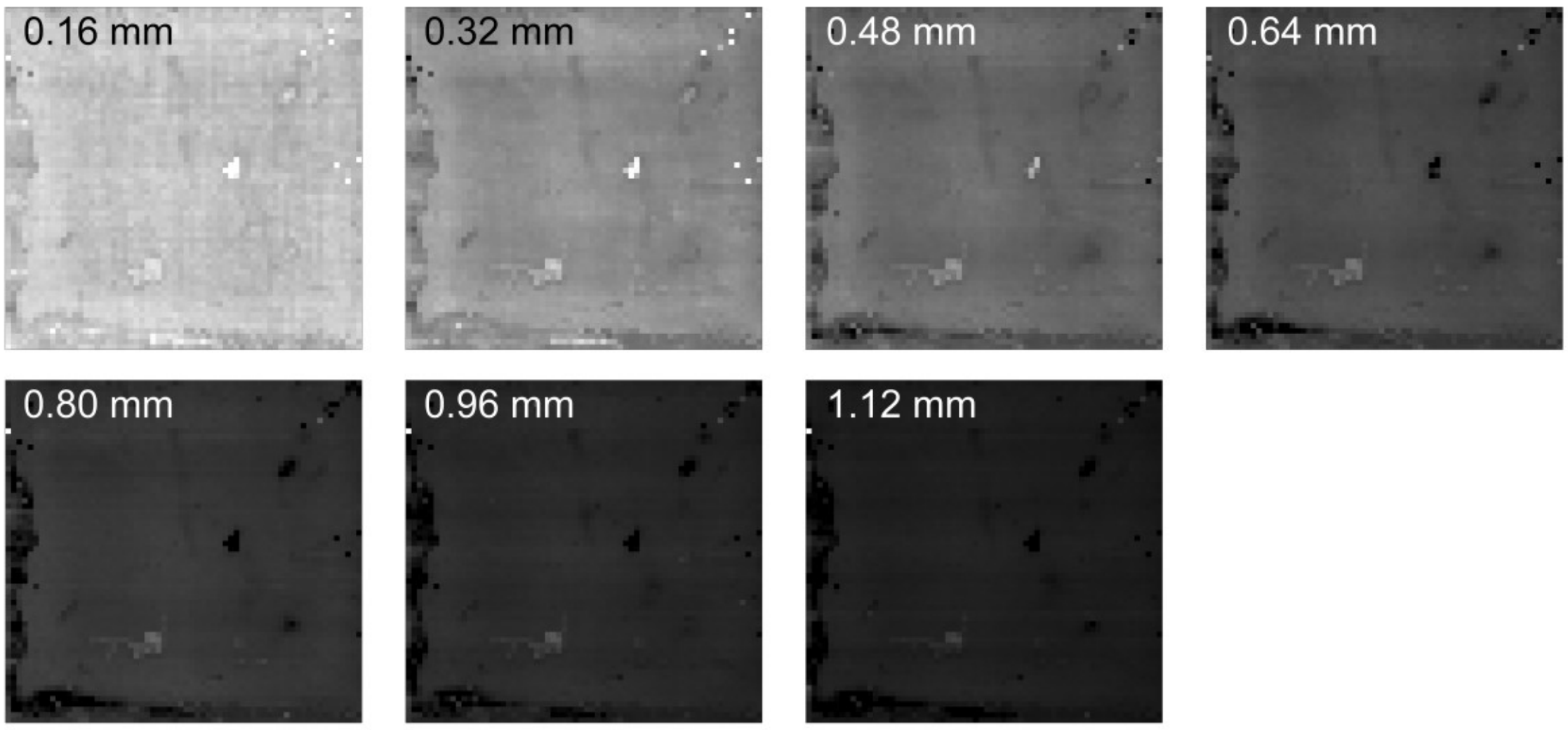

Fig. S42. SWIR imaging of 100% cotton fabrics with different thicknesses. The SWIR images of 100% cotton fabrics with thicknesses of 0.16 mm and 0.64 mm are also presented in Fig. 5B. The imaging area is 3.2 cm × 3.2 cm.

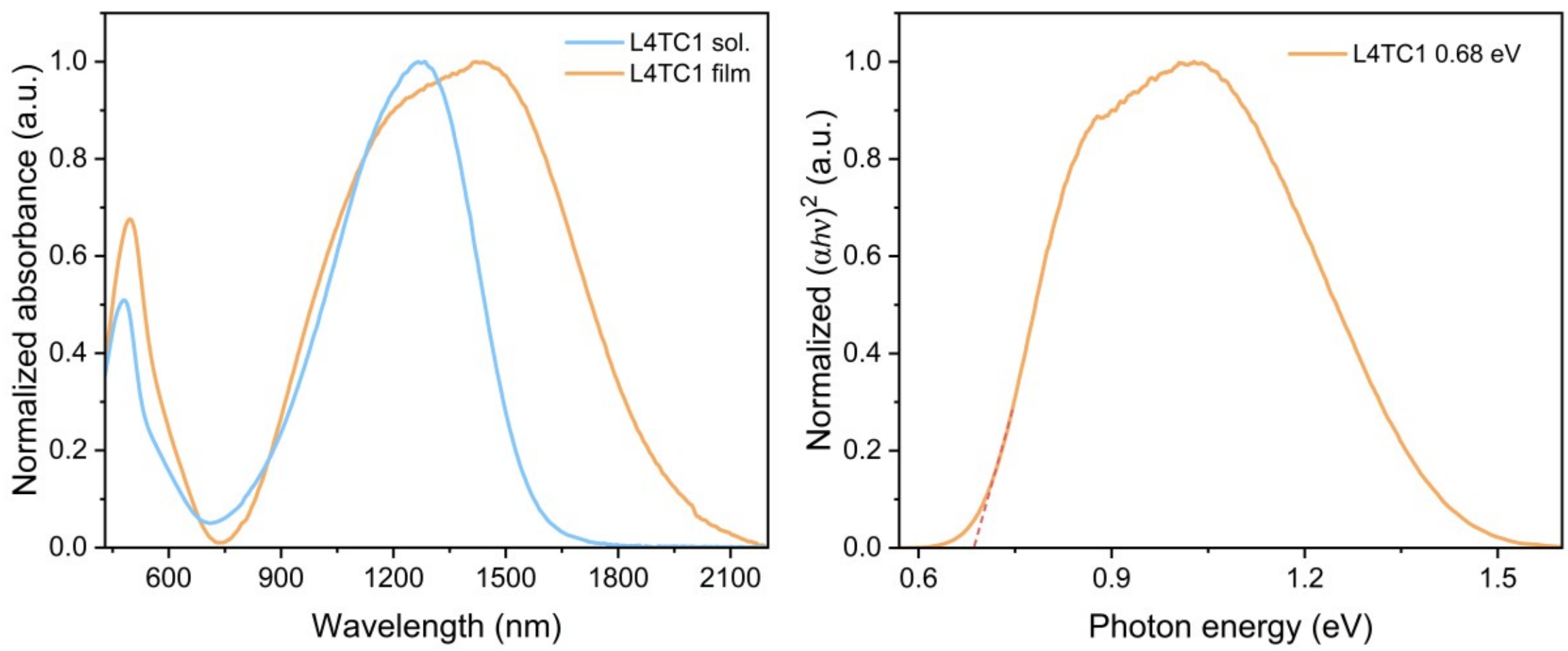

Fig. S43. Absorption spectra of L4TC1 in dilute chloroform solution and $CHCl_3$-cast thin films, and the corresponding E_g^{opt} value.

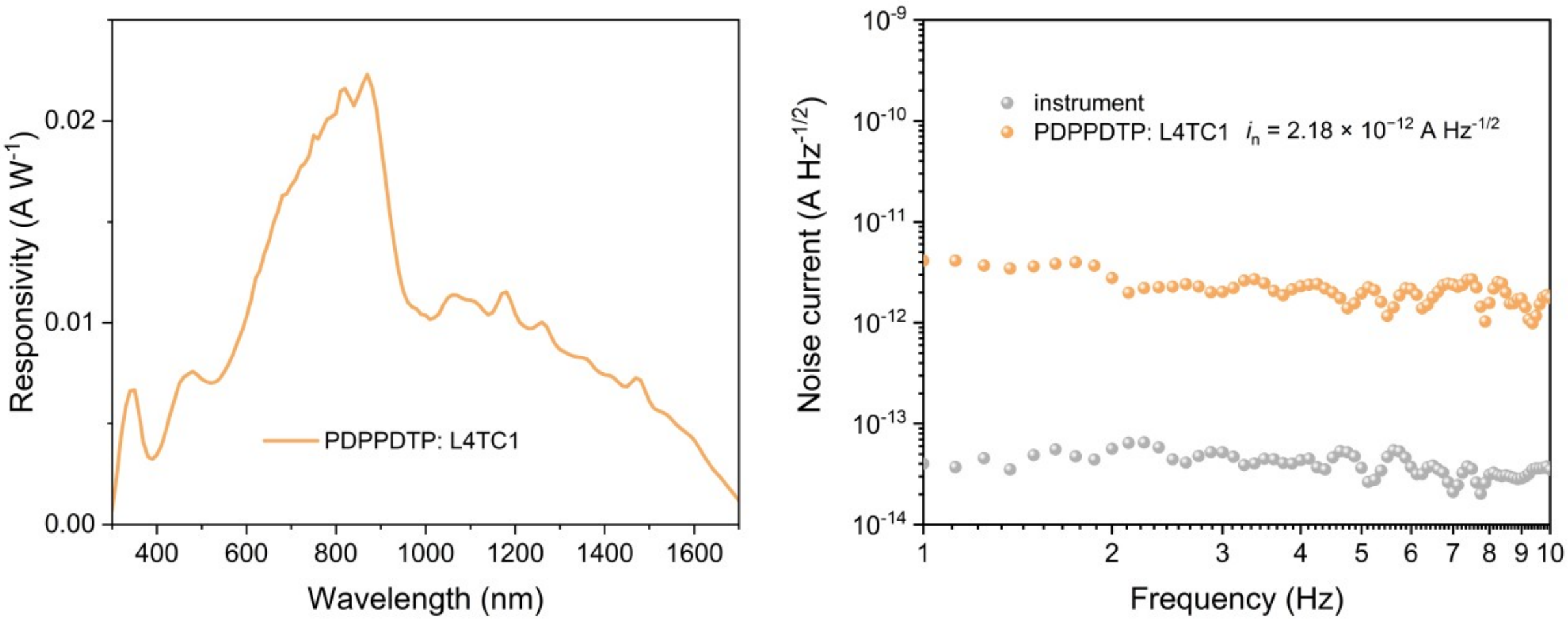

Fig. S44. The *R* and noise current curves of SWIR OPDs based on PDPPDTP: L4TC1 under zero bias.

Supplementary Tables

Table S1. Structure parameters acquired from GIWAXS measurements for neat films of L2-eC9 and LTCx (x = 1,2).

Film	Direction	Location (Å^{-1})	*d*-spacing (Å)	FWHM (Å^{-1})	CCL (Å)	Amorphous phase
L2-eC9	IP (100)	0.370	17.0	0.175	32.3	-
	OOP (010)	1.680	3.74	0.309	18.3	19.0%
LTC1	IP (100)	0.375	16.7	0.131	43.2	-
	OOP (010)	1.684	3.73	0.305	18.5	11.9%
LTC2	IP (100)	0.367	17.0	0.157	36.0	-
	OOP (010)	1.684	3.73	0.305	18.5	12.7%

Table S2. Crystal data of LTC1.

Identification code	LTC1
Temperature/K	99.97(11)
Crystal system	monoclinic
Space group	$P2_1/c$
a/Å	31.7242(4)
b/Å	15.3774(2)
c/Å	31.1026(3)
α/°	90
β/°	93.6660(10)
γ/°	90
Volume/$Å^3$	15141.9(3)
Z	4
F(000)	6200.0
Radiation	CuKα (λ = 1.54184)
Index ranges	$-39 \leq h \leq 39$, $-19 \leq k \leq 14$, $-38 \leq l \leq 39$
Goodness-of-fit on F^2	1.095
Final R indexes [I>=2σ (I)]	$R_1 = 0.1165$, $wR_2 = 0.2990$
Final R indexes [all data]	$R_1 = 0.1436$, $wR_2 = 0.3169$

Table S3. The summarized detection performance of reported SWIR OPDs with a response range exceeding 1.4 µm. Note that many reported D^* values in literature suppose that the noise power spectrum is "white" and calculated from the root mean square of i_{shot} and $i_{thermal}$ (even only using i_{shot} as the total noise current), generally leading to overestimated D^* values(*9*, *58*, *59*). Here, only D^* values calculated from real-measured i_n are summarized.

Photoactive Materials	Response limit (nm)	V_{bias} (V)	i_n (A Hz$^{-1/2}$)	Measured D^*_{max}† (Jones)	Ref.
PDPPDTP: L4	1600	0	1.0×10^{-12}	1.9×10^{9} @ 1.18 µm	(*15*)
PDPPDTP: BT-12D	1600	0	1.15×10^{-12}	~4×10^{9} @ 1 µm	(*53*)
PTTQ(HD): $PC_{71}BM$	1500	0	~1.2×10^{-13}	~1.7×10^{10} @ 1.14 µm	(*37*)
P1a: Y7	1500	−1	~5×10^{-12}	8.17×10^{9} @ 1.2 µm	(*60*)
P1: Y7	1500	−1	~1×10^{-12}	2.96×10^{10} @ 1.2 µm	(*60*)
P1b: Y7	1500	−1	~3×10^{-12}	~5.6×10^{9} @ 1.2 µm	(*60*)
TQ-T: IEICO-4F	1500	−2	~2×10^{-10}	~2.9×10^{8} @ 1.19 µm	(*19*)
TQ-3T: IEICO-4F	1500	−2	~6×10^{-12}	~1.1×10^{10} @ 1.14 µm	(*19*)
N2200/PPhTQ: COTIC-4F	1600	−0.2	~2×10^{-13}	~7.4×10^{10} @ 1 µm	(*61*)
PTB7-Th: L2-2Cl-eC9	1600	0	8.0×10^{-14}	2.11×10^{11} @ 1.03 µm	(*41*)
PTB7-Th: LTC1	1500	0	4.29×10^{-14}	4.53×10^{11} @ 1.06 µm	This work

†The values represent the maximum EQE and D^* in the SWIR region (1-3 µm).

Table S4. Structure parameters acquired from GIWAXS measurements for blend films of PTB7-Th: L2-eC9, and PTB7-Th: LTCx (x = 1, 2).

Film	Direction	Location (Å^{-1})	*d*-spacing (Å)	FWHM (Å^{-1})	CCL (Å)	Amorphous phase
PTB7-Th:L2-eC9	IP (100)	0.294	21.4	0.0868	65.1	-
	OOP (010)	1.672	3.80	0.311	18.2	46.7%
PTB7-Th:LTC1	IP (100)	0.296	21.2	0.0823	68.7	-
	OOP (010)	1.675	3.78	0.308	18.4	19.7%
PTB7-Th:LTC2	IP (100)	0.294	21.4	0.0848	66.7	-
	OOP (010)	1.675	3.79	0.309	18.3	20.7%

Supplementary Movie

Movie S1. Real-time monitoring of the dynamic motion and distribution of leaked diesel in flowing water under 1.4 μm illumination.

Supplementary Notes

Note S1. Effect of thiophene fusion orientation on energy levels

LTC3 differs from LTC1 and LTC2 by showing an upshifted LUMO (–3.83 eV) and a larger transport gap (1.51 eV) than L2-eC9 (fig. S3), which is attributed to the distinct thiophene orientation that disrupts intramolecular conjugation and reduces the charge transfer from fragment D to A (Q_{CT} = 0.593 electrons, fig. 2C), thereby weakening the ICT effect. To further rationalize these results at the orbital level, each molecule was divided into the terminal unit and the BTP-CN core (fig. S4). According to the symmetry-matching principle, fusion at the 1,2- or 3,4-position (or benzene) enables LUMO-LUMO interactions that generate lower-energy bonding orbitals serving as the LUMOs of LTC1, LTC2, and L2-eC9, whereas fusion at the 2,3-position causes HOMO-LUMO interactions that yield a higher-energy antibonding orbital, leading to the upshifted LUMO of LTC3 (fig. S5).

Note S2. GIWAXS analysis of intermolecular packing and aggregation

Intermolecular packing and aggregation were analyzed by GIWAXS (figs. S23, S24 and Table S4), which revealed that all blend films exhibit a face-on orientation with strong (010) and (100) diffraction peaks at ~1.67 $Å^{-1}$ (OOP) and ~0.29 $Å^{-1}$ (IP). The (100) peak CCLs of PTB7-Th: LTC1 and PTB7-Th: LTC2 (68.7 and 66.7 Å) exceed that of L2-eC9 (65.1 Å), indicating improved lamellar ordering, while their lower amorphous contents in the π-π stacking direction (19.7% and 20.7% vs. 46.7%) confirm that sulfur incorporation enhances intermolecular interactions and promotes more ordered π-π stacking.

Note S3. Analysis of trap density and charge carrier mobility

PTB7-Th: LTC1-based OPDs exhibit the lowest trap densities in the energy range of 0.12-0.18 eV (3.0×10^{15} to 8.4×10^{15} cm^{-3} eV^{-1}), followed by PTB7-Th: LTC2-based OPDs (3.5×10^{15} to 1.6×10^{16} cm^{-3} eV^{-1}), with PTB7-Th: L2-eC9-based OPDs showing the highest values (4.7×10^{15} to 6.2×10^{16} cm^{-3} eV^{-1}) (fig. S25). Due to its more ordered molecular packing and lower trap density, PTB7-Th: LTC1 exhibits faster and more balanced charge transport with μ_e, hole mobility (μ_h), and μ_h/μ_e of 8.4×10^{-5} cm^2 V^{-1} s^{-1}, 1.5×10^{-4} cm^2 V^{-1} s^{-1}, and 1.8, respectively, compared to those of PTB7-Th: LTC2 ($\mu_h = 1.4 \times 10^{-4}$ cm^2 V^{-1} s^{-1}, $\mu_e = 4.2 \times 10^{-5}$ cm^2 V^{-1} s^{-1}, $\mu_h/\mu_e = 3.3$) and PTB7-Th: L2-eC9 ($\mu_h = 7.7 \times 10^{-5}$ cm^2 V^{-1} s^{-1}, $\mu_e = 3.0 \times 10^{-5}$ cm^2 V^{-1} s^{-1}, $\mu_h/\mu_e = 2.6$) (fig. S26), contributing to the lowest bimolecular recombination rate constant of PTB7-Th: LTC1.

Note S4. Analysis of charge carrier dynamics

The charge recombination dynamics were investigated by measuring the open-circuit voltage (V_{OC}) and short-circuit current density (J_{SC}) under varying light intensities (P_{light}), revealing a relationship described by $V_{OC} \propto n(kT/q)\ln(P_{light})$ (*62*). As shown in fig. S27A, the slopes of V_{OC} versus $\ln(P_{light})$ are 0.97 kT/q for PTB7-Th: LTC1- and PTB7-Th: LTC2-based OPDs, closer to kT/q than the 0.92 kT/q of PTB7-Th:L2-eC9, indicating that bimolecular recombination dominates in all devices (*63–65*). The relationship $J_{SC} \propto P_{light}^{\alpha}$ further shows $\alpha = 0.94$ for PTB7-Th: LTC1 and PTB7-Th: L2-eC9, while a slightly lower $\alpha = 0.92$ is observed for PTB7-Th: LTC2 (fig. S27B), suggesting more pronounced bimolecular recombination in the LTC2-based device.

As shown in fig. S27C, PTB7-Th: LTC1-based OPDs exhibit the highest carrier concentration (22.93×10^{16}-8.91×10^{16} cm^{-3} across 4-100 mW cm^{-2}), exceeding that of PTB7-Th: L2-eC9 (1.69×10^{16}-8.50×10^{16} cm^{-3}), especially under low-light conditions, whereas PTB7-Th: LTC2-based OPDs show the lowest values (8.03×10^{15}-3.72×10^{16} cm^{-3} over 8-100 mW cm^{-2}). Carrier lifetime decreases with increasing concentration (fig. S27D), confirming bimolecular recombination. Correspondingly, the bimolecular recombination rate constants of PTB7-Th: LTC1 (2.02×10^{-12}-5.69×10^{-12} cm^3 s^{-1}) are lower than those of PTB7-Th: L2-eC9 (3.50×10^{-12}-6.57×10^{-12} cm^3 s^{-1}), particularly at low carrier concentrations, while PTB7-Th: LTC2-based OPDs show even higher constants ($>10^{-11}$ cm^3 s^{-1}), explaining their most pronounced recombination (fig. S27E). In addition, the carrier extraction times of PTB7-Th: LTC1- and PTB7-Th: LTC2-based OPDs are 0.39 and 0.54 μs, both shorter than that of PTB7-Th: L2-eC9 (0.83 μs) (fig. S27F), facilitating faster charge collection and consistent with improved response speed.

REFERENCES

1. Q. Duan, J. Li, Classification of common household plastic wastes combining multiple methods based on near-infrared spectroscopy. *ACS EST Engg.* **1**, 1065–1073 (2021).

2. L. G. Weyer, Near-infrared spectroscopy of organic substances. *Appl. Spectrosc. Rev.* **21**, 1–43 (1985).

3. THE 17 GOALS | Sustainable development. https://sdgs.un.org/goals.

4. J.-P. Lange, Managing plastic waste─sorting, recycling, disposal, and product redesign. *ACS Sustain. Chem. Eng.* **9**, 15722–15738 (2021).

5. J. Michel, J. Liu, L. C. Kimerling, High-performance Ge-on-Si photodetectors. *Nat. Photon.* **4**, 527–534 (2010).

6. F. Teng, K. Hu, W. Ouyang, X. Fang, Photoelectric detectors based on inorganic p-type semiconductor materials. *Adv. Mater.* **30**, e1706262 (2018).

7. Y. Chen, Y. Zheng, J. Wang, X. Zhao, G. Liu, Y. Lin, Y. Yang, L. Wang, Z. Tang, Y. Wang, Y. Fang, W. Zhang, X. Zhu, Ultranarrow-bandgap small-molecule acceptor enables sensitive SWIR detection and dynamic upconversion imaging. *Sci. Adv.* **10**, eadm9631 (2024).

8. C. Wang, X. Zhang, W. Hu, Organic photodiodes and phototransistors toward infrared detection: Materials, devices, and applications. *Chem. Soc. Rev.* **49**, 653–670 (2020).

9. C. Fuentes-Hernandez, W.-F. Chou, T. M. Khan, L. Diniz, J. Lukens, F. A. Larrain, V. A. Rodriguez-Toro, B. Kippelen, Large-area low-noise flexible organic photodiodes for detecting faint visible light. *Science* **370**, 698–701 (2020).

10. T. Kamijo, A. J. J. M. van Breemen, X. Ma, S. Shanmugam, T. Bel, G. de Haas, B. Peeters, R. Petre, D. Tordera, R. Verbeek, H. B. Akkerman, L. M. Hagelsieb, F. de Roose, I. Lieberman, F. Yamaguchi, R. A. J. Janssen, E. A. Meulenkamp, A. J. Kronemeijer, G. H. Gelinck, A touchless user interface based on a near-infrared-sensitive transparent optical imager. *Nat. Electron.* **6**, 451–461 (2023).

11. Y. Ma, X. Xu, T. Li, Z. Wang, N. Li, X. Zhao, W. Wei, X. Zhan, L. Shen, Amplified narrowband perovskite photodetectors enabled by independent multiplication layers for anti-interference light detection. *Sci. Adv.* **11**, eadq1127 (2025).

12. C. Li, H. Wang, F. Wang, T. Li, M. Xu, H. Wang, Z. Wang, X. Zhan, W. Hu, L. Shen, Ultrafast and broadband photodetectors based on a perovskite/organic bulk heterojunction for large-dynamic-range imaging. *Light Sci. Appl.* **9**, 31 (2020).

13. C. Yan, S. Barlow, Z. Wang, H. Yan, A. K.-Y. Jen, S. R. Marder, X. Zhan, Non-fullerene acceptors for organic solar cells. *Nat. Rev. Mater.* **3**, 18003 (2018).

14. D. Nodari, Z. Qiao, F. Furlan, O. J. Sandberg, K. Vandewal, N. Gasparini, Towards high and reliable specific detectivity in visible and infrared perovskite and organic photodiodes. *Nat. Rev. Mater.* **10**, 842–856 (2025).

15. T. Li, G. Hu, L. Tao, J. Jiang, J. Xin, Y. Li, W. Ma, L. Shen, Y. Fang, Y. Lin, Sensitive photodetection below silicon bandgap using quinoid-capped organic semiconductors. *Sci. Adv.* **9**, eadf6152 (2023).

16. Y. Zhang, J. Chen, J. Yang, M. Fu, Y. Cao, M. Dong, J. Yu, S. Dong, X. Yang, L. Shao, Z. Hu, H. Cai, C. Liu, F. Huang, Sensitive SWIR organic photodetectors with spectral response reaching 1.5 μm. *Adv. Mater.* **36**, e2406950 (2024).

17. J. Wang, Y. Xie, K. Chen, H. Wu, J. M. Hodgkiss, X. Zhan, Physical insights into non-fullerene organic photovoltaics. *Nat. Rev. Phys.* **6**, 365–381 (2024).

18. O. J. Sandberg, C. Kaiser, S. Zeiske, N. Zarrabi, S. Gielen, W. Maes, K. Vandewal, P. Meredith, A. Armin, Mid-gap trap state-mediated dark current in organic photodiodes. *Nat. Photonics* **17**, 368–374 (2023).

19. P. Jacoutot, A. D. Scaccabarozzi, D. Nodari, J. Panidi, Z. Qiao, A. Schiza, A. D. Nega, A. Dimitrakopoulou-Strauss, V. G. Gregoriou, M. Heeney, C. L. Chochos, A. A. Bakulin, N. Gasparini, Enhanced sub-1 eV detection in organic photodetectors through tuning polymer energetics and microstructure. *Sci. Adv.* **9**, eadh2694 (2023).

20. J. Xu, Y. Zhang, J. Liu, L. Wang, NIR-II absorbing monodispersed oligomers based on N-B←N unit. *Angew. Chem. Int. Ed. Engl.* **62**, e202310838 (2023).

21. B. Yin, X. Zhou, Y. Li, G. Hu, W. Wei, M. Yang, S. Jeong, W. Deng, B. Wu, Y. Cao, B. Huang, L. Pan, X. Yang, Z. Fu, Y. Fang, L. Shen, C. Yang, H. Wu, L. Lan, F. Huang, Y. Cao, C. Duan, Sensitive organic photodetectors with spectral response up to 1.3 μm using a quinoidal molecular semiconductor. *Adv. Mater.* **36**, e2310811 (2024).

22. W. Liu, W. Deng, W. Wang, H. Wu, C. Gao, Y. Xie, J. Zhao, X. Dong, Z. Zhao, Z. Zheng, Y. Chi, L. Duan, X. Zhan, Y. Zou, H. Wu, J. Peng, Y. Cao, Ultrahigh-radiance near-infrared organic light-emitting diodes. *Nat. Photonics* **19**, 650–657 (2025).

23. W. Guo, Y. Duan, W. Liu, X. Zhang, J. Gao, Z. You, Y. Jiang, Z. Ma, X. Zhan, Y. Liu, Ultranarrow bandgap electron acceptor with low reorganization energy and refined crystallization enables efficient self-powered photodetectors with spectral response over 1100 nm. *CCS Chem.* **7**, 1731–1744 (2024).

24. R. R. Birge, G. A. Schick, D. F. Bocian, Calculation of molecular polarizabilities using a semiclassical slater-type orbital-point dipole interaction (STOPDI) model. *J. Chem. Phys.* **79**, 2256–2264 (1983).

25. M. N. Bellido, On the polarizability of the sulfur atom. *Chem. Phys. Lett.* **122**, 562–566 (1985).

26. H. Yao, L. Ye, J. Hou, B. Jang, G. Han, Y. Cui, G. M. Su, C. Wang, B. Gao, R. Yu, H. Zhang, Y. Yi, H. Y. Woo, H. Ade, J. Hou, Achieving highly efficient nonfullerene organic solar cells with improved intermolecular interaction and open-circuit voltage. *Adv. Mater.* **29**, 1700254 (2017).

27. Y. Cui, P. Zhu, X. Shi, X. Liao, Y. Chen, Theoretical study of excited state charge transfer characteristics based on A–D–A and A–DA′D–A type nonfullerene acceptors. *J. Phys. Chem. C* **125**, 10250–10259 (2021).

28. S. Kuila, H. Miranda-Salinas, J. Eng, C. Li, M. R. Bryce, T. J. Penfold, A. P. Monkman, Rigid and planar π-conjugated molecules leading to long-lived intramolecular charge-transfer states exhibiting thermally activated delayed fluorescence. *Nat. Commun.* **15**, 9611 (2024).

29. S. Simon, M. Duran, J. J. Dannenberg, How does basis set superposition error change the potential surfaces for hydrogen-bonded dimers? *J. Chem. Phys.* **105**, 11024–11031 (1996).

30. S. Grimme, J. Antony, S. Ehrlich, H. Krieg, A consistent and accurate ab initio parametrization of density functional dispersion correction (DFT-D) for the 94 elements H-Pu. *J. Chem. Phys.* **132**, 154104 (2010).

31. G. Zhang, X.-K. Chen, J. Xiao, P. C. Y. Chow, M. Ren, G. Kupgan, X. Jiao, C. C. S. Chan, X. Du, R. Xia, Z. Chen, J. Yuan, Y. Zhang, S. Zhang, Y. Liu, Y. Zou, H. Yan, K. S. Wong, V. Coropceanu, N. Li, C. J. Brabec, J.-L. Bredas, H.-L. Yip, Y. Cao, Delocalization of exciton and electron wavefunction in non-fullerene acceptor molecules enables efficient organic solar cells. *Nat. Commun.* **11**, 3943 (2020).

32. C. W. Bunn, H. P. Klug, Chemical crystallography. *J. Phys. Chem.* **50**, 490–491 (1946).

33. C. Li, J. Zhou, J. Song, J. Xu, H. Zhang, X. Zhang, J. Guo, L. Zhu, D. Wei, G. Han, J. Min, Y. Zhang, Z. Xie, Y. Yi, H. Yan, F. Gao, F. Liu, Y. Sun, Non-fullerene acceptors with branched side chains and improved molecular packing to exceed 18% efficiency in organic solar cells. *Nat. Energy* **6**, 605–613 (2021).

34. Z. Zhang, Y. Li, G. Cai, Y. Zhang, X. Lu, Y. Lin, Selenium heterocyclic electron acceptor with small urbach energy for as-cast high-performance organic solar cells. *J. Am. Chem. Soc.* **142**, 18741–18745 (2020).

35. S. Karuthedath, J. Gorenflot, Y. Firdaus, N. Chaturvedi, C. S. P. De Castro, G. T. Harrison, J. I. Khan, A. Markina, A. H. Balawi, T. A. D. Peña, W. Liu, R.-Z. Liang, A. Sharma, S. H. K. Paleti, W. Zhang, Y. Lin, E. Alarousu, S. Lopatin, D. H. Anjum, P. M. Beaujuge, S. De Wolf, I. McCulloch, T. D. Anthopoulos, D. Baran, D. Andrienko, F. Laquai, Intrinsic efficiency limits in low-bandgap non-fullerene acceptor organic solar cells. *Nat. Mater.* **20**, 378–384 (2021).

36. G. Yu, J. Gao, J. C. Hummelen, F. Wudl, A. J. Heeger, Polymer photovoltaic cells: Enhanced efficiencies via a network of internal donor-acceptor heterojunctions. *Science* **270**, 1789–1791 (1995).

37. S. Gielen, C. Kaiser, F. Verstraeten, J. Kublitski, J. Benduhn, D. Spoltore, P. Verstappen, W. Maes, P. Meredith, A. Armin, K. Vandewal, Intrinsic detectivity limits of organic near-infrared photodetectors. *Adv. Mater.* **32**, e2003818 (2020).

38. Z. Wu, N. Li, N. Eedugurala, J. D. Azoulay, D.-S. Leem, T. N. Ng, Noise and detectivity limits in organic shortwave infrared photodiodes with low disorder. *npj Flex. Electron.* **4**, 6 (2020).

39. X. Li, Q. Zhang, J. Yu, Y. Xu, R. Zhang, C. Wang, H. Zhang, S. Fabiano, X. Liu, J. Hou, F. Gao, M. Fahlman, Mapping the energy level alignment at donor/acceptor interfaces in non-fullerene organic solar cells. *Nat. Commun.* **13**, 2046 (2022).

40. J. B. Barton, R. F. Cannata, S. M. Petronio, InGaAs NIR focal plane arrays for imaging and DWDM applications. *Proc. SPIE* **4721**, 37–47 (2002).

41. T. Li, Y. Qu, Y. Peng, H. Liu, Y. Yang, J. Zhang, H. Hou, L. Li, Q. Lin, X. Lu, Y. Yang, Y. Fang, Y. Lin, Organic semiconductors with halogenated quinoid terminals for sensitive short-wave infrared detection and imaging. *Adv. Mater.* **38**, e22336 (2026).

42. D. Geng, K. Wang, L. Li, K. Myny, A. Nathan, J. Jang, Y. Kuo, M. Liu, Thin-film transistors for large-area electronics. *Nat. Electron.* **6**, 963–972 (2023).

43. S. E. Chang, Potential impacts of an impending oil spill. *Nat. Sustain.* **4**, 1023–1024 (2021).

44. M. Xie, Y. Li, Z. Zhang, Q. Fu, H. Jiang, Remote sensing of the oil spills caused by ships: A review. *Mar. Pollut. Bull.* **214**, 117754 (2025).

45. H. Pan, K. Tang, J. Zhuo, Y. Lu, J. Chen, Z. Lv, Underwater acoustic technology-based monitoring of oil spill: A review. *J. Mar. Sci. Eng.* **11**, 870 (2023).

46. S. Y. Venyaminov, F. G. Prendergast, Water (H_2O and D_2O) molar absorptivity in the 1000–4000 cm^{-1} range and quantitative infrared spectroscopy of aqueous solutions. *Anal. Biochem.* **248**, 234–245 (1997).

47. Textiles strategy-european commission (An official website of the European Union, 2025); https://environment.ec.europa.eu/strategy/textiles-strategy_en.

48. J.-R. Riba, R. Cantero, T. Canals, R. Puig, Circular economy of post-consumer textile waste: Classification through infrared spectroscopy. *J. Clean. Prod.* **272**, 123011 (2020).

49. A. Rose, The sensitivity performance of the human eye on an absolute scale. *J. Opt. Soc. Am.* **38**, 196–208 (1948).

50. K. H. Hendriks, W. Li, M. M. Wienk, R. A. J. Janssen, Small-bandgap semiconducting polymers with high near-infrared photoresponse. *J. Am. Chem. Soc.* **136**, 12130–12136 (2014).

51. B. L. Chenard, J. R. McConnell, J. S. Swenton, An efficient route to benzo[b]thiophenequinones, quinone bisketals, and quinone monoketals via anodic oxidation of methoxylated benzo[b]thiophenes. Studies directed at the synthesis of functionalized benzo[b]thiophene-4,7-quinones. *J. Org. Chem.* **48**, 4312–4317 (1983).

52. Z. Jiang, N. Liu, G. Dong, Y. Jiang, Y. Liu, X. He, Y. Huang, S. He, W. Chen, Z. Li, J. Yao, Z. Miao, W. Zhang, C. Sheng, Scaffold hopping of sampangine: Discovery of potent antifungal lead compound against Aspergillus fumigatus and Cryptococcus neoformans. *Bioorg. Med. Chem. Lett.* **24**, 4090–4094 (2014).

53. Z. Zhang, Y. Yang, T. Li, H. Hou, Y. Geng, Q. Lin, Y. Lin, Deuterating narrow-bandgap organic semiconductors for sensitive short-wave infrared photodetection. *Angew. Chem. Int. Ed. Engl.* **64**, e202507637 (2025).

54. M. J. Frisch, G. W. Trucks, H. B. Schlegel, G. E. Scuseria, M. A. Robb, J. R. Cheeseman, G. Scalmani, V. Barone, G. A. Petersson, H. Nakatsuji, X. Li, M. Caricato, A. V. Marenich, J. Bloino, B. G. Janesko, R. Gomperts, B. Mennucci, H. P. Hratchian, J. V. Ortiz, A. F.

Izmaylov, J. L. Sonnenberg, D. Williams-Young, F. Ding, F. Lipparini, F. Egidi, J. Goings, B. Peng, A. Petrone, T. Henderson, D. Ranasinghe, V. G. Zakrzewski, J. Gao, N. Rega, G. Zheng, W. Liang, M. Hada, M. Ehara, K. Toyota, R. Fukuda, J. Hasegawa, M. Ishida, T. Nakajima, Y. Honda, O. Kitao, H. Nakai, T. Vreven, K. Throssell, J. A. Montgomery Jr., J. E. Peralta, F. Ogliaro, M. J. Bearpark, J. J. Heyd, E. N. Brothers, K. N. Kudin, V. N. Staroverov, T. A. Keith, R. Kobayashi, J. Normand, K. Raghavachari, A. P. Rendell, J. C. Burant, S. S. Iyengar, J. Tomasi, M. Cossi, J. M. Millam, M. Klene, C. Adamo, R. Cammi, J. W. Ochterski, R. L. Martin, K. Morokuma, O. Farkas, J. B. Foresman, D. J. Fox, Gaussian 16, Revision A.03, Gaussian, Inc., Wallingford CT, (2016).

55. J. B. Johnson, Thermal agitation of electricity in conductors. *Phys. Rev.* **32**, 97–109 (1928).

56. G. G. Malliaras, J. R. Salem, P. J. Brock, C. Scott, Electrical characteristics and efficiency of single-layer organic light-emitting diodes. *Phys. Rev. B* **58**, R13411–R13414 (1998).

57. R. A. Street, Y. Yang, B. C. Thompson, I. McCulloch, Capacitance spectroscopy of light induced trap states in organic solar cells. *J. Phys. Chem. C* **120**, 22169–22178 (2016).

58. Y. Fang, A. Armin, P. Meredith, J. Huang, Accurate characterization of next-generation thin-film photodetectors. *Nat. Photon.* **13**, 1–4 (2019).

59. V. Pecunia, T. D. Anthopoulos, A. Armin, B. Bouthinon, M. Caironi, A. Castellanos-Gomez, Y. Chen, K. Cho, C. Clegg, X. Fang, P. Fendel, B. Fowler, G. Gelinck, H. Gottlob, P. Guyot-Sionnest, R. Hannebauer, G. Hernandez-Sosa, M. C. Hersam, L. Hirsch, J. C. Ho, F. H. Isikgor, J. Joimel, H. J. Kim, G. Konstantatos, J. Labram, M. C. Lemme, K. Leo, E. Lhuillier, E. Lidorikis, M. A. Loi, P. E. Malinowski, P. Merken, T. Mueller, B. Nasrollahi, D. Natali, T. N. Ng, T.-Q. Nguyen, S. K. Park, L.-M. Peng, P. Samorì, E. H. Sargent, L. Shen, S. Shishido, I. Shorubalko, P. Sonar, S. D. Stranks, S. F. Tedde, K. Vandewal, M. Verhaegen, S. Walia, F. Yan, T. Yokota, F. Zhang, Guidelines for accurate evaluation of photodetectors based on emerging semiconductor technologies. *Nat. Photon.* **19**, 1178–1188 (2025).

60. I. Park, C. Kim, R. Kim, N. Li, J. Lee, O. K. Kwon, B. Choi, T. N. Ng, D.-S. Leem, High performance shortwave infrared organic photodetectors adopting thiadiazole quinoxaline-based copolymers. *Adv. Opt. Mater.* **10**, 2200747 (2022).

61. X. Yu, H. Lin, Z. He, X. Du, Z. Chen, G. Yang, C. Zheng, S. Tao, Efficient near-infrared organic photodetectors with spectral response up to 1600 nm for accurate alcohol concentration detection. *ACS Appl. Mater. Interfaces* **15**, 16918–16929 (2023).

62. H. Lu, W. Liu, G. Ran, Z. Liang, H. Li, N. Wei, H. Wu, Z. Ma, Y. Liu, W. Zhang, X. Xu, Z. Bo, High-pressure fabrication of binary organic solar cells with high molecular weight D18 yields record 19.65 % efficiency. *Angew. Chem. Int. Ed. Engl.* **62**, e202314420 (2023).

63. L. J. A. Koster, V. D. Mihailetchi, R. Ramaker, P. W. M. Blom, Light intensity dependence of open-circuit voltage of polymer:Fullerene solar cells. *Appl. Phys. Lett.* **86**, 123509 (2005).

64. S. R. Cowan, A. Roy, A. J. Heeger, Recombination in polymer-fullerene bulk heterojunction solar cells. *Phys. Rev. B* **82**, 245207 (2010).

65. S. Wheeler, F. Deledalle, N. Tokmoldin, T. Kirchartz, J. Nelson, J. R. Durrant, Influence of surface recombination on charge-carrier kinetics in organic bulk heterojunction solar cells with nickel oxide interlayers. *Phys. Rev. Appl.* **4**, 024020 (2015).
